# Future Climate Change and Anthropogenic Disturbance Promote the Invasions of the World’s Worst Invasive Insect Pests

**DOI:** 10.3390/insects15040280

**Published:** 2024-04-16

**Authors:** Runyao Cao, Jianmeng Feng

**Affiliations:** College of Agriculture and Biological Science, Dali University, Dali 671003, China; cao1998424@163.com

**Keywords:** anthropogenic disturbances, climate change, future scenarios, range dynamics, worst invasive insect pests

## Abstract

**Simple Summary:**

Invasive insect pests adversely impact human welfare and global ecosystems. However, no studies have compared the future invasion risk of invasive insect pests using a unified scheme. We compared the future range dynamics of 15 of the world’s worst invasive insect pests. Although future range dynamics varied substantially among the 15 worst invasive insect pests, most exhibited large range expansions. The Asian tiger mosquito, cypress aphid, and Khapra beetle occurred four times in the top five largest potential ranges under most of the four climate scenarios examined. The Asian longhorned beetle, Asian tiger mosquito, and Formosan subterranean termite were predicted to have the largest range expansions. The Asian longhorned beetle, New Guinea flatworm, Formosan subterranean termite, and red imported fire ant exhibited the largest range centroid shifts. Strategies for their detection and management should be species-specific. This study furthers the understanding of how habitat changes may affect the ecology and shifts in the invasive potential of these insects.

**Abstract:**

Invasive insect pests adversely impact human welfare and global ecosystems. However, no studies have used a unified scheme to compare the range dynamics of the world’s worst invasive insect pests. We investigated the future range shifts of 15 of the world’s worst invasive insect pests. Although future range dynamics varied substantially among the 15 worst invasive insect pests, most exhibited large range expansions. Increases in the total habitat suitability occurred in more than ca. 85% of global terrestrial regions. The relative impacts of anthropogenic disturbance and climate variables on the range dynamics depended on the species and spatial scale. *Aedes albopictus*, *Cinara cupressi*, and *Trogoderma granarium* occurred four times in the top five largest potential ranges under four future climate scenarios. *Anoplophora glabripennis*, *Aedes albopictus*, and *Co. formosanus* were predicted to have the largest range expansions. *An. glabripennis*, *Pl. manokwari*, *Co. formosanus*, and *So. invicta* showed the largest range centroid shifts. More effective strategies will be required to prevent their range expansions. Although the strategies should be species-specific, mitigating anthropogenic disturbances and climate change will be essential to preventing future invasions. This study provides critical and novel insights for developing global strategies to combat the invasions of invasive insect pests in the future.

## 1. Introduction

Invasive insect pests (*IIPs*) have adversely affected human health, food supplies, beneficial species, ecosystem functions, and the economy globally [1,2,3]. For example, the spongy moth (*Lymantria dispar*) is one of the world’s worst *IIPs* and has caused significant defoliation and damage to native and commercial forest ecosystems [4,5,6]. Additionally, the impacts of *IIPs* are expected to intensify, primarily due to climate change and the accelerating pace of globalization, potentially exacerbating the spread of *IIPs* globally [3,7,8]. Predicting the occurrence of *IIPs* is essential to mitigating this problem, identifying invasion hotspots, and improving the strategies to prevent their invasions [3,9,10]. Therefore, predicting the potential ranges of *IIPs* is critical [11,12]. Although hundreds of *IIPs* exist, the worst *IIPs*,such as the Argentine ant (*Linepithema humile*), Asian gypsy moth (*Lymantria dispar*), and Asian longhorned beetle (*Anoplophora glabripennis*), should be the top-priority target species. To the best of our knowledge, no studies have assessed the range dynamics of the worst *IIPs* using a unified scheme to compare their invasion risk.

Numerous studies have indicated the strong impacts of climate change on altering the distribution of *IIPs.* Winter warming might contribute to expanding the distribution of *IIPs* [13]. For example, Justine et al. (2014) projected that winter warming might expand the range of the flatworm (*Platydemus manokwari*), one of the world’s worst *IIPs* [14]. Nunez-Mir et al. (2022) projected a winter-warming-induced range expansion of the European spongy moth (*L. dispar dispar*) in North America [15]. Although many studies have found that future climate change could cause range expansions of *IIPs* [16,17,18], controversy remains. For example, Netherer and Schopf, A. (2010) projected the future range contraction of *Thaumetopoea pityocampa*, an important pine defoliator [18], whereas Battisti et al. (2017) projected climate-change-induced range expansions of this species [19]. Moreover, Olabimi et al. (2021) argued that the range dynamics of an *IIP* might be affected by the general circulation models (GCMs) used to calibrate climate conditions under future scenarios [20]. Thus, the impacts of climate change on *IIPs*’ range dynamics require more investigation.

In addition to the effects of climate change, anthropogenic disturbance, such as land use (cover) change, could substantially influence the *IIP* ranges due to habitat modification and food availability [7,21,22]. For example, Munga et al. (2009) observed that anthropogenic disturbance could modify the distribution of *Anopheles gambiae* [23]. Liu et al. (2019) found that anthropogenic disturbance considerably affected the future ranges of *IIPs*, i.e., *Aedes aegypti* and *Ae. albopictus* [24]. Although studies have demonstrated the strong influence of anthropogenic disturbance on *IIP* ranges, its effects relative to climate change remain unclear [25]. For example, Ageep et al. (2009) observed that anthropogenic disturbance had stronger impacts on the range dynamics of *IIPs* than climate change [26]. In contrast, Nie et al. (2023) projected the stronger roles of climate change than anthropogenic disturbance on the range dynamics of *Hyphantria cunea* in Europe [27]. Therefore, more research is required on the relative influences of climate change and anthropogenic disturbance on *IIP* ranges.

The influences of topography on *IIP* ranges have attracted increased attention. Topographical factors are related to insect habitat [28,29] and represent barriers to species dispersal [30]. For example, Azrag et al. (2018) detected strong influences of elevation on the distribution of the coffee stink bug (*Antestiopsis thunbergia*) in Tanzania [31]. Adeogun et al. (2023) observed the influences of topographical factors on the ranges of several *IIPs* in Nigeria [32]. However, the influences of topographical factors relative to those of climate variables need further investigation. For example, Macedo et al. (2023) observed that topographical factors had strong effects on *Drosophila suzukii*’ ranges in Madeira Island in Portugal [33]. In contrast, de Souza et al. (2010) [34] and Adeogun et al. (2023) [32] did not observe this. Hence, the relative impacts of topographical and climatic variables on *IIPs*’ ranges require more study.

Although many studies have investigated the effects of several factors on the range dynamics of *IIPs* in recent decades, and significant progress has been made, these studies lacked a unified scheme and did not compare the range dynamics of *IIPs*. The likely reason is that different predictors and indices were used to quantify the magnitude of range shifts [5,6,35,36]. Therefore, it remains unknown which factors have the largest effects on the range dynamics and invasion risk of *IIPs*, and which *IIPs* might have larger invasion potentials in the future. For example, Peterson et al. (2007) used topographical and climatic predictors to project the range dynamics of *Lymantria dispar*, one of the worst *IIPs* [37]. In contrast, Jung et al. (2022) only utilized climatic variables to predict the future range shifts in one of the worst *IIPs*: *Linepithema humile* [38]. Additionally, Nie and Feng (2023) determined the range expansions, range contractions, and stable ranges to assess the future range dynamics of *Aedes albopictus*, one of the worst *IIPs* [39]. Kraemer et al. (2015) investigated range dynamics by comparing the spatial patterns of potential ranges under current and future scenarios [40]. Therefore, investigations on the range dynamics of the worst *IIPs* using a unified or comparable scheme are urgently needed.

Here, we hypothesized that the relative impacts of topography, anthropogenic disturbance, and climate on the range dynamics of the worst *IIPs* might depend on the invading insect species. The objective was to elucidate the relative risk of range dynamics of the 15 worst *IIPs* under current and future scenarios. We used our recently developed range dynamic models and topography, anthropogenic disturbance, and climate change datasets to investigate the range dynamics of the 15 worst *IIPs* and the influencing factors. This study is expected to provide crucial information for devising strategies to mitigate future invasions of the worst *IIPs*.

## 2. Materials and Methods

### 2.1. Occurrences of the Worst IIPs

**Figure 1:**
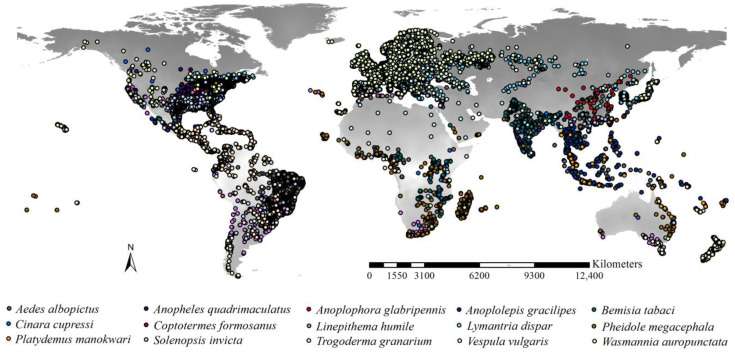
Occurrences of the 15 worst *IIPs*. Records were compiled from Global Biodiversity Information Facility, Global Invasive Species Database, and CABI Digital Library. We obtained 26,835 records after spatial rarefication.

The list of the 15 worst *IIPs* was compiled from the “100 of the World’s Worst Invasive Alien Species” created by the Invasive Species Specialist Group (ISSG) of the International Union for Conservation of Nature (IUCN) Species Survival Commission (www.iucngisd.org/gisd/100_worst.php, accessed on 2 February 2023) (Table 1). These 15 worst *IIPs* belong to 10 families, and Formicidae has five (ant) species, i.e., *Linepithema humile*, *Pheidole megacephala*, *Wasmannia auropunctata*, *Anoplolepis gracilipes*, and *Solenopsis invicta*. The data sources for the occurrences of the 15 worst *IIPs* were the following online datasets: Global Biodiversity Information Facility (www.gbif.org, accessed on 10 February 2023), Global Invasive Species Database (www.iucngisd.org, accessed on 10 February 2023), and Invasive Species Compendium by the CABI Digital Library (www.cabidigitallibrary.org, accessed on 10 February 2023). We compiled a preliminary occurrence dataset including 226,544 records. For each *IIP*, we used the method suggested by Zhou et al. (2024) [41] to remove occurrences with geographical co-ordinate uncertainty >5 km and spatially thinned the occurrences at an equal distance of 10 km. The final occurrence dataset contained 26,835 occurrence points, belonging to 15 sub-datasets of the worst *IIPs* (Figure 1). These data were used to project the range dynamics of the *IIPs*.

### 2.2. Predictors in the Range Dynamic Models

We compiled 31 predictors, including topographic (3), anthropogenic disturbance (9), and climate (19) factors, to predict the habitat suitability and ranges of the worst *IIPs*. A digital elevation model (DEM) downloaded from Worldclim (Fick and Hijmans, 2017) [42] was used to obtain the topographical predictors (i.e., elevation, slope, and aspect) with a 2.5 arc-min spatial resolution. Anthropogenic disturbance included 9 variables, i.e., population density and 8 land-use variables. Population density with a spatial resolution of 2.5 arc-min under current and future scenarios in 2100 was an interpolation dataset obtained from World Population Prospects (https://population.un.org/wpp/, accessed on 3 March 2023). The Land-Use Harmonization dataset (LUH2, https://luh.umd.edu/, accessed on 5 March 2023) was used. It included eight predictors at a spatial resolution of 0.25 arc degree representing the fractions of rangeland, urban land, cropland, non-forested primary land, non-forested secondary land, managed pasture, primary forested land, and secondary forested land. We compiled two sets of land-use variables to represent the future scenarios: the Shared Socio-Economic Pathway (SSP) 126 and SSP585 scenarios in 2100. The SSP scenarios are a set of climate scenarios in which the changes of global society, demographics, and economics are important inputs [43,44]. SSP126 is an optimistic scenario with 2.6 W/m^2^ by the year 2100 which was designed with the aim of simulating a development that is compatible with the 2 °C target [43,44]. SSP585 is a pessimistic scenario in which an additional radiative forcing of 8.5 W/m^2^ by the year 2100 could be allowed, representing the upper boundary of the range of the SSP scenarios [43,44]. Nineteen climatic predictors (eight for temperature and eleven for precipitation) had a 2.5 arc-min spatial resolution [41]. The monthly rainfall and temperature datasets with a spatial resolution of 2.5 arc-minutes from 1990–2020 were obtained from the Climate Research Division (CRU, www.crudata.uea.ac.uk, accessed on 6 March 2023). The *R* package 4.3.0 Biovarcs by Fick & Hijmans (2017) [42] was used to calibrate 19 climate predictors under current conditions [42]. They corresponded to those from the Worldclim database [41]. The 19 climate predictors for the future scenarios in 2100 under the least (SSP126) and most (SSP585) climate change scenarios were obtained from Worldclim [42]. We used the climatic variable datasets calibrated by the two most reliable and complementary GCMs: FIO-ESM-2-0 (F) and MPI-ESM1-2-HR (M) [45]. We compiled five sets of predictors under five scenarios, i.e., current, F126, F585, M126, and M585 scenarios, which indicated current scenarios, the most optimistic scenario calibrated by FIO-ESM-2-0, the most pessimistic scenario calibrated by FIO-ESM-2-0, the most optimistic scenario calibrated by MPI-ESM1-2-HR, and the most pessimistic scenario calibrated by MPI-ESM1-2-HR, respectively. All datasets had a spatial resolution of 2.5 arc-min or were resampled to this spatial resolution.

### 2.3. Predictor Selection

We established preliminary SDMs using Biomod2, an ensemble platform of SDMs, and used jackknifing to calculate the factors’ importance values (IVs) for each *IIP* individually (Supplementary Materials S1). SDMs utilize species occurrence data and environmental predictors to predict species distributions across space and time. We used |0.7| as the threshold [40]. Pearson correlation analyses were conducted to assess collinearity among the predictors for each of the 15 *IIPs*, separately (Supplementary Materials S2). Predictors with low IVs were eliminated if strong collinearity existed. We input the remaining predictors into 15 formal SDMs to predict the ranges and habitat suitability of the 15 *IIPs* individually.

### 2.4. Estimating Habitat Suitability and Potential Ranges

Biomod2, an ensembled platform of SDMs [46], was used to predict the habitat suitability and potential ranges of the *IIPs* and create raster maps. It is an *R* package 4.3.0 which incorporates a wide range of algorithms to produce combined projections, and, therefore, could treat a range of methodological uncertainties [46]. We parameterized species distribution models (SDMs) for each worst *IIP*, separately (Supplementary Materials S3). We used the following eleven algorithms: Surface Range Envelope, Xtreme Gradient Boosting (XGBoost), Multiple Adaptive Regression Splines, Random Forest, Maximum Entropy Model, Maxnet, Generalized Linear Model, Artificial Neural Network, Classification Tree, Generalized Boosting Model, and Flexible Discriminant Analysis [46]. As recommended by Thuiller et al. (2009) [46], the pseudo-absences (PAs) for each species were generated through surface range envelope technique. Then, 1000 PAs were obtained when the number of the worst *IIP* occurrences was less than 1000 [47]. Otherwise, the number of PAs matched the number of the worst *IIPs* occurrences [46]. First, we created habitat suitability maps of the worst *IIPs* under each scenario. Then, we used the maximum sum of sensitivity and specificity (MSS) to calibrate the *IIPs*’ ranges [48]. Five-fold cross-validation was used to assess the SDMs’ performances. We used 70% of the samples for SDM establishment and 30% for reliability assessment [49]. The algorithms with a true skill statistic (TSS) greater than 0.6 or an area under the curve (AUC) greater than 0.8 were incorporated into the ensemble SDMs [50], and then we obtained a list of algorithms included in SDMs for each species (Supplementary Materials S4). To obtain assembled rasters of SDM projection, a weight proportional to their TSS evaluation was given to each raster produced by algorithm’s projection. In total, we developed distribution maps for 15 species × 5 climate scenarios, totaling 75 distribution maps used to project habitat suitability and potential ranges of the 15 worst *IIPs*.

### 2.5. Dynamics in Habitat Suitability

The shifts in habitat suitability for the worst *IIPs* were obtained by differencing the habitat suitability raster maps (representing the occurrence probability of the worst *IIPs*) under future and current scenarios. We overlaid all habitat suitability raster maps of the worst *IIPs* to determine the total habitat suitability indices under different scenarios. The rasters of total habitat suitability shifts for all 15 *IIPs* were derived by differencing the total habitat suitability maps under future and current scenarios.

### 2.6. Estimating Range Dynamics

We used our recently developed dynamic models [39] to investigate the range dynamics of the worst *IIPs*. We categorized the total ranges into range expansions, range contractions, and stable ranges for each of the worst *IIPs* individually (the ranges shared by the current and future scenarios for the worst *IIPs* individually). Thus, the potential ranges under the current scenarios (*RCS*) were equal to the sum of the range contractions and stable ranges. The potential ranges under the future scenarios (*RFS*) were equal to the sum of stable ranges and range expansions. The range dynamics were estimated using the range ratio index (*RRI*) and the range similarity index (*RSI*). The *RRI* was used to estimate the shifts in the range sizes from current to future scenarios:

$$RRI=\frac{RFS}{{RCS}^{\prime }}$$

If *RRI* > 1, the *RFS* is larger than the *RCS*.

The *RSI* was used to assess the shifts in the position of the ranges from current to future scenarios:

$$RSI=\frac{2SR}{RCS+{RFS}^{\prime }}$$

where *SR* is the ranges shared by the current and future scenarios for the worst *IIPs* individually. If the *RSI* > 0.5, the *RCS* and *RFS* have similar centroids. Additionally, both higher range ratio index and lower range similarity index indicated larger range shifts.

We overlaid the raster maps of the 15 worst *IIP*^′^ ranges under current and future conditions separately to examine overall spatial patterns of their potential ranges through simple additive algorithm. In total, we obtained five maps of the overall spatial patterns of their potential ranges, i.e., one under current and four under future scenarios. We also overlaid the range expansions, range contractions, and stable ranges separately to assess their spatial patterns under the five scenarios.

## 3. Results

### 3.1. Model Performance

The AUCs of the SDMs of the 15 species ranged from 0.956 to 0.999, with an average of 0.989 ± 0.010. The TSSs were 0.823 to 0.992, with an average of 0.919 ± 0.046 (Supplementary Materials S5). The high AUC and TSS scores indicate the high robustness of the SDMs, ensuring the robustness of the predicted potential ranges. Specifically, the highest AUCs (0.99) occurred for *Linepithema humile*, *Anoplophora glabripennis*, *Anopheles quadrimaculatus*, *Vespula vulgaris*, *Anoplolepis gracilipes*, *Platydemus manokwari*, *Coptotermes formosanus*, *Wasmannia auropunctata*, and *Solenopsis invicta*, and the lowest AUCs were observed for *Cinara cupressi* (0.956) and *Trogoderma granarium* (0.971). The highest TSSs occurred in *Coptotermes formosanus* (0.992), *Anopheles quadrimaculatus* (0.961), *Solenopsis invicta* (0.961), and *Platydemus manokwari* (0.97), and the lowest ones were observed for *Cinara cupressi* (0.823) and *Trogoderma granarium* (0.861).

### 3.2. Importances of the Predictors

**Figure 2:**
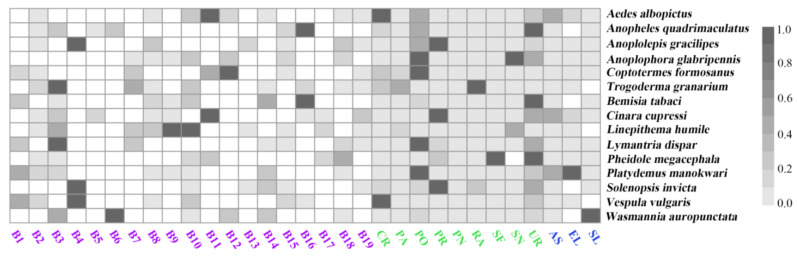
Importance of the predictors in the species distribution models. Note: B1, annual mean temperature; B2, mean diurnal range; B3, isothermality; B4, temperature seasonality; B5, max. temperature of warmest month; B6, min. temperature of coldest month; B7, temperature annual range; B8, mean temperature of wettest quarter; B9, mean temperature of driest quarter; B10, mean temperature of warmest quarter; B11, mean temperature of coldest quarter; B12, annual precipitation; B13, precipitation of wettest month; B14, precipitation of driest month; B15, precipitation seasonality; B16, precipitation of wettest quarter; B17, precipitation of driest quarter; B18, precipitation of warmest quarter; B19, precipitation of coldest quarter; CR, cropland; PA, managed pasture; PN, non-forested primary land; PO, population density; PR, forested primary land; RA, rangeland; SF, forested secondary land; SN, non-forested secondary land; UR: urban land; AS, aspect; EL, elevation; SL, slope. Climatic, human disturbance, and topographical predictors were in purple, green, and blue, respectively. The importance values of the predictors were min–max-standardized for each species, separately.

The relative importance of the predictors for the baseline distribution models depended on the species. For example, the top predictor in the SDM of the common wasp (*Vespula vulgaris*) was temperature seasonality, followed by annual mean temperature and the mean temperature in the warmest season with importance values of 0.165, 0.147, and 0.053, respectively. The top variables in the SDM of the common malaria mosquito (*Anopheles quadrimaculatus*) were population density (0.084), fraction of urban land (0.041), and mean temperature in the warmest season (0.020) (Figure 2, Supplementary Materials S6). The highest importance value (0.279) occurred for the fraction of urban land in the SDMs for *Coptotermes formosanus*, while the lowest value (0) was observed for aspect in the SDMs for most species. The climatic predictors had higher importance values than the topographical and anthropogenic disturbance predictors in the models for the nine worst *IIPs*, including *Lymantria dispar*, *Trogoderma granarium*, *Wasmannia auropunctata*, *Vespula vulgaris*, *Anoplolepis gracilipes*, *Cinara cupressi*, *Aedes albopictus*, *Pheidole megacephala*, and *Linepithema humile*, in which isothermality, temperature seasonality, min. temperature of the coldest month, and mean temperature of the coldest quarter showed the highest importance values (Figure 2, Supplementary Materials S6). The anthropogenic disturbance predictors had a higher importance than the climatic and topographical predictors in the models for the six worst *IIPs*, including *Anoplophora glabripennis*, *Anopheles quadrimaculatus*, *Platydemus manokwari*, *Coptotermes formosanus*, *Solenopsis Invicta*, and *Bemisia tabaci*, in which population density and the fractions of urban land played the strongest roles (Figure 2, Supplementary Materials S6). Additionally, the importance values of the topographical predictors were lower than those of the climate and anthropogenic disturbance predictors in the SDMs of all worst *IIPs* (Figure 2, Supplementary Materials S6).

### 3.3. Habitat Suitability of the Worst IIPs

The habitat suitability patterns of the 15 worst *IIPs* differed for different scenarios. For example, the Formosan subterranean termite (*Coptotermes formosanus*) was predicted to have a high habitat suitability in the southeastern United States under current conditions. In contrast, a high habitat suitability was predicted under the F585 scenario in the southeastern United States, southeastern China, Europe, Japan, the Korean peninsula, the Philippines, Malaysia, Indonesia, and Uganda (Supplementary Materials S7). Additionally, the habitat suitability patterns also depended on the species. For example, a high habitat suitability was observed for the common malaria mosquito (*Anopheles quadrimaculatus*) in the southeastern United States under the F126 scenario. The common wasp (*Vespula vulgaris*) had a high habitat suitability in Europe (Supplementary Materials S7). Additionally, the high habitat suitability of *Pheidole megacephala*, *Anoplolepis gracilipes*, *Bemisia tabaci*, *Platydemus manokwari*, and *Wasmannia auropunctata* was observed in tropical and subtropical regions. A high habitat suitability occurred in the United States of America for *Anopheles quadrimaculatus* and *Solenopsis invicta* and in Europe for *Vespula vulgaris*. The high habitat suitability of most of the worst *IIPs* was predicted for Europe, the United States of America, and tropical and subtropical regions.

**Figure 3:**
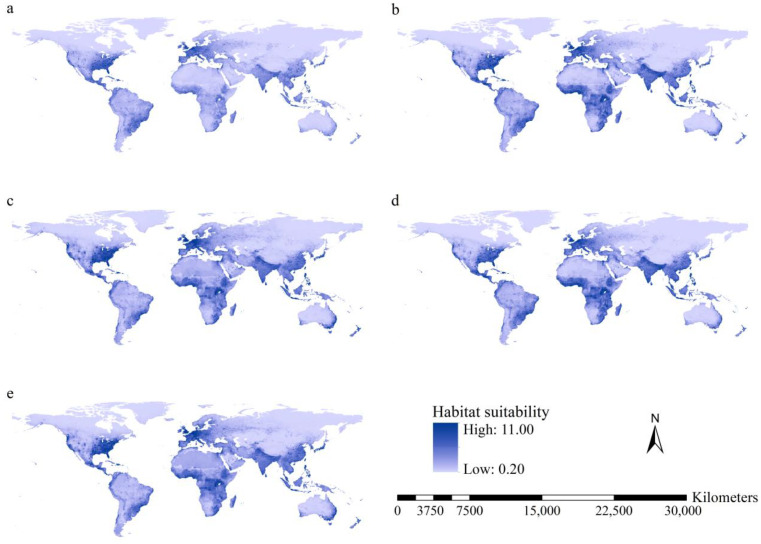
Total habitat suitability of the 15 worst *IIPs*: (**a**) current conditions; (**b**) F126 scenario; (**c**) F585 scenario; (**d**) M126 scenario; and (**e**) 585 scenario. High habitat suitability under all scenarios was observed on the west coast and in the southeastern United States of America, Mexico, the Caribbean Islands, the southeast coast of South America, Europe, South Asia, southeastern China, the Korean Peninsula, Japan, Indonesia, Philippines, east coast of Australia, Tasmania, and tropical regions of West Africa.

Higher total habitat suitability indices under current conditions occurred on the western coast and southeastern United States, Mexico, Caribbean Islands, southeastern coast of South America, Europe, South Asia, southeastern China, the Korean Peninsula, Japan, Indonesian, Philippines, and the eastern coast of Australia and Tasmania (Figure 3). The spatial patterns of the total habitat suitability indices were similar under current and future conditions. Higher total habitat suitability indices with larger areas were predicted for East Africa and the tropical regions of West Africa (Figure 3).

**Figure 4:**
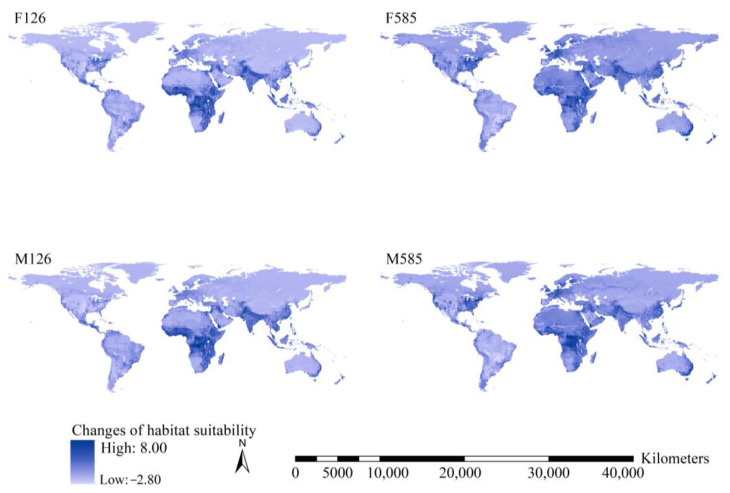
Changes in total habitat suitability of the 15 worst *IIPs*. Substantial increases of total habitat suitability indices under the F126 and M126 scenarios were projected for the southern slope of the Himalayan Mountains and tropical regions of Africa. Substantial increases of total habitat suitability indices under the F585 and M585 scenarios were projected for the west coast and the southeastern United States of America, tropical regions of Africa, Europe, the southern slope of the Himalayan Mountains, East China, Central Asia, West Asia, Japan, and southeast coast of Australia.

Under the F126 and M126 scenarios, substantial increases in total habitat suitability were projected for the southern slope of the Himalaya Mountains and tropical regions in Africa, ranging from 15° south latitude to 15° north latitude, except for South Sudan (Figure 4). Under the F585 and M585 scenarios, substantial increases in total habitat suitability indices were predicted for the western coast and southeastern United States, tropical regions of Africa, Europe, the southern slope of the Himalaya Mountains, East China, Central Asia, West Asia, Japan, and the southeastern coast of Australia (Figure 4).

Additionally, areas with increased total habitat suitability covered 114.9, 110.6, 123.5, and 120.9 million km^2^ under the F126, F585, M126, and M585 scenarios, respectively. Areas with decreased total habitat suitability covered 19.8, 23.0, 11.1, and 13.8 million km^2^. Therefore, increases in total habitat suitability of the 15 worst *IIPs* are predicted for more than 85% of the global terrestrial regions under future scenarios.

### 3.4. Potential Ranges of the Worst IIPs

The thresholds for determining the ranges depended on the species (Supplemental Material S8). For instance, the MSS thresholds under current conditions were 0.35 for the red imported fire ant (*Solenopsis invicta*) and 0.46 for the Argentine ant (*Linepithema humile*). The MSS thresholds for calibrating the potential ranges also depended on the scenario (Supplemental Material S8). For example, the MSS thresholds for the Flatworm (*Platydemus manokwari*) were 0.31, 0.60, 0.43, 0.59, and 0.62 under the current, F126, F585, M126, and M585 scenarios, respectively (Supplementary Materials S8). The highest thresholds were observed for *Bemisia tabaci* (0.66 under the F126 scenario), *Anoplolepis gracilipes* (0.64 under the F126 scenario), *Wasmannia auropunctata* (0.63 under the F126 scenario), and *Linepithema humile* (0.63 under the M126 scenario). The lowest ones occurred for *Cinara cupressi* (0.18 under the F585 scenario) and *Anoplophora glabripennis* (0.20 and 0.21 under the F585 and M585 scenarios, respectively).

The areas covered by the potential ranges of the 15 worst *IIPs* were 1.19 to 17.25, 2.14 to 22.00, 6.47 to 34.63, 1.45 to 25.21, and 3.59 to 41.04 million km^2^ under the current, F126, F585, M126, and M585 scenarios, respectively (Table 2). Under the current scenarios, the largest potential ranges occurred for *Cinara cupressi* (17.25 million km^2^), *Trogoderma granarium* (12.91 million km^2^), and *Aedes albopictus* (11.66 million km^2^). Under the F126 scenarios, *Aedes albopictus* (22.00 million km^2^), *Cinara cupressi* (19.83 million km^2^), and *Trogoderma granarium* (18.20 million km^2^) had the largest potential ranges. Under the F585 scenarios, *Cinara cupressi* (34.63 million km^2^), *Aedes albopictus* (27.11 million km^2^), and *Trogoderma granarium* (23.22 million km^2^) had the largest potential ranges. Under the M126 scenarios, the largest potential ranges occurred for *Trogoderma granarium* (25.21 million km^2^), *Cinara cupressi* (24.19 million km^2^), and *Aedes albopictus* (21.74 million km^2^). Under the M585 scenarios, *Trogoderma granarium* (41.04 million km^2^), *Cinara cupressi* (30.44 million km^2^), and *Aedes albopictus* (26.63 million km^2^) had the largest potential ranges. The Asian tiger mosquito (*Aedes albopictus*), cypress aphid (*Cinara cupressi*), Khapra beetle (*Trogoderma granarium*), and big-headed ant (*Pheidole megacephala*) occurred four, four, four, and three times in the top five largest potential ranges under the four future scenarios, respectively (Table 2). The red imported fire ant (*So. invicta*), Formosan subterranean termite (*C. formosanus*), flatworm (*Platydemus manokwari*), common malaria mosquito (*Anopheles quadrimaculatus*), and crazy ant (*Anoplolepis gracilipes*) occurred four, four, four, four, and three times in the top five smallest potential ranges under the four scenarios, respectively (Table 2). A paired-sample t-test showed that the range areas were smaller under the current than the future scenarios and under the F126 and M126 than under the F585 and M585 scenarios, respectively (*p* < 0.01).

Additionally, the spatial distributions of the potential ranges were species-specific (Supplementary Materials S9). For example, the current range of the Formosan subterranean termite (*Coptotermes formosanus*) includes the southeastern United States and tropical and subtropical regions in Asia and Japan, covering 1.19 million km^2^. The current range of the Khapra beetle (*Trogoderma granarium*) includes Europe, Africa, Central Asia, West Asia, and India, covering 12.90 million km^2^. The spatial patterns of the potential ranges differed for different scenarios (Supplementary Materials S9). For example, the potential future range of the flatworm (*Platydemus manokwari*) under the F126 scenario included the southeastern coast of the United States, Nepal, the southeastern coast of China, and tropical regions in Asia, covering 3.55 million km^2^ (Supplementary Materials S9). Its future range under the F585 scenario was the southeastern United States, southeastern China, Nepal, tropical regions in Africa and Asia, Brazil, and the southeastern coast of Australia, covering 8.32 million km^2^ (Supplementary Materials S9). In summary, the potential ranges of *Pheidole megacephala*, *Anoplolepis gracilipes*, *Bemisia tabaci*, *Platydemus manokwari*, and *Wasmannia auropunctata* were located primarily in tropical and subtropical regions. The potential ranges of *Anopheles quadrimaculatus* and *Solenopsis invicta* were observed primarily in the United States of America, whereas those of *Vespula vulgaris* were projected to occur in Europe.

**Figure 5:**
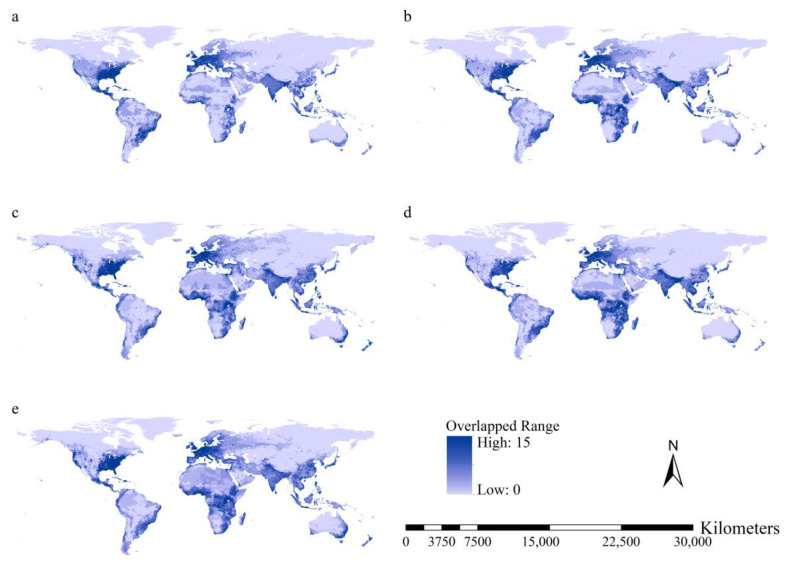
Overlapping potential ranges of the 15 worst *IIPs*: (**a**) overlapping ranges under current conditions; (**b**) overlapping potential ranges under the F126 scenario; (**c**) overlapping potential ranges under the F585 scenario; (**d**) overlapping potential ranges under the M126 scenario; and (**e**) overlapping potential ranges under the M585 scenario. Most overlaps occurred in west coast regions and the southeastern United States of America, Europe, Mexico, southeastern South America, tropical regions in Africa (except South Sudan), East Africa, tropical regions in Asia, southeastern China, Japan, and the Korean peninsula.

Although the geographical patterns of the range overlaps of the 15 worst *IIPs* depended on the scenario, most overlaps occurred on the west coast and southeastern part of the United States, Europe, Mexico, southeastern South America, tropical regions in Africa except South Sudan, East Africa, tropical regions in Asia, southeastern China, Japan, and the Korean peninsula (Figure 5).

### 3.5. Range Dynamics of the Worst IIPs

Most of the worst *IIPs* exhibited range expansions under all future scenarios except for a slight range contraction of the flatworm (*Platydemus manokwari*) under the F126 and M585 scenarios (Table 2). The average *RRIs* were 1.47, 1.56, 2.26, and 1.93 under the F126, M126, F585, and M585 scenarios, respectively. Under the F126 scenarios, *Anoplophora glabripennis* (2.32), *Aedes albopictus* (1.84), and *Coptotermes formosanus* (1.72) exhibited the largest *RRIs* (the largest range expansions). Under the F585 scenarios, *Coptotermes formosanus* (5.25), *Anoplophora glabripennis* (3.83), and *Pheidole megacephala* (2.39) had the largest *RRIs*. The largest *RRIs* under the M126 scenarios occurred for *Anoplophora glabripennis* (2.66), *Trogoderma granarium* (1.93), and *Pheidole megacephala* (1.86). Under the M585 scenarios, *Trogoderma granarium* (3.13), *Coptotermes formosanus* (2.92), and *Anoplophora glabripennis* (2.85) had the largest *RRIs*. Under the F126 scenarios, the lowest *RSIs* (the largest centroid shifts) were observed for *Anoplophora glabripennis* (0.492), *Anoplophora glabripennis* (0.558), and *Solenopsis invicta* (0.602). Under the F585 scenarios, *Coptotermes formosanus* (0.291), *Anoplophora glabripennis* (0.363), and *Solenopsis invicta* (0.470) had the lowest *RSIs*. Under the M126 scenarios, *Anoplophora glabripennis* (0.427), *Solenopsis invicta* (0.572), and *Platydemus manokwari* (0.579) had the lowest *RSIs.* The lowest *RSIs* under the M585 scenarios occurred for *Coptotermes formosanus* (0.391), *Trogoderma granarium* (0.472, and *Anoplophora glabripennis* (0.472). Most *RSIs* of the 15 worst *IIPs* under the four future scenarios exceeded 0.5 (Table 2). The average *RSIs* were 0.70, 0.67, 0.54, and 0.58 under the F126, M126, F585, and M585 scenarios, respectively (Table 2). Paired-sample t-tests showed that the *RRIs* and *RSIs* were lower and higher under the F126 and M126 than under the F585 and M585 scenarios, respectively (*p* < 0.01).

The range dynamics of the worst *IIPs* were species-specific (Table 2). For example, the *RRI* and *RSI* of the Asian long-horned beetle (*Anoplophora glabripennis*) under the M585 scenario were 2.85 and 0.47. Those of the cypress aphid (*Cinara cupressi*) were 1.74 and 0.61, respectively. Additionally, the range dynamics also depended on the scenarios (Table 2). For example, the *RRI* and *RSI* of the Argentine ant (*Linepithema humile*) were 1.20 and 0.78 under the M126 scenario and 1.47 and 0.63 under the F585 scenario, respectively. Additionally, the Asian long-horned beetle (*An. glabripennis*), Asian tiger mosquito (*Ae. albopictus*), and Formosan subterranean termite (*Coptotermes formosanus*) occurred four, four, and three times in the lists of the top five highest *RRIs* under the four future scenarios. The Argentine ant (*Li. humile*), common wasp (*Vespula vulgaris*), flatworm (*Platydemus manokwari*), and sweet potato whitefly (*Bemisia tabaci*) occurred four, three, three, and three times in the lists of the top five lowest *RRI* under the four future scenarios, respectively (Table 2). The common wasp (*Vespula vulgaris*), Argentine ant (*Li. humile*), sweet potato whitefly (*Bemisia tabaci*), and crazy ant (*Anoplolepis gracilipes*) were detected four, four, three, and three times in the lists of the top five highest *RSIs* under the four future scenarios (Table 2). The Asian long-horned beetle (*Anoplophora glabripennis*), flatworm (*Platydemus manokwari*), Formosan subterranean (*Co. formosanus*), and red imported fire ant (*So. invicta*) occurred four times in the lists of the top five lowest *RSIs* under the four future scenarios, respectively (Table 2).

**Figure 6:**
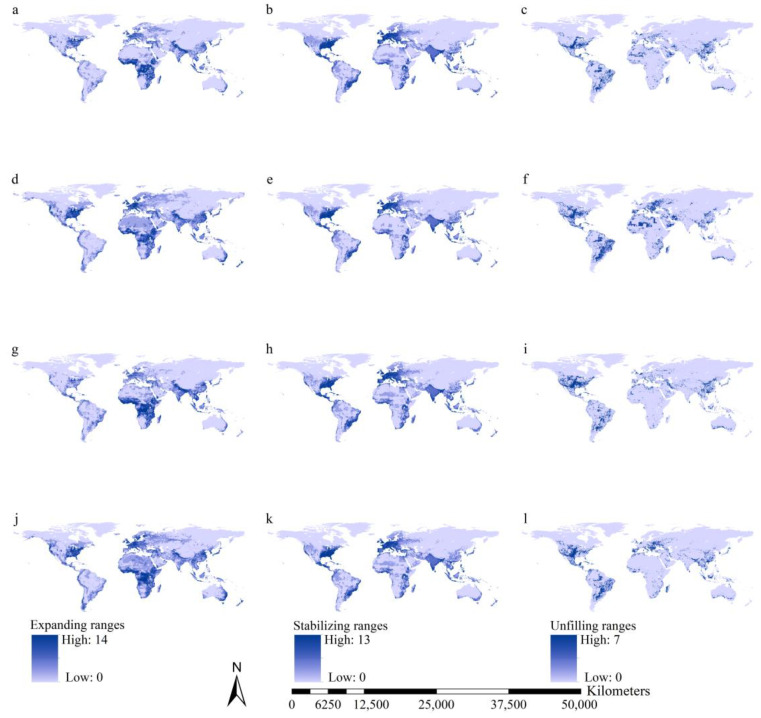
Overlapped expanding, stabilizing, and unfilling ranges of the 15 worst invasive insects: (**a**) expanding ranges under current-F126 scenarios; (**b**) stabilizing ranges under current-F126 scenarios; (**c**) unfilling ranges under current-F126 scenarios; (**d**) expanding ranges under current-F585 scenarios; (**e**) stabilizing ranges under current-F585 scenarios; (**f**) unfilling ranges under current-F585 scenarios; (**g**) expanding ranges under current-M126 scenarios; (**h**) stabilizing ranges under current-M126 scenarios; (**i**) unfilling ranges under current-M126 scenarios; (**j**) expanding ranges under current-M585 scenarios; (**k**) stabilizing ranges under current-M585 scenarios; and (**l**) unfilling ranges under current-M585 scenarios.

The range expansions of the 15 worst *IIPs* under the F126 and M126 scenarios overlapped in small regions in Western Europe, tropical regions in Africa except South Sudan, East Africa, a small region in the southeastern United States, Nepal, and tropical regions in Asia and East China (Figure 6). The range expansions under the F585 and M585 scenarios overlapped in Western Europe, the southeastern United States, tropical regions in Africa (except South Sudan), East Africa, Nepal, and tropical regions in Asia, East China, and Southeast Australia (Figure 6). Range contractions under the F126 and M126 scenarios occurred in scattered regions in the southern United States, East Africa, and tropical and subtropical regions in Asia. The range contractions were similar under the F585, M585, F126, and M126 scenarios, except in Africa (especially under F585). The stable ranges under all scenarios overlapped in the southeastern United States, Mexico, southeastern South America, Western Europe, scattered regions in Africa, and tropical and subtropical regions in Asia (Figure 6).

## 4. Discussion

A recently developed range dynamics model was used to predict the global range dynamics of the 15 worst *IIPs*. We detected the range expansions of most of the worst *IIPs*. Moreover, increases in the habitat suitability of the 15 worst *IIPs* were projected for ca. 85% of global terrestrial regions. Therefore, we might encounter an increased risk of the worst *IIPs* in the future, requiring stricter strategies to prevent or mitigate their spread. Additionally, our study applied a unified scheme to calibrate the range dynamics of the 15 worst *IIPs* to compare their invasion risks. We investigated, for the first time, the range expansions, range contractions, and stable ranges of the 15 worst *IIPs*. Therefore, our study provides novel insights into developing strategies to address future invasions of *IIPs*.

Numerous studies have shown that anthropogenic disturbance and climate change are dominant factors affecting the range dynamics of *IIPs* [7,13,15,18,22]. However, their relative influences on range dynamics were unclear. For instance, Liu et al. (2020) observed a stronger effect of anthropogenic disturbance than climate variation on the range variations of the fall armyworm (*Spodoptera frugiperda*) globally [25]. In contrast, Nie and Feng et al. (2023) predicted the opposite pattern in the range dynamics of *Aedes albopictus* [39]. The relative effect of anthropogenic disturbance and climate change on the range dynamics of insects depended on the scale, i.e., larger influences of climate change at larger scales and larger effects of anthropogenic disturbance at smaller scales [39]. Our global-scale study demonstrated that climate change had a larger effect than anthropogenic disturbance on the range dynamics of 60% of the worst *IIPs*, such as *Lymantria dispar*, *Trogoderma granarium*, and *Wasmannia auropunctata*. The opposite pattern was observed in 40% of the worst *IIPs*, such as *Anoplophora glabripennis*, *Anopheles quadrimaculatus*, and *Platydemus manokwari*. Although our study showed that temperature seasonality and the fraction of urban land contributed the most to range shifts, our findings imply that the dependence of these factors on the spatial scale was not universal, and their relative roles might be species-specific.

These observations also suggest that the relative influences of climate changes and anthropogenic disturbance on potential ranges of the worst *IIPs* depend on food availability and environmental tolerance. For example, *Anopheles quadrimaculatus* is a mosquito species that transmits malaria to humans [51]; therefore, its potential range was strongly affected by anthropogenic disturbance. However, the potential range of *Lymantria dispar* might be strongly affected by its climatic tolerance [52]. In summary, the relative effects of climate changes and anthropogenic disturbance on the potential ranges were species-specific and depended on species traits. However, uncertainty remains, and further investigations are required.

Our study showed that 30% of the 15 worst *IIPs* belong to Formicidae (i.e., *Linepithema humile*, *Pheidole megacephala*, *Wasmannia auropunctata*, *Anoplolepis gracilipes*, and *Solenopsis invicta*), while most other families only had 6.7 percentages (Table 1). Additionally, from the perspective of species richness, Formicidae is not the largest families among all families having *IIPs* [53]. It suggested that species belonging to Formicidae might have a stronger potential or higher probability of being the worst *IIPs* than those belonging to other families. Therefore, the invasive or introduced insects belonging to Formicidae should be paid much more attention than those belonging to other families.

Our study also indicated that the top three worst *IIPs* having the largest potential ranges under all of the five scenarios were *Cinara cupressi*, *Trogoderma granarium*, and *Aedes albopictus*, and all of them have been believed to mostly invade anthropogenic systems [54]. For example, *Trogoderma granarium* mostly invade grain barns around the world. It, to certain extent, implied that anthropogenic disturbance could facilitate the invasions of the worst *IIPs* [54], and the *IIPS* in anthropogenic systems might have a higher invasion risk than those in non-anthropogenic systems, though uncertainty might remain, and the influences of other factors, such as climate conditions, invasion history, and species traits, should not be neglected [10,54].

Topography may affect water and food availability, and rugged topography may be a barrier to the dispersion of *IIPs* [32,55,56,57,58]. However, our study indicated that topographical factors had less influence than climate change and anthropogenic disturbance on the range dynamics of the worst *IIPs*. Therefore, topographical factors might have negligible effects, whereas climate change and anthropogenic disturbance have dominant influences on the potential ranges of the worst *IIPs*. The weaker effects of topographical factors might be related to anthropogenic activities. In other words, transportation networks may facilitate the spread of *IIPs* since they are not affected by topographical barriers [25]. Therefore, strict strategies against *IIP* invasions are necessary, even in regions with rugged topography.

Our study showed that, relative to those under 126 scenarios, the worst *IIPs* under 585 scenarios were projected to experience more substantial range shifts (Table 2). In other words, most (pessimistic) change scenarios generally contributed the most to the range expansion and the centroid shifts in the worst *IIPs*, which was consistent with the findings by Nie and Feng (2023) [39]. *Anoplophora glabripennis*, *Coptotermes formosanu*, and *Trogoderma granarium* exhibited the largest range expansions and centroid shifts (Table 2) and had the fewest occurrence records (Supplementary Materials S3). This might be explained by Liu et al.’s (2020) hypothesis that species with small current distributions represent only a small part of their full invasion potentials [59]. Therefore, they have higher probabilities of large invasion potentials under future scenarios than species with large current distributions.

Our results showed that the Asian tiger mosquito (*Ae. albopictus*), cypress aphid (*Ci. cupressi*), Khapra beetle (*Tr. granarium*), and big-headed ant (*Ph. Megacephala*) exhibited larger range expansions than the other worst *IIPs* under most scenarios. Therefore, the focus should be on these species. Additionally, the Asian long-horned beetle (*An. glabripennis*), Asian tiger mosquito (*Ae. albopictus*), and Formosan subterranean termite (*Co. formosanus*) were predicted to have larger range expansions (indicated by higher *RRIs*) than the other worst *IIPs* under most future scenarios, suggesting stricter strategies will be required. The Asian long-horned beetle (*An. glabripennis*), flatworm (*Pl. manokwari*), Formosan subterranean termite (*Co. formosanus*), and red imported fire ant (*So. invicta*) exhibited larger centroid shifts than other species (indicated by lower *RSIs*) under most future scenarios. This result suggests the need for substantial strategy modifications in priority regions to prevent their invasions.

Under most future scenarios, range expansions and increases in habitat suitability occurred predominantly in tropical regions in Africa except South Sudan, Western Europe, and the southeastern United States, suggesting stricter strategies against the invasions of the 15 worst *IIPs* should be implemented in these areas. Additionally, range contractions were mostly predicted in scattered regions of the southern United States, southern East Africa, and Eastern Europe. This finding suggests that these areas are not priority regions for future strategies, and the focus should be on other regions.

The range dynamics of the 15 worst *IIPs* depended on future climate scenarios, and our investigation could not incorporate all future scenarios, representing one limitation of the SDMs. The scenarios, i.e., the optimistic, pessimistic, and other scenarios, and the GCMs affected future range dynamics. Therefore, the future range dynamics of the 15 worst *IIPs* remain uncertain, requiring further investigations.

## 5. Conclusions

We used a unified scheme to predict the range dynamics of the 15 worst *IIPs*. To the best of our knowledge, this is the first study investigating the range dynamics of the worst *IIPs* using a unified scheme. Although the range dynamics were species-specific, most species exhibited range expansions. The habitat suitability of these species was predicted to increase in more than 85% of global land, suggesting increasing invasion threats in the future. Therefore, more effective strategies are required to prevent or mitigate species invasions. Strategies for their detection and management should be species-specific. This study improves our understanding of how habitat changes may affect the ecology of the worst *IIPs* and shifts in their invasive potential.

## Figures and Tables

**Table 1:** The 15 world’s worst invasive insect pests in the study.

Common Names	Scientific Names	Family	Native Regions	Major Impacts
Argentine ant	*Linepithema humile*	Formicidae	South America	Biodiversity losses
Asian gypsy moth	*Lymantria dispar*	Erebidae	Asia and Europe	Forest losses
Asian longhorned beetle	*Anoplophora glabripennis*	Cerambycidae	Asia	Forest losses
Asian tiger mosquito	*Aedes albopictus*	Culicidae	Southeast Asia	Transmission of diseases
Big-headed ant	*Pheidole megacephala*	Formicidae	Southern Africa	Biodiversity and crop loss
Cocoa tree-ant	*Wasmannia auropunctata*	Formicidae	South America	Biodiversity losses
Common malaria mosquito	*Anopheles quadrimaculatus*	Culicidae	North America	Transmission of diseases
Common wasp	*Vespula vulgaris*	Vespidae	Europe	Human health and insect losses
Cotton whitefly	*Bemisia tabaci*	Aleyrodidae	India	Crop losses
Crazy ant	*Anoplolepis gracilipes*	Formicidae	Unclear	Biodiversity losses
Cypress aphid	*Cinara cupressi*	Aphididae	North America	Forest losses
Flatworm	*Platydemus manokwari*	Rhynchodemidae	New Guinea, Africa	Biodiversity losses
Formosa termite	*Coptotermes formosanus*	Rhinotermitidae	China	Economic losses
Khapra beetle	*Trogoderma granarium*	Dermestidae	Indian subcontinent	Food storage
Red imported fire ant	*Solenopsis invicta*	Formicidae	South America	Biodiversity losses

Note: The information of the 15 world’s worst invasive insect pests was compiled from the “100 of the World’s Worst Invasive Alien Species” created by the Invasive Species Specialist Group (ISSG) of IUCN Species Survival Commission (www.iucngisd.org/gisd/100_worst.php, 2 February 2023) and CABI Digital Library (www.cabidigitallibrary.org/, 3 February 2023).

**Table 2:** Potential ranges and range dynamics of the 15 worst invasive insect pests.

	Potential Ranges (million km^2^)					Range Ratio Index				Range Similarity Index			
Species	CurPR	F126PR	F585PR	M126PR	M585PR	F126RRI	F585RRI	M126RRI	M585RRI	F126RSI	F585RSI	M126RSI	M585RSI
*Linepithema humile*	7.16	8.76	10.92	8.87	10.41	1.19	1.47	1.20	1.40	0.79	0.63	0.78	0.66
*Anoplophora glabripennis*	4.23	9.74	16.11	11.20	12.00	2.32	3.83	2.66	2.85	** 0.49 **	** 0.36 **	** 0.43 **	** 0.47 **
*Aedes albopictus*	11.66	22.00	27.11	21.74	26.63	1.84	2.26	1.82	2.23	0.66	0.55	0.67	0.57
*Pheidole megacephala*	8.23	13.46	20.51	15.78	18.09	1.58	2.39	1.86	2.12	0.71	0.52	0.64	0.54
*Anopheles quadrimaculatus*	3.98	6.72	7.20	5.85	6.53	1.64	1.77	1.42	1.60	0.65	0.64	0.66	0.67
*Vespula vulgaris*	7.09	10.46	11.42	8.24	9.93	1.41	1.51	1.11	1.33	0.78	0.62	0.86	0.71
*Anoplolepis gracilipes*	5.84	8.12	9.57	9.01	9.14	1.41	1.67	1.57	1.59	0.75	0.69	0.73	0.69
*Cinara cupressi*	17.25	19.83	34.63	24.19	30.44	1.13	1.98	1.38	1.74	0.80	0.53	0.66	0.61
*Platydemus manokwari*	3.86	3.56	8.33	4.52	3.62	0.95	2.22	1.20	0.96	0.60	0.48	0.58	0.50
*Coptotermes formosanus*	1.19	2.14	6.47	1.45	3.59	1.72	5.25	1.17	2.92	0.56	** 0.29 **	0.58	** 0.39 **
*Lymantria dispar*	8.76	11.81	21.23	11.26	18.77	1.36	2.44	1.29	2.16	0.81	0.52	0.83	0.58
*Trogoderma granarium*	12.91	18.20	23.22	25.21	41.04	1.39	1.76	1.93	3.13	0.71	0.54	0.65	0.47
*Wasmannia auropunctata*	8.86	12.00	18.58	13.68	18.11	1.32	2.05	1.51	2.00	0.74	0.58	0.72	0.60
*Solenopsis invicta*	4.78	8.15	10.17	7.63	7.40	1.66	2.07	1.55	1.50	0.60	** 0.47 **	0.57	0.52
*Bemisia tabaci*	10.45	11.90	12.57	17.41	14.29	1.14	1.21	1.67	1.37	0.78	0.75	0.71	0.75

Note: CurPR, potential range under current scenarios; F126PR, potential range under F126 scenarios; F585PR, potential range under F585 scenarios; M126PR, potential range under M126 scenarios; M585PR, potential range under M585 scenarios; F126RRI, range ratio index under F126 scenarios; F585RRI, range ratio index under F585 scenarios; M126RRI, range ratio index under M126 scenarios; M585RRI, range ratio index under M585 scenarios; F126RSI, range similarity index under F126 scenarios; F585RSI, range similarity index under F585 scenarios; M126RSI, range similarity index under M126 scenarios; and M585RSI, range similarity index under M585 scenarios. The yellow-highlighted items indicated the top five largest potential ranges, top five largest range expansions, and top five largest shifts of range positions. The grey-highlighted items indicated range contraction. The bold fonts with underlines indicated ranges under current and future scenarios occupied different ns.

## Data Availability

All data have been supplied as Supplementary Materials.

## Supplementary Materials

The following supporting information can be downloaded at: https://www.mdpi.com/article/10.3390/insects15040280/s1, S1: Importance scores of all factors in the preliminary models; S2: Correlation analyses among all predictors; S3: Methods for parameterization in the models; S4: Algorithms incorporated in the formal species distribution models; S5: The AUCs and TSSs in the models for the ranges of the 15 worst *IIPs*; S6: Importance scores of the predictors in the formal models; S7: Habitat suitability maps of the 15 worst invasive insect pests; S8: Thresholds for calibrating the ranges of the 15 worst invasive insect pests; S9: Potential ranges of the 15 worst invasive insect pests.
