# Supplementary material for: Future Climate Change and Anthropogenic Disturbance Promote the Invasions of the World’s Worst Invasive Insect Pests
Source: Insects. 2024 Apr 16;15(4):280. doi: 10.3390/insects15040280 (PMC11050065; doi:10.3390/insects15040280)
Supplement: Supplementary file 1 [file insects-15-00280-s001.zip › Supplemental material S3.pdf]

S3 Methods for parameterization in the models

We used importance values (through jackknifing method) and multi-collinearity diagnosis (through Pearson correlation) to parameterize important predictors in the formal species distribution models for each species individually. For example, for *Platydemus manokwari*, its important predictors in our species distribution models included UR, B18, b11, PR, B17, B10, EL, PO, B2, SF, PA, PN, CR, SL, SN, AS and RA, while for *Anoplophora glabripennis*, they were UR, PO, B3, CR, B6, CR, B15, B5, B2, RA, B16, SL, PR, SF, SN, AS, PN, PA and RA. We used true skill statistic (TSS) and area under the curve (AUC) to parameterize algorithms which were not suitable predict potential range of each species individually. Therefore, different species might have different algorithms to project their potential ranges. For example, for *Anoplophora glabripennis*, the included algorithms were CTA, FDA, GBM, GLM, MARS, MAXENT, RF and XGBOOST while for *Linepithema humile,* they were ANN, CTA, FDA, GBM, GLM, MARS, MAXENT, RF and XGBOOST. The amounts of pseudo-absences (PAs) were parameterized through the amounts of species occurrence records for each species individually, i.e., 1000 PAs if the occurrence records being less than 1000, or equal amounts of PAs being randomly generated. For example, for *Linepithema humile*, *Anoplophora glabripennis*, *Aedes albopictus* and *Pheidole megacephala*, they had 1247, 1000, 8009 and 681 PAs, respectively. To parameterize assembled projection of each species individually, we used weights equaling to TSS of the species distribution models derived by each algorithm. For example, for *Anoplophora glabripennis*, projection by CTA was given the lowest weights, while for *Vespula vulgaris*, ANN was given the lowest weight. We also used the maximum sum of sensitivity and specificity (MSS) threshold to parameterize the potential ranges of each species. The key parameterizations in species distribution models for each species were listed in following table1.

Table S1 Key parameterizations in species distribution models (SDMs) for each species

Scientific Names	Amounts of occurrences	Predictors in SDMs	The number of PAs	Algorithms in SDMs	Thresholds for current scenarios	Thresholds for F126	Thresholds for F585	Thresholds for M126	Thresholds for M585 scenario
Aedes albopictus	8009	B11, PO, UN, B10, B12, EL, PA, PR, B14, CR, SL, RA, B2, B8, B19, SN, B18, PN, SF, AS	8009	CTA, FDA, GBM, GLM, MARS, MAXENT, MAXNET, RF, XGBOOST	0.53	0.56	0.42	0.55	0.43
Anopheles quadrimaculatus	569	UN, PO, B3, B6, CR, B15, B5, B2, RA, B16, SL, PR, SF, SN, AS, PN, PA	1000	CTA, FDA, GBM, GLM, MARS, MAXENT, MAXNET, RF, XGBOOST	0.405	0.26	0.32	0.25	0.27
Anoplolepis gracilipes	660	B4, PO, B18, B8, B13, UN, B2, RA, SL, PR, PA, EL, B15, CR, B19, PN, SF, AS, SN	1000	ANN, CTA, FDA, GBM, GLM, MARS, MAXENT, MAXNET, RF, XGBOOST	0.54	0.64	0.46	0.57	0.61
Anoplophora glabripennis	87	PO, UN, B10, B12, B15, B18, B7, PA, EL, SN, B9, PR, RA, PN, CR, B8, SF, SL, AS	1000	CTA, FDA, GBM, GLM, MARS, MAXENT, MAXNET, RF, XGBOOST	0.45	0.28	0.2	0.25	0.21
Bemisia tabaci	634	UN, B14, B1, B10, PO, B8, B3, B9, RA, B16, CR, PR, PA, SN, SL, PN, AS, SF	1000	CTA, FDA, GBM, GLM, MARS, MAXENT, MAXNET, RF, XGBOOST	0.34	0.51	0.43	0.56	0.5
Cinara cupressi	65	B11, UN, B19, B3, B5, EL, PO, B16, B8, PR, RA, CR, SN, B2, B9, B17, SL, PN, PA, AS, SF	1000	ANN, CTA, FDA, GBM, GLM, MARS, MAXENT, MAXNET, RF, XGBOOST	0.38	0.44	0.38	0.42	0.42
Coptotermes formosanus	95	PO, B11, CR, B7, B1, PA, RA, UN, B15, B12, SN, B2, PN, EL, B19, PR, SL, AS, SF	1000	CTA, FDA, GBM, GLM, MARS, MAXENT, MAXNET, RF, XGBOOST	0.48	0.66	0.54	0.5	0.57
Linepithema humile	1247	B10, B3, B8, B7, B17, PA, UN, B19, B2, B9, RA, PO, EL, CR, PR, B13, B15, SF, PN, SN, AS	1247	ANN, CTA, FDA, GBM, GLM, MARS, MAXENT, MAXNET, RF, XGBOOST	0.32	0.35	0.18	0.35	0.26
Lymantria dispar	6555	B3, UN, B1, B7, PR, B18, CR, EL, PN, PO, B14, SN, SL, RA, B19, B15, PA, SF, AS	6555	ANN, CTA, FDA, GBM, GLM, MARS, MAXENT, MAXNET, RF, XGBOOST	0.46	0.58	0.44	0.63	0.44
Pheidole megacephala	683	UN, B18, B11, PR, B17, B10, EL, PO, B2, SF, PA, PN, CR, SL, B3, AS, RA	1000	CTA, FDA, GBM, GLM, MARS, MAXENT, MAXNET, RF, XGBOOST	0.53	0.45	0.24	0.45	0.24
Platydemus manokwari	214	PO, B1, UN, B14, B3, B2, B10, B18, SN, EL, B9, PA, CR, SL, PR, PN, RA, SF, B8, AS	1000	ANN, CTA, FDA, GBM, GLM, MARS, MAXENT, MAXNET, RF, XGBOOST	0.53	0.58	0.38	0.55	0.47
Solenopsis invicta	2095	B4, UN, RA, B14, CR, PO, B15, PN, SN, PR, PA, B13, B10, SL, SF, B19, AS	2095	ANN, CTA, FDA, GBM, GLM, MARS, MAXENT, MAXNET, RF, XGBOOST	0.31	0.6	0.43	0.59	0.62
Trogoderma granarium	71	B3, B7, B10, CR, B16, B2, PO, PN, B18, RA, PR, B8, EL, UN, AS, SF, B14, SN, B19, PA	1000	CTA, FDA, GBM, GLM, MARS, MAXENT, MAXNET, RF, XGBOOST	0.35	0.35	0.29	0.38	0.39
Vespula vulgaris	5166	B4, B1, B10, UN, B15, B2, B8, EL, SF, CR, PO, PR, SN, RA, B16, PN, PA, AS	5166	ANN, CTA, FDA, GBM, GLM, MARS, MAXENT, MAXNET, RF, XGBOOST	0.49	0.42	0.3	0.51	0.34
Wasmannia auropunctata	685	B6, B3, B14, B12, UN, PO, B10, PA, B18, SL, PR, RA, CR, SF, PN, SN, AS	1000	CTA, FDA, GBM, GLM, MARS, MAXENT, MAXNET, RF, XGBOOST	0.55	0.63	0.38	0.6	0.37

Note: B1, Annual Mean Temperature; B2, Mean Diurnal Range; B3, Isothermality; B4, Temperature Seasonality; B5, Max Temperature of Warmest Month; B6, Min Temperature of Coldest Month; B7, Temperature Annual Range; B8, Mean Temperature of Wettest Quarter; B9, Mean Temperature of Driest Quarter; B10, Mean Temperature of Warmest Quarter; B11, Mean Temperature of Coldest Quarter; B12, Annual Precipitation; B13, Precipitation of Wettest Month; B14, Precipitation of Driest Month; B15, Precipitation Seasonality; B16, Precipitation of Wettest Quarter; B17, Precipitation of Driest Quarter; B18, Precipitation of Warmest Quarter; B19, Precipitation of Coldest Quarter; CR, cropland; PA, managed pasture; PN, non-forested primary land; PO, Population density; PR, forested primary land; RA, rangeland; SF, forested secondary land; SN, non-forested secondary land; UR: urban land; AS, aspect; EL, elevation; SL, slope. XGBOOST, Xtreme Gradient Boosting; MARS, Multiple Adaptive Regression Splines; RF, Random Forest; MAXENT, Maximum Entropy Model; GLM, Generalized Linear Model; ANN, Artificial Neural Network; CT, Classification Tree; GBM, Generalized Boosting Model; FDA, Flexible Discriminant Analysis; MAXNET, Maxnet model.
