# Supplementary material for: Future Climate Change and Anthropogenic Disturbance Promote the Invasions of the World’s Worst Invasive Insect Pests
Source: Insects. 2024 Apr 16;15(4):280. doi: 10.3390/insects15040280 (PMC11050065; doi:10.3390/insects15040280)

S7 Maps of habitat suitability of 15 worst insect pest

*Linepithema humile* Current, F126, F585, M126 and M585 in this order


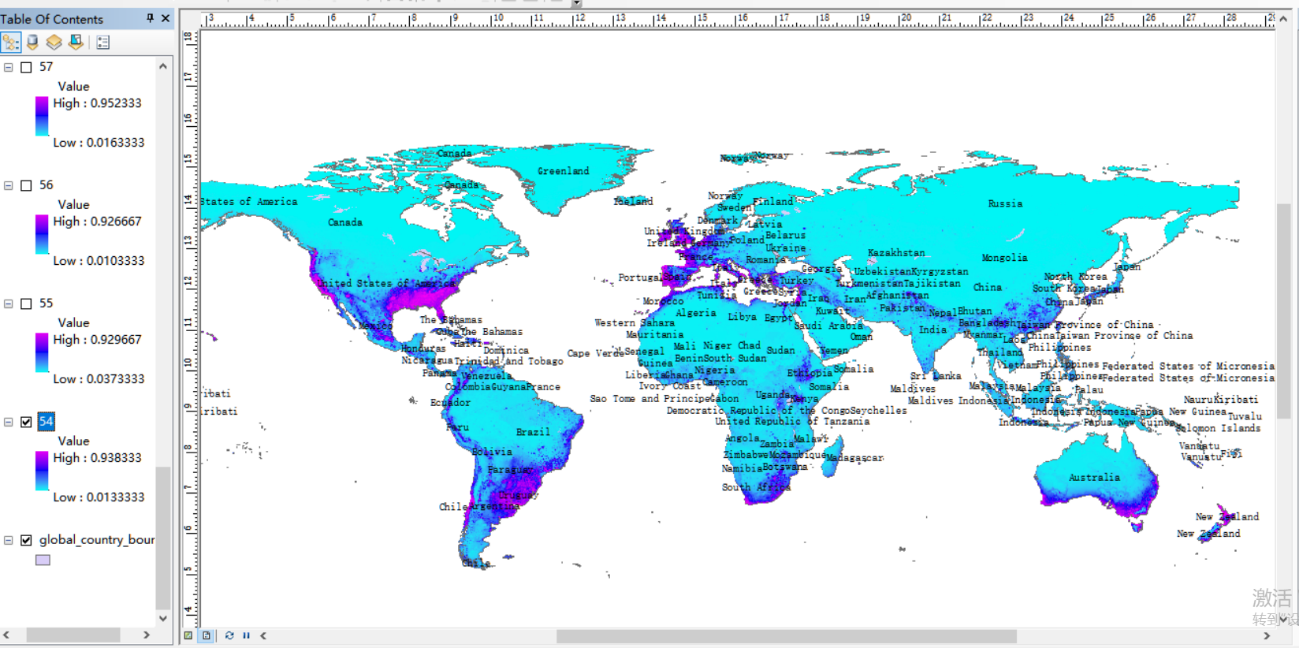


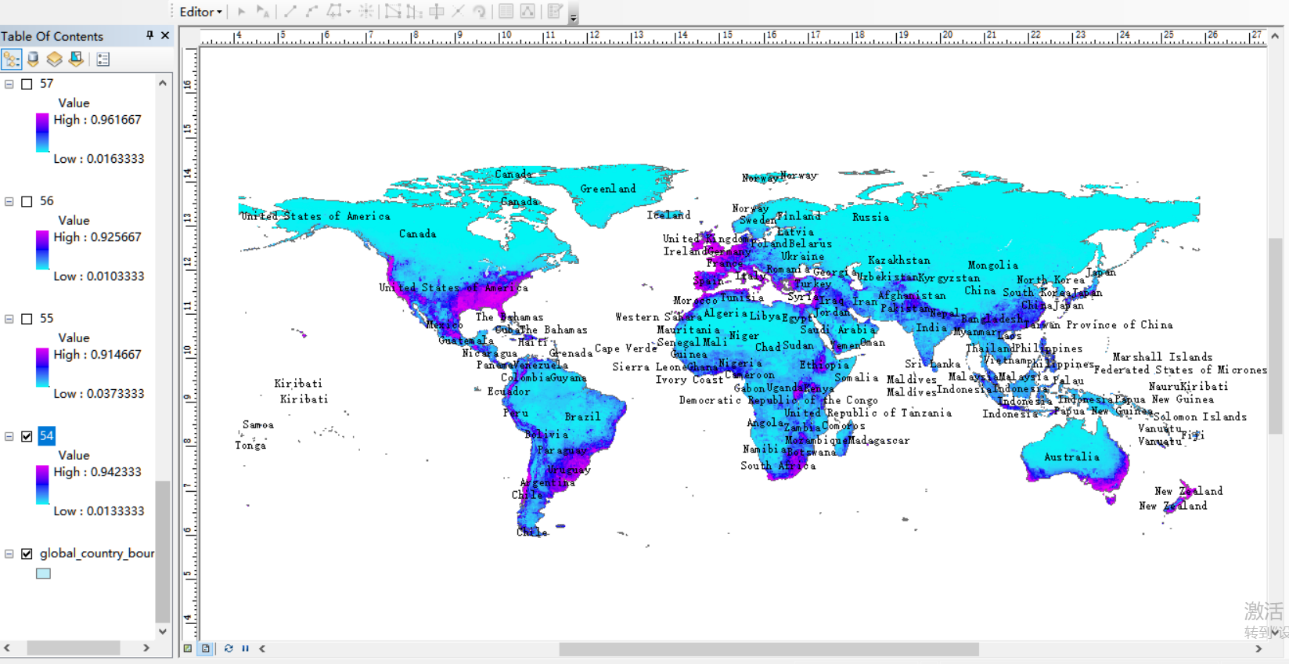


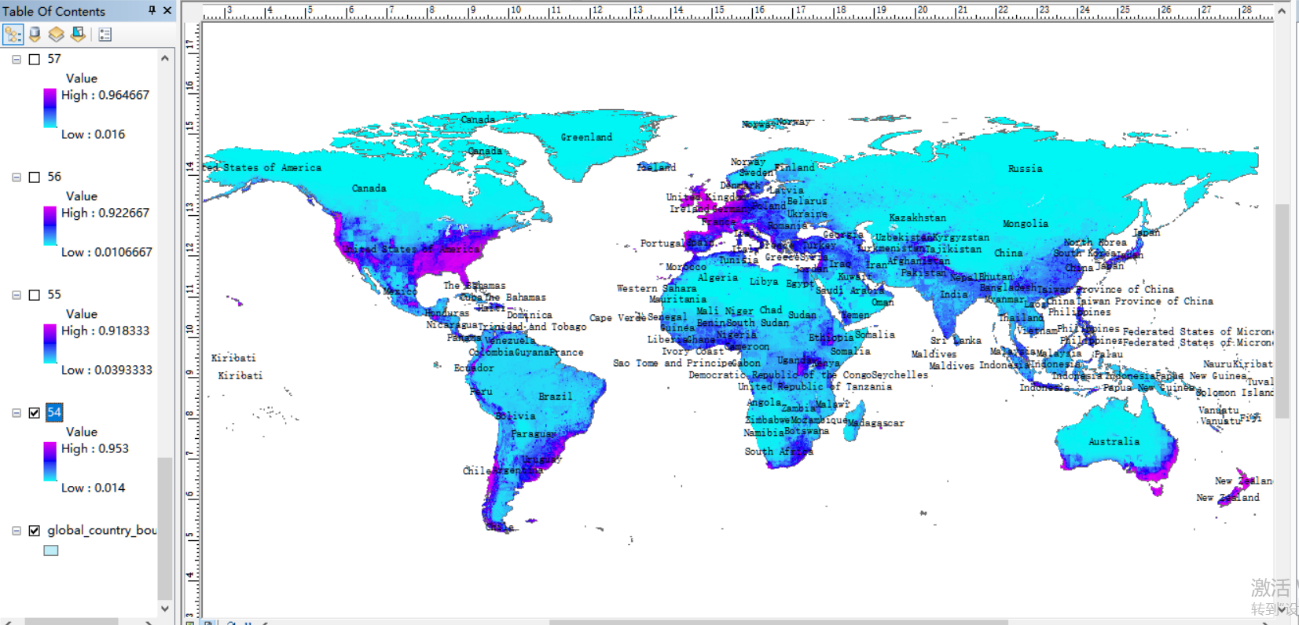


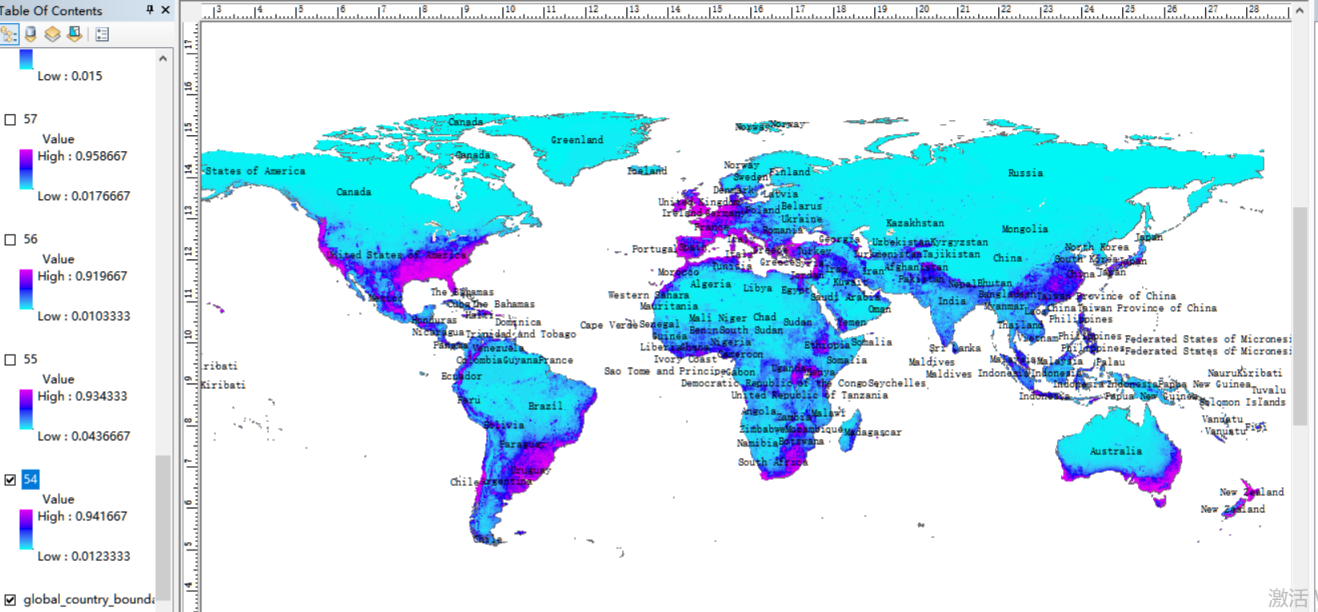


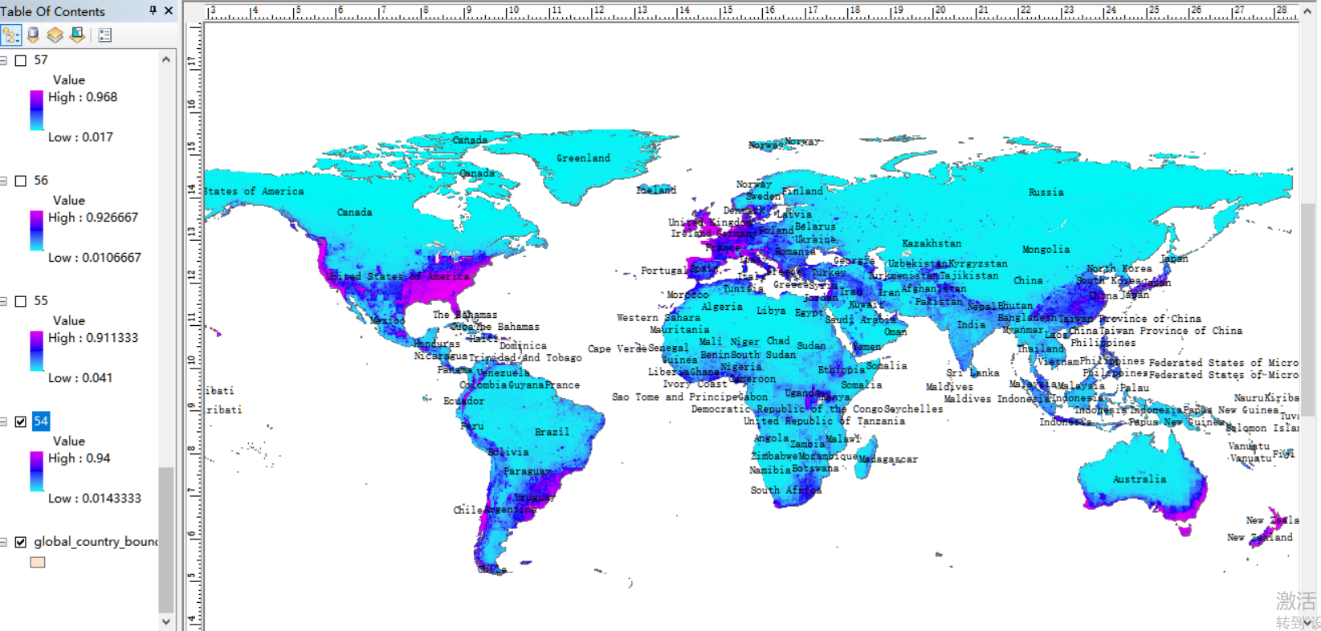


*Anoplophora glabripennis*  Current, F126, F585, M126 and M585 in this order


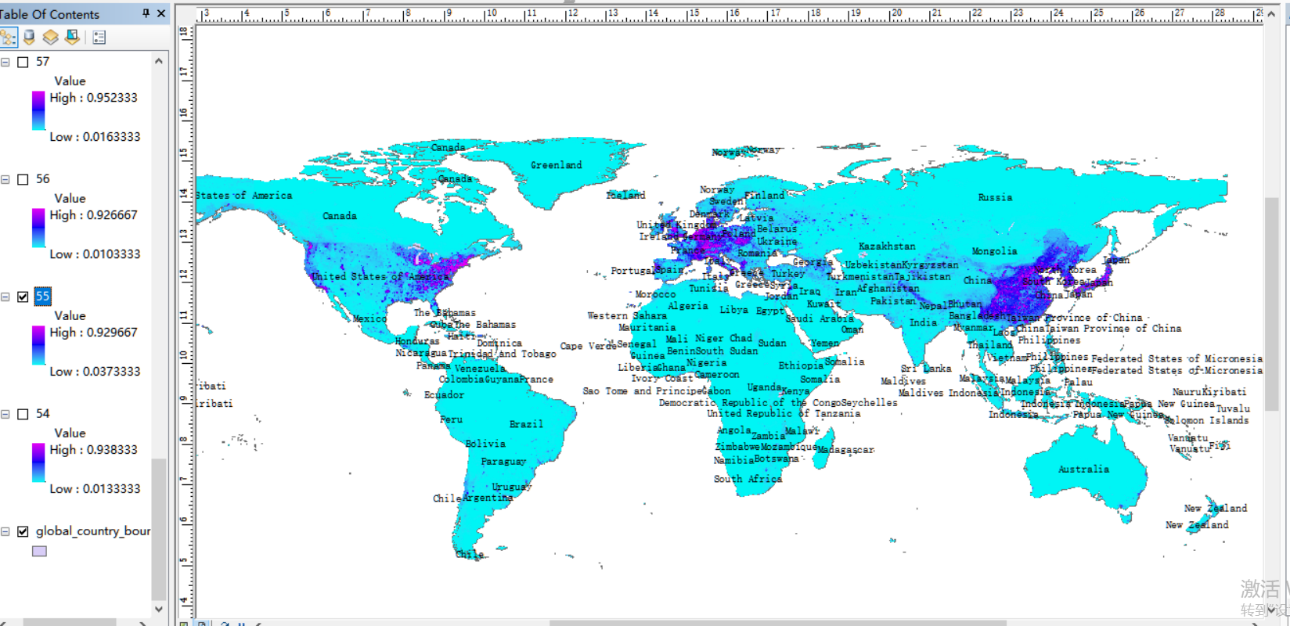


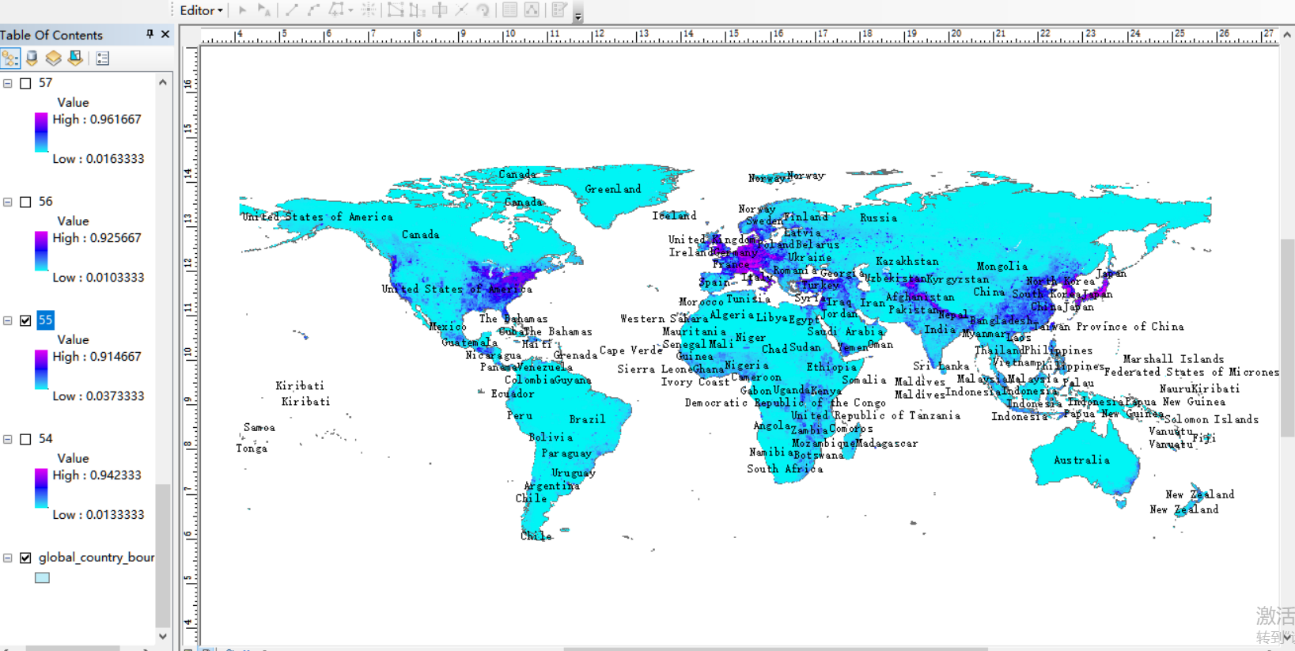


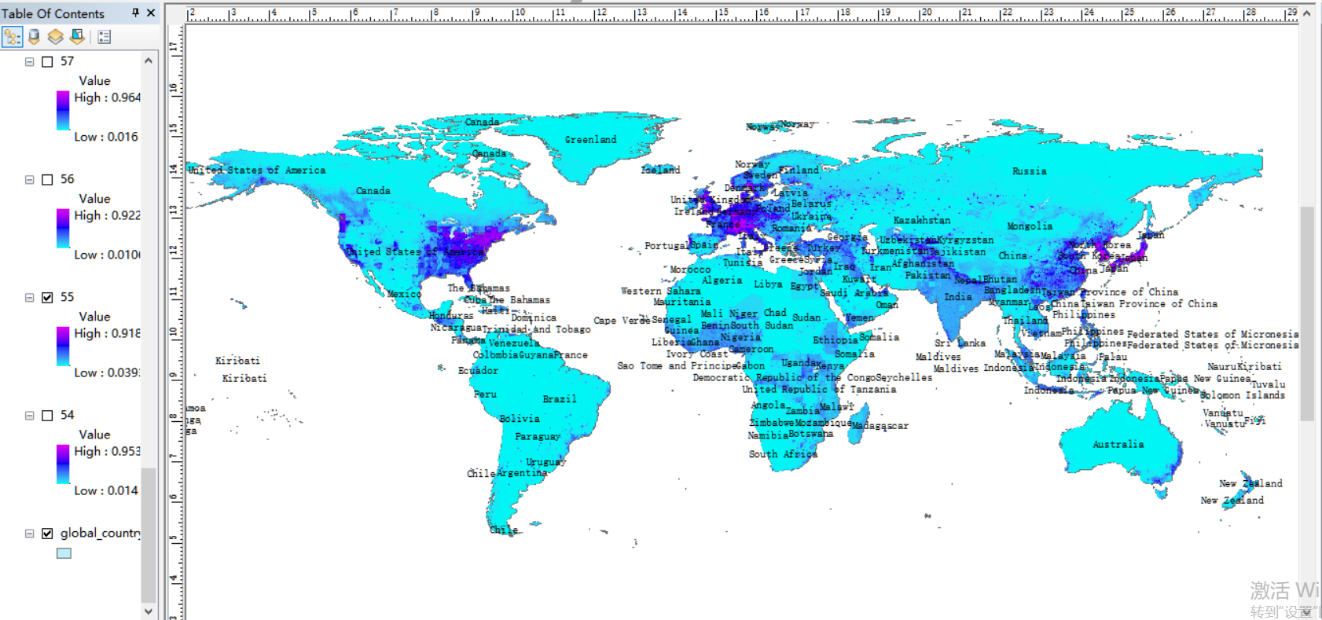


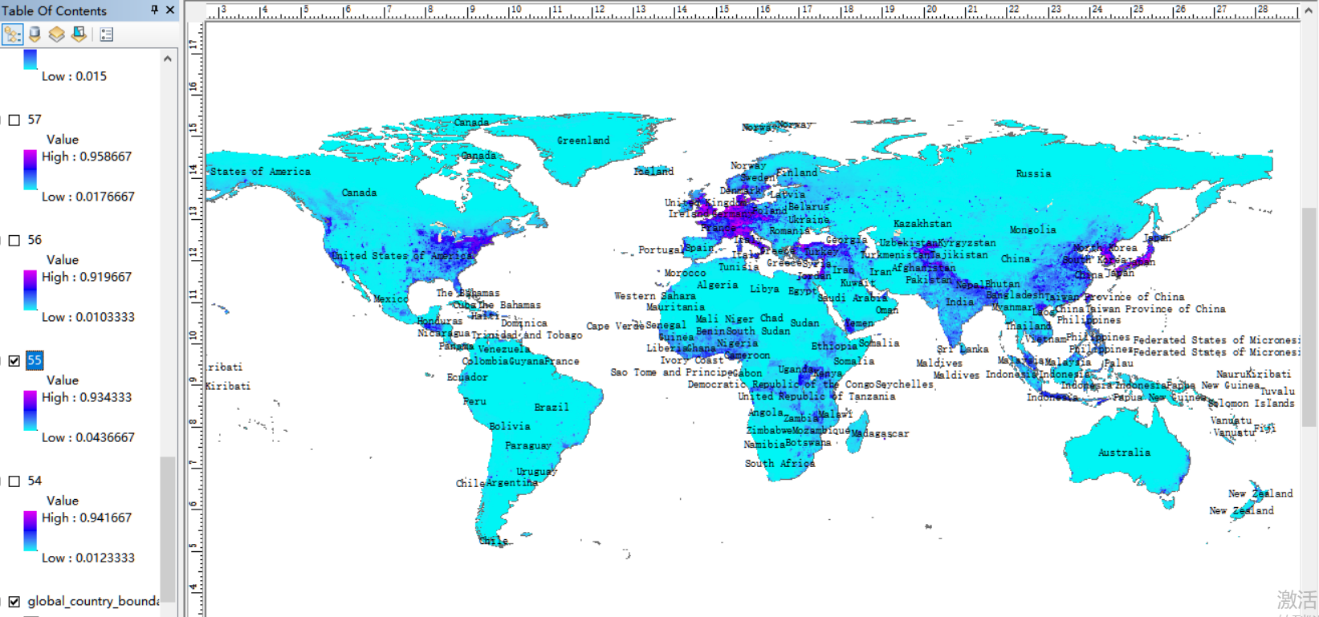


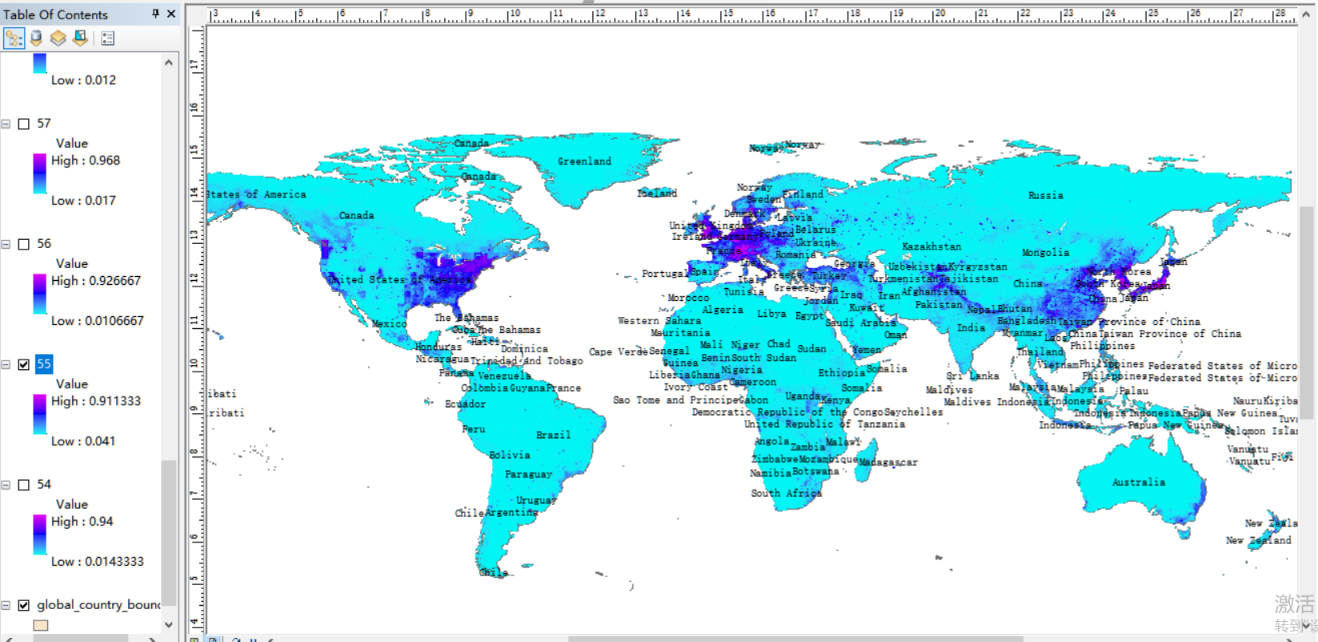


*Aedes albopictus*  Current, F126, F585, M126 and M585 in this order


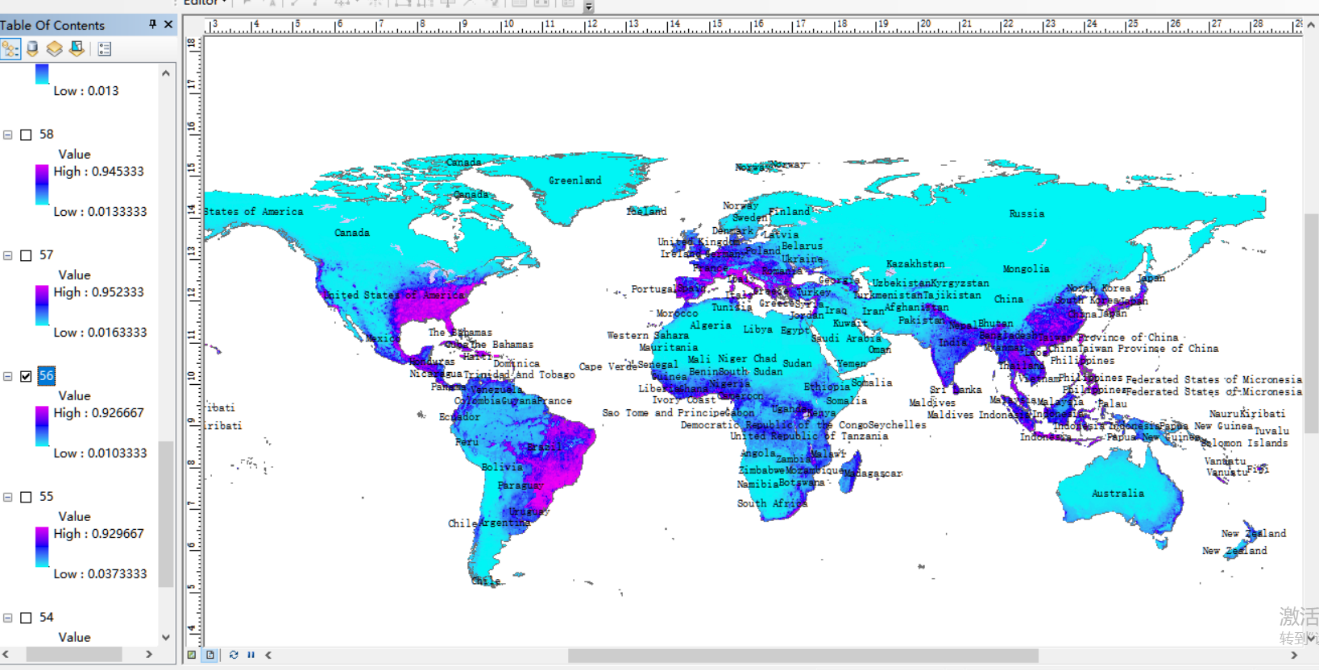


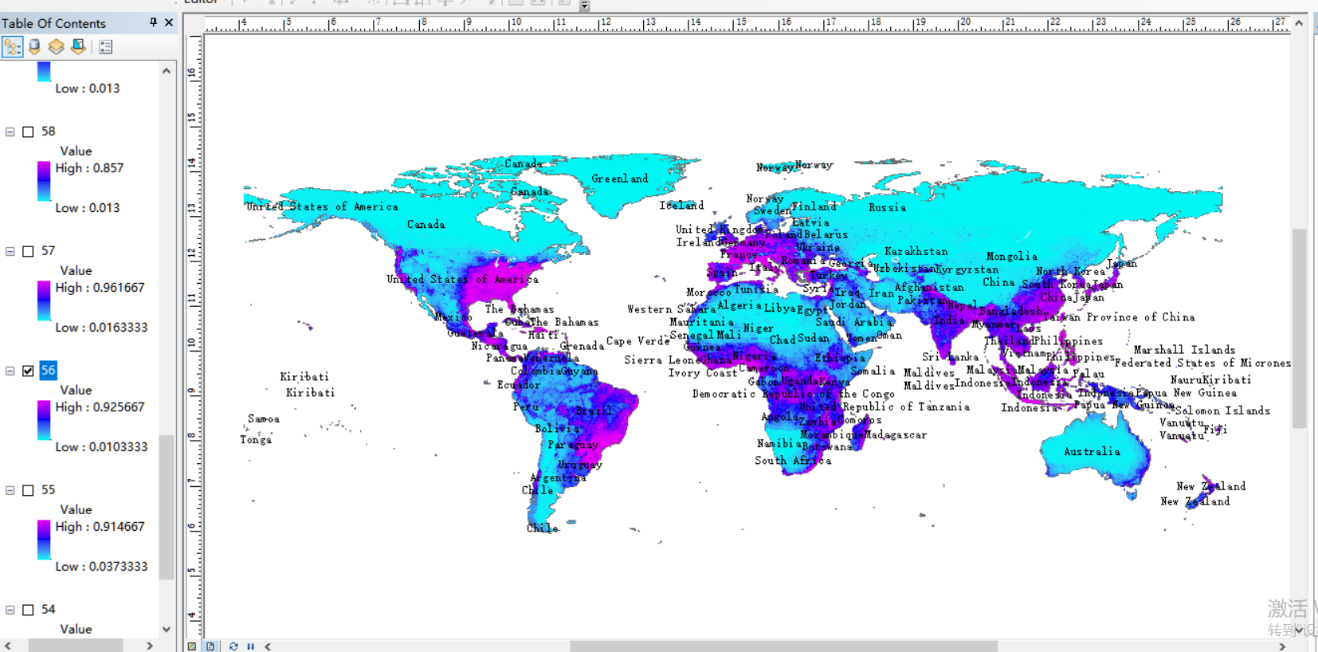


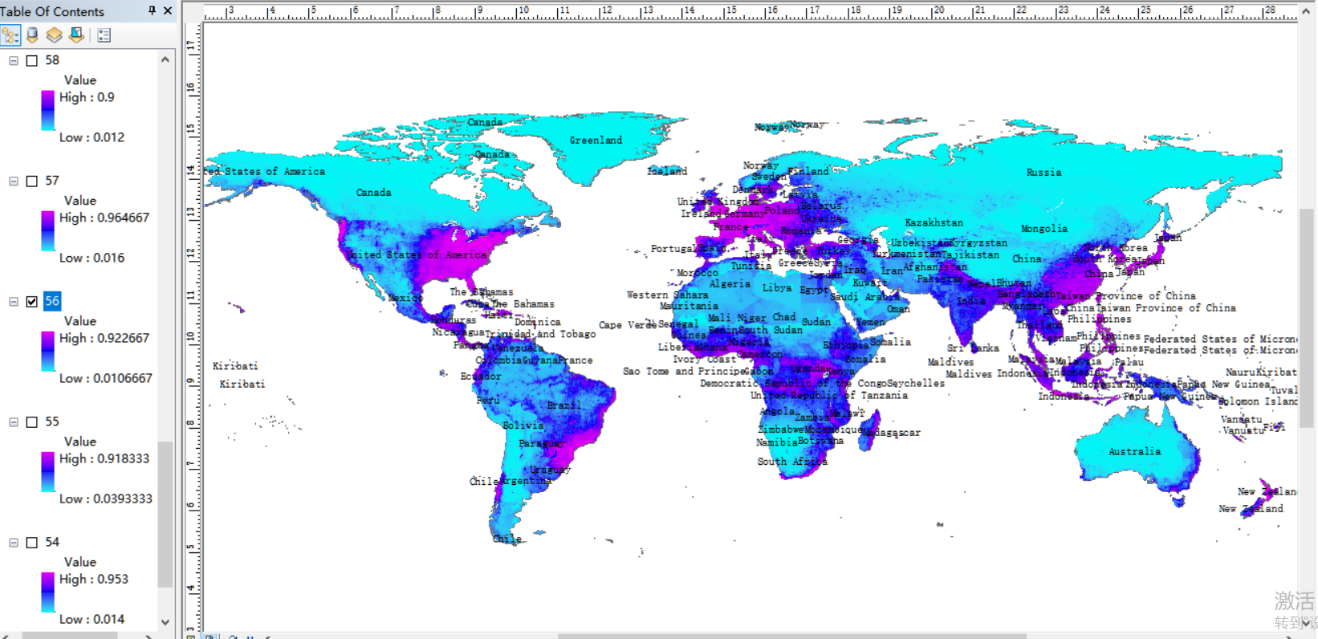


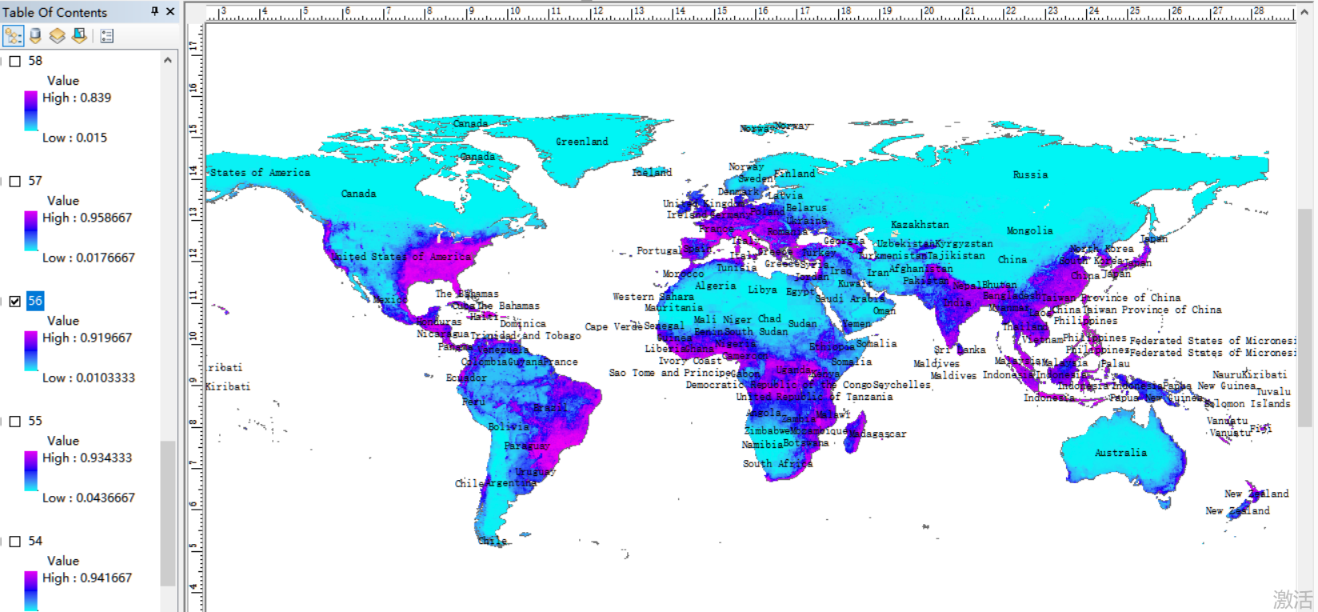


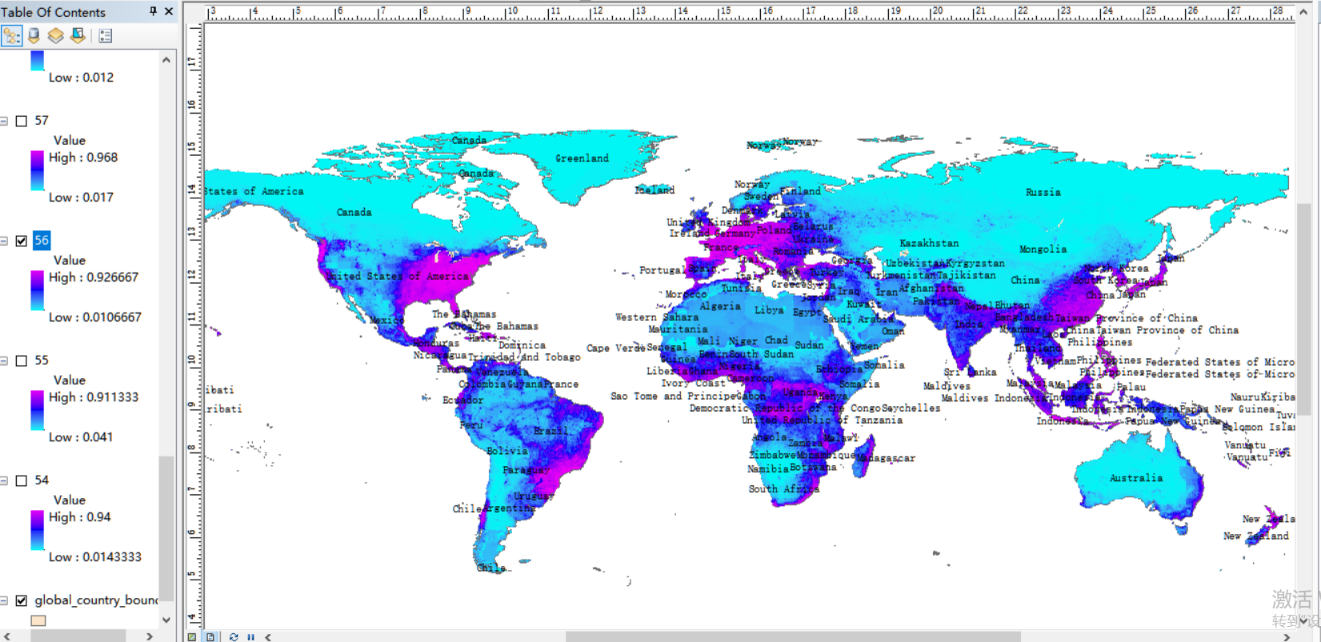


*Pheidole megacephala* Current, F126, F585, M126 and M585 in this order


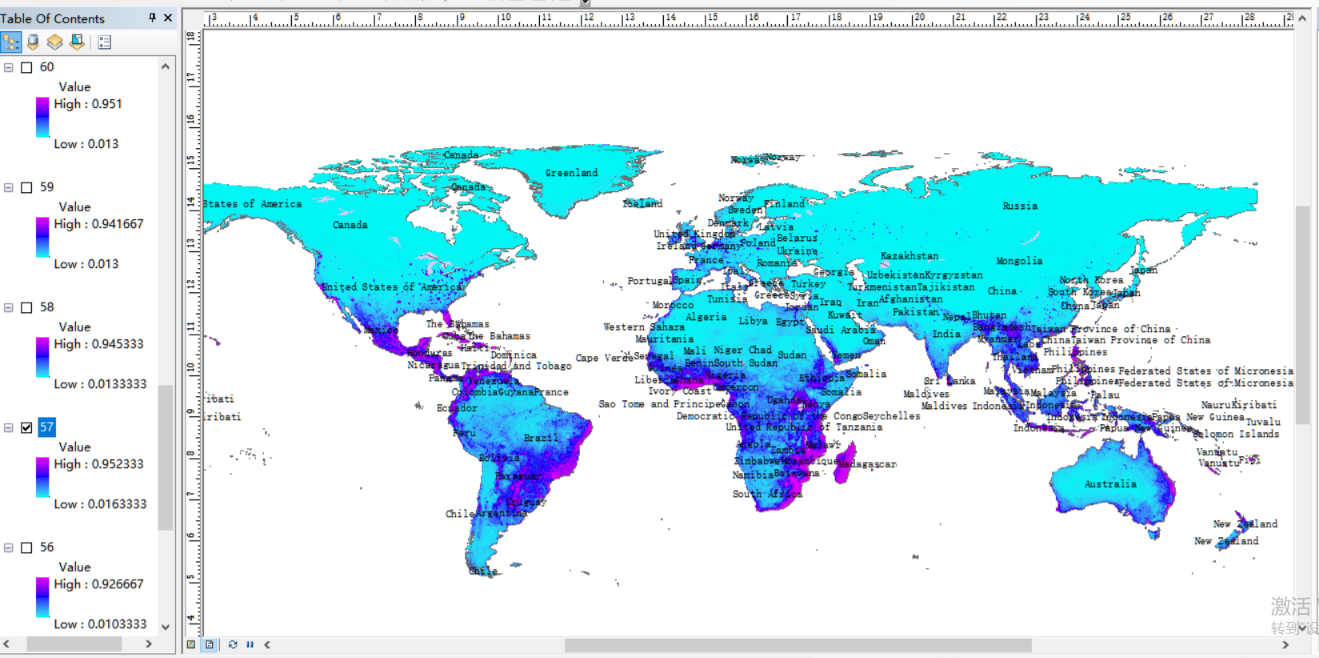


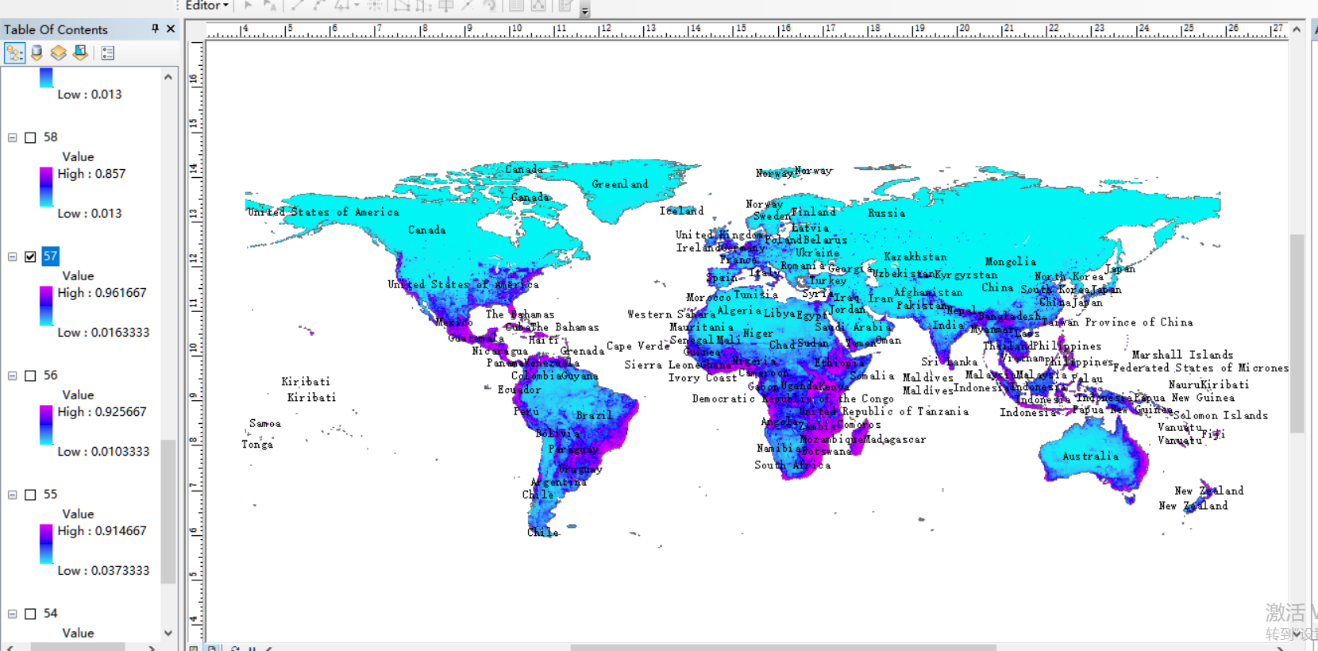


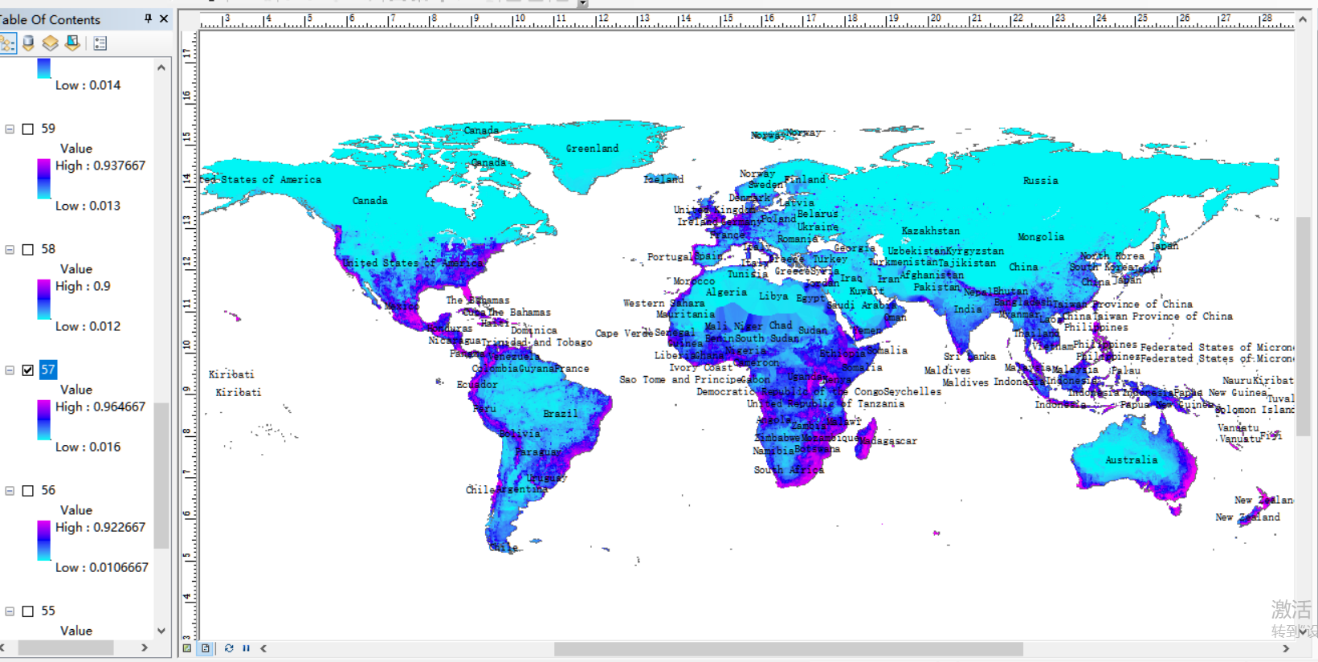


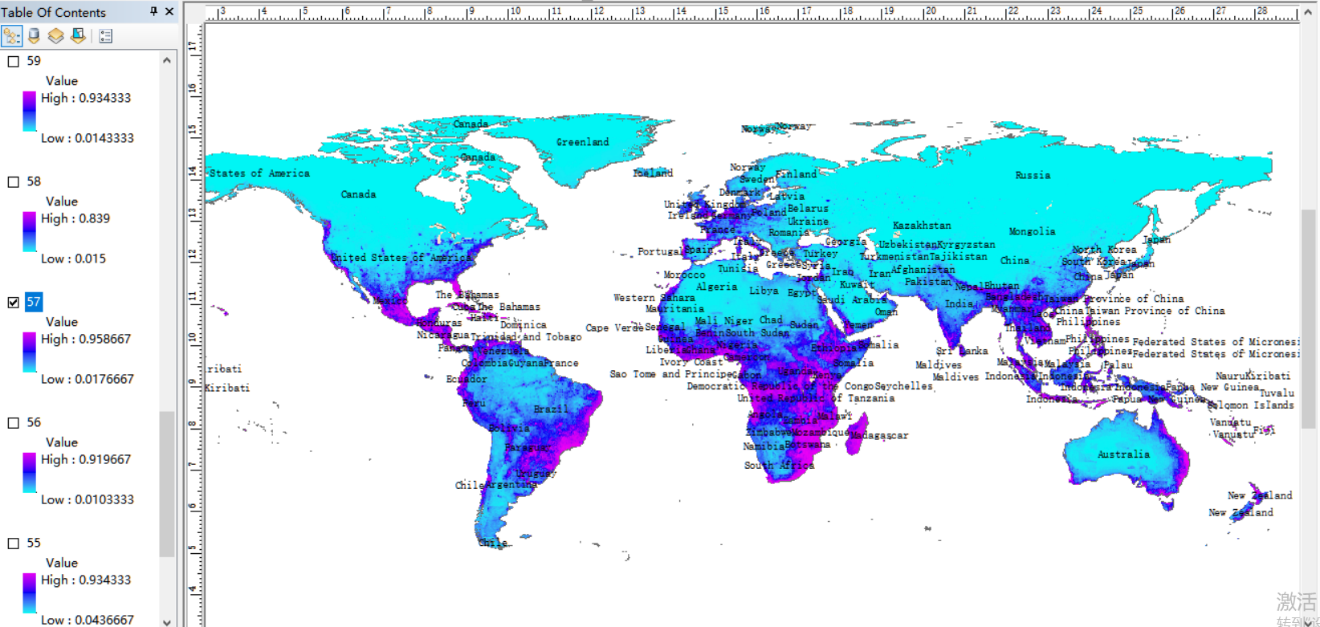


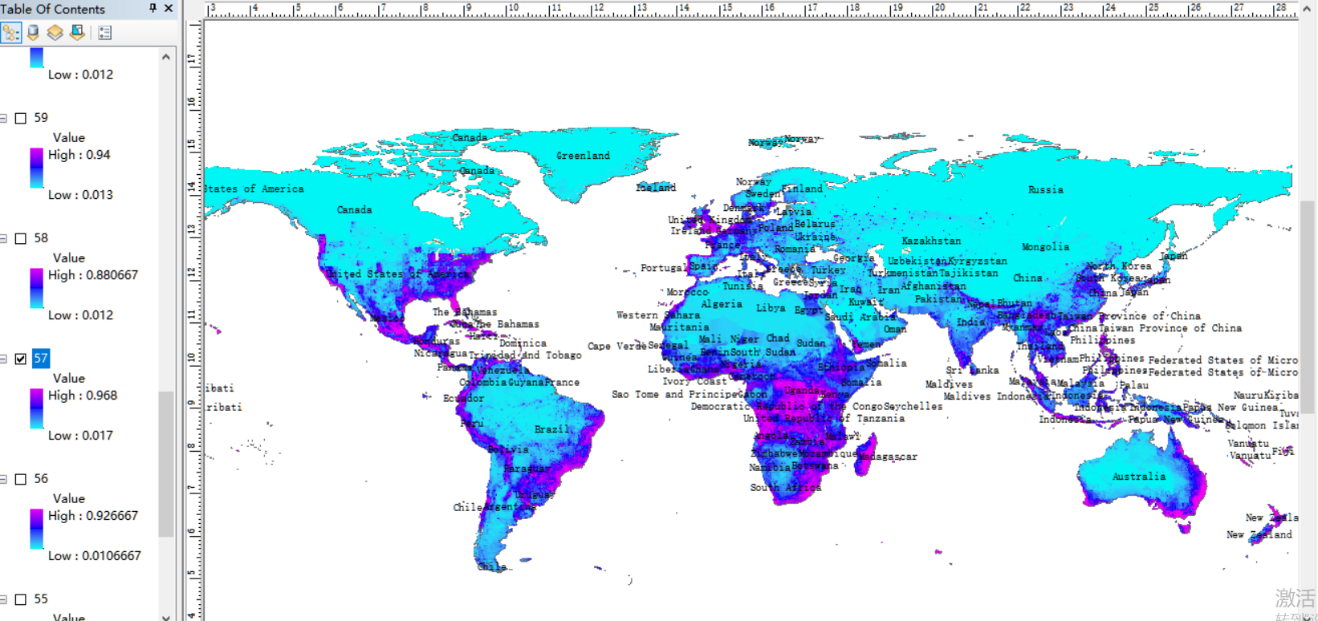


*Anopheles quadrimaculatus*  Current, F126, F585, M126 and M585 in this order


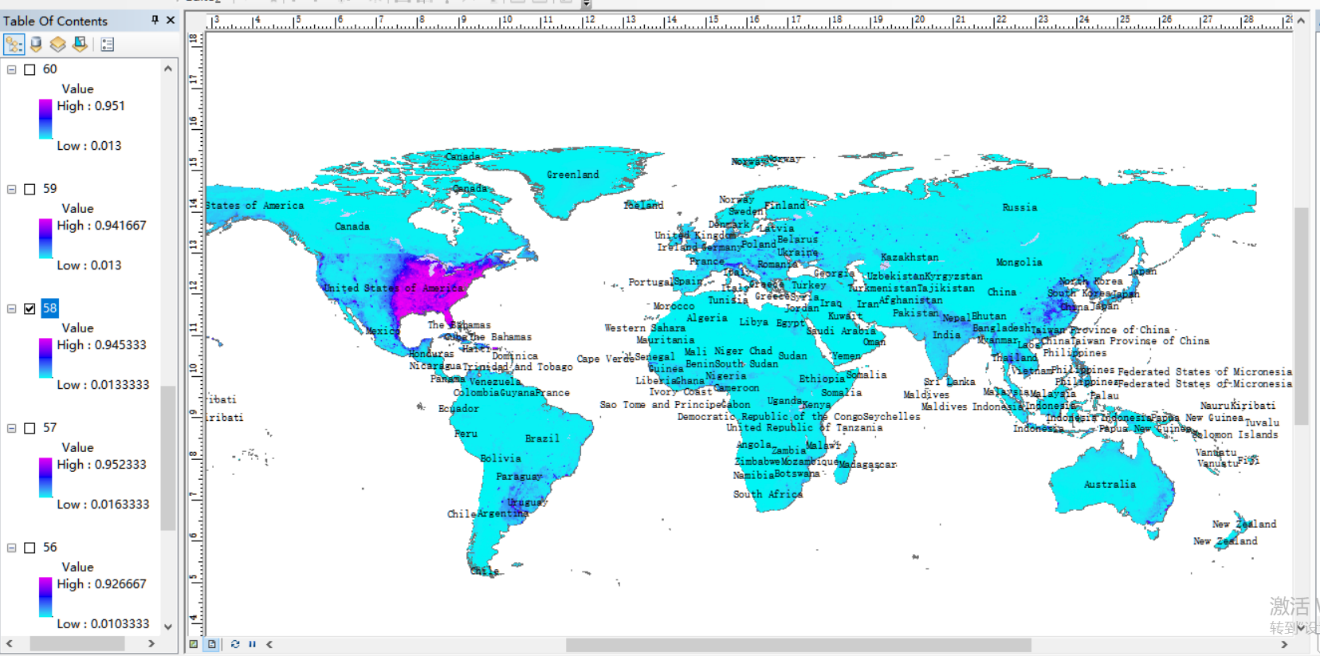


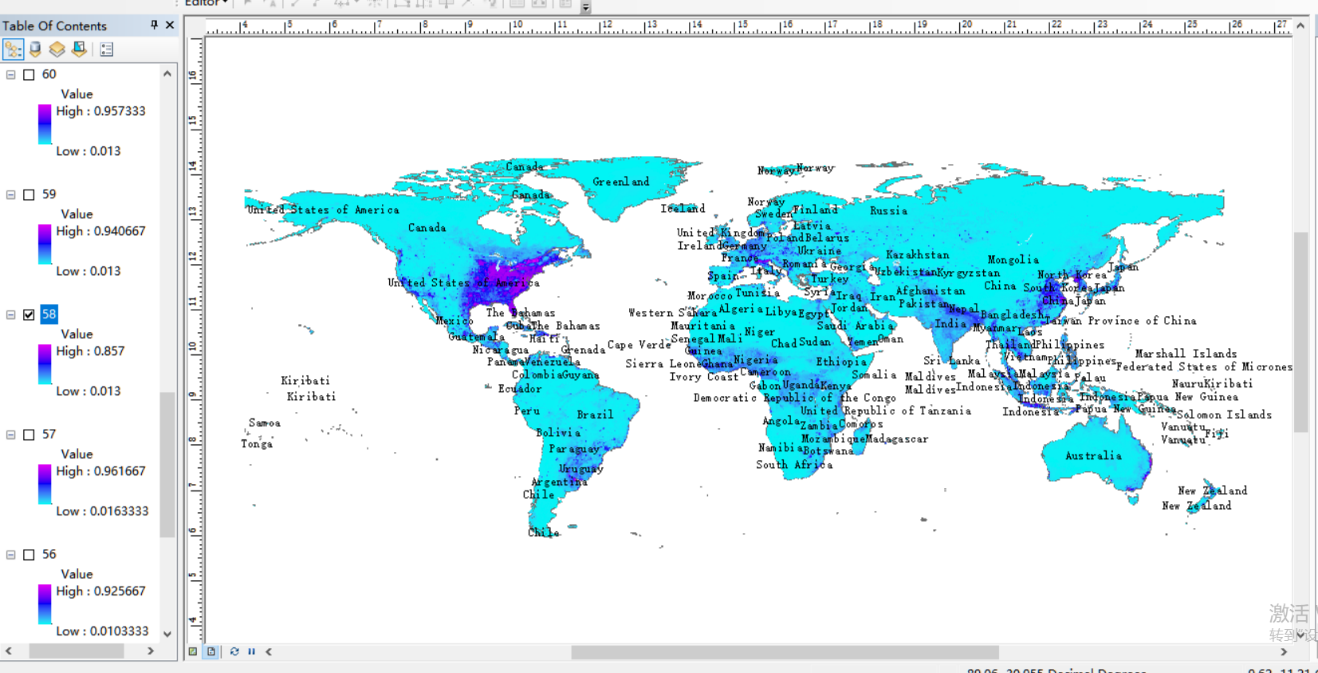


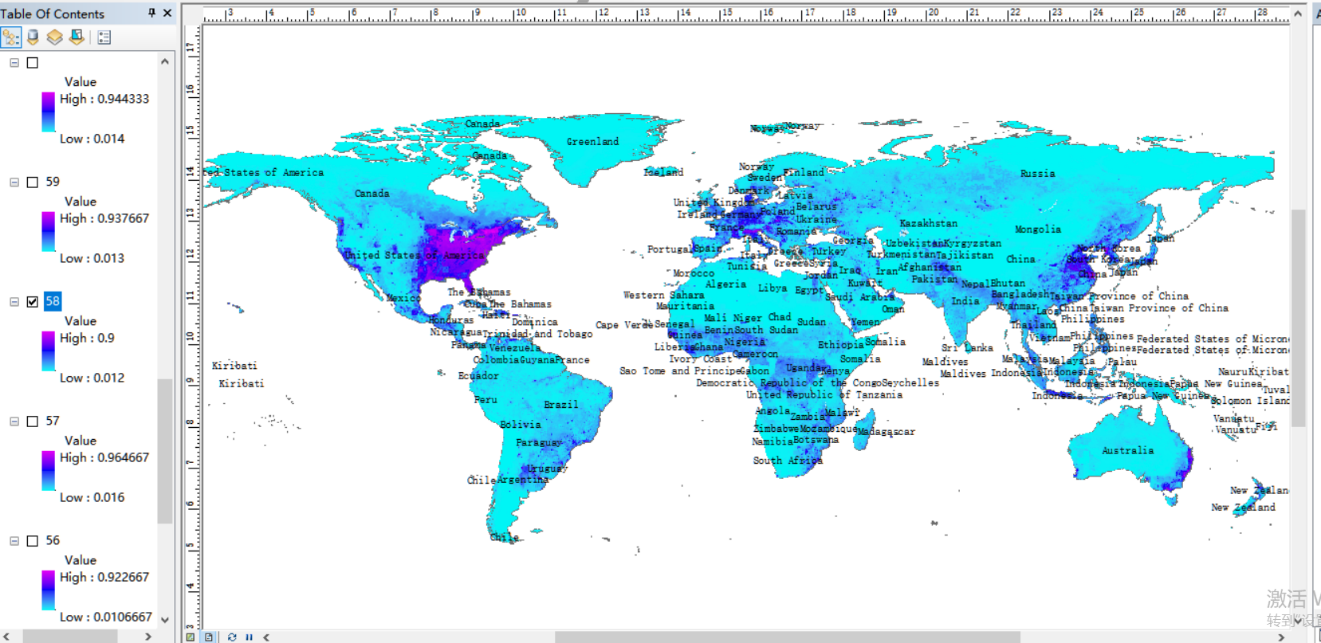


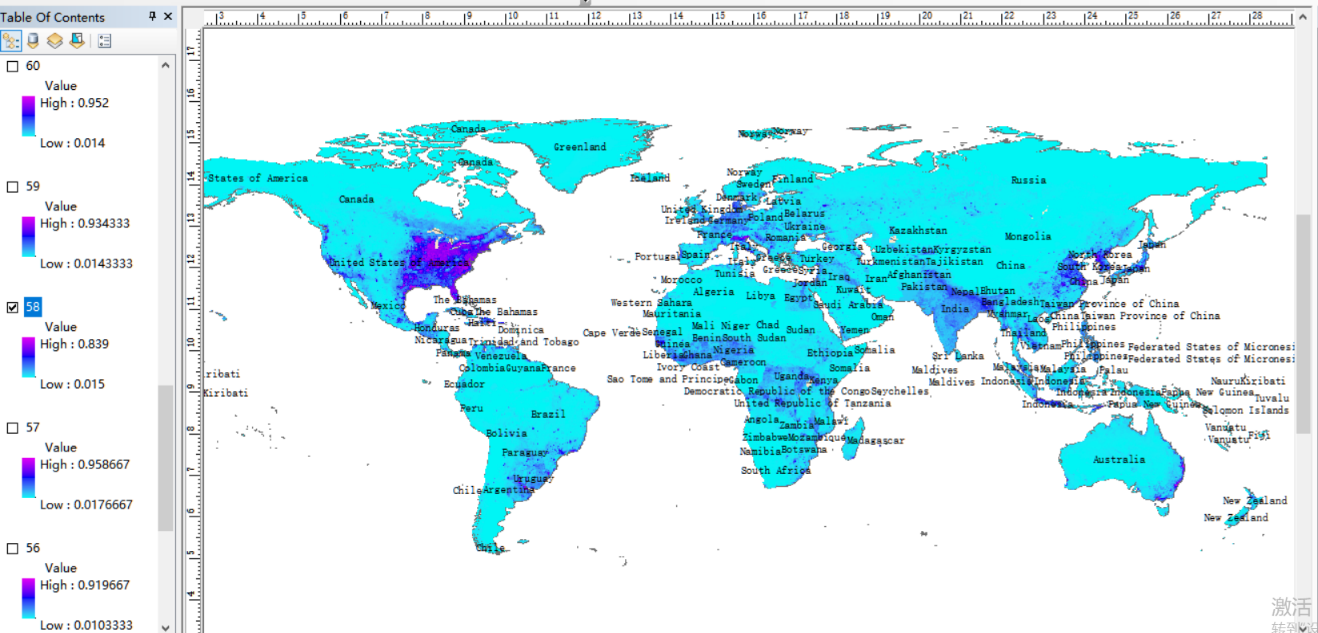


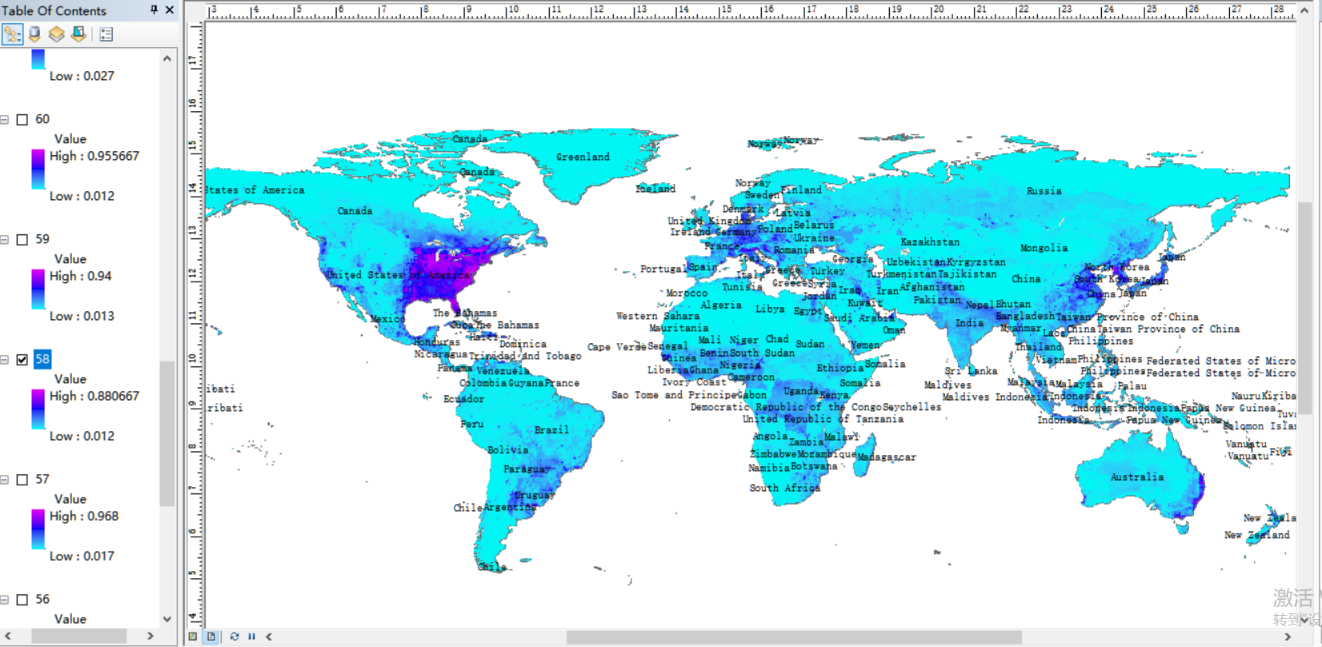


*Vespula vulgaris* Current, F126, F585, M126 and M585 in this order


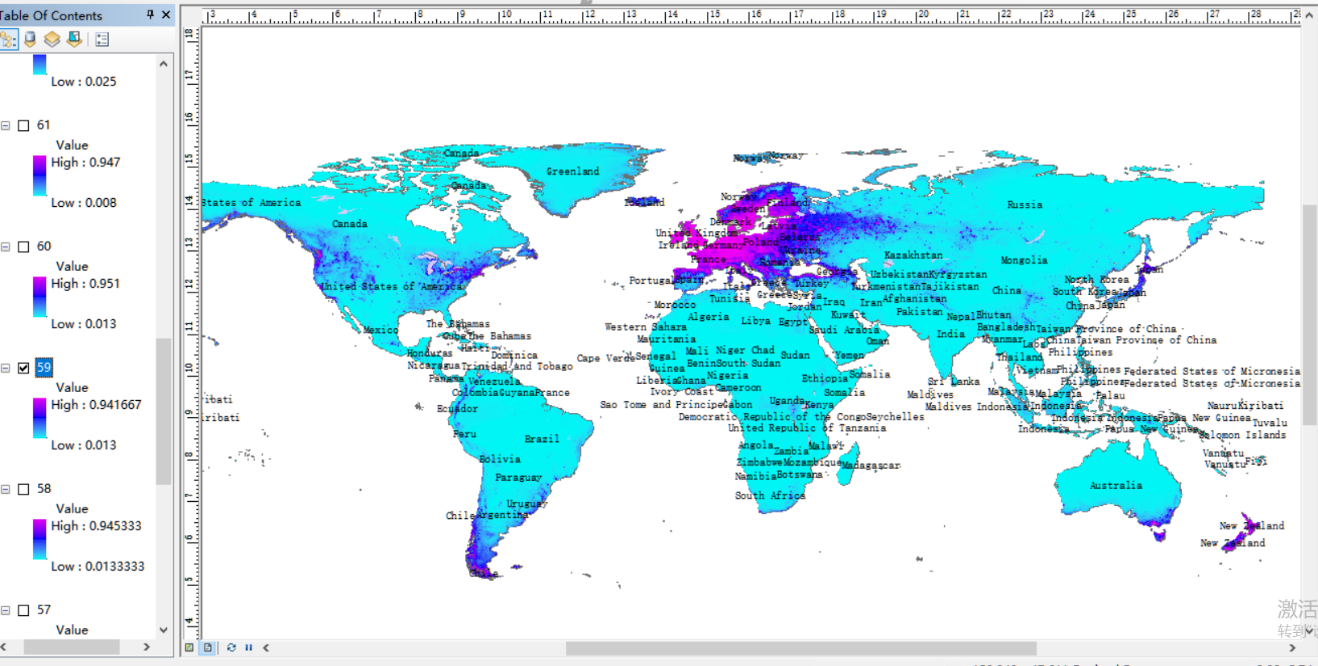


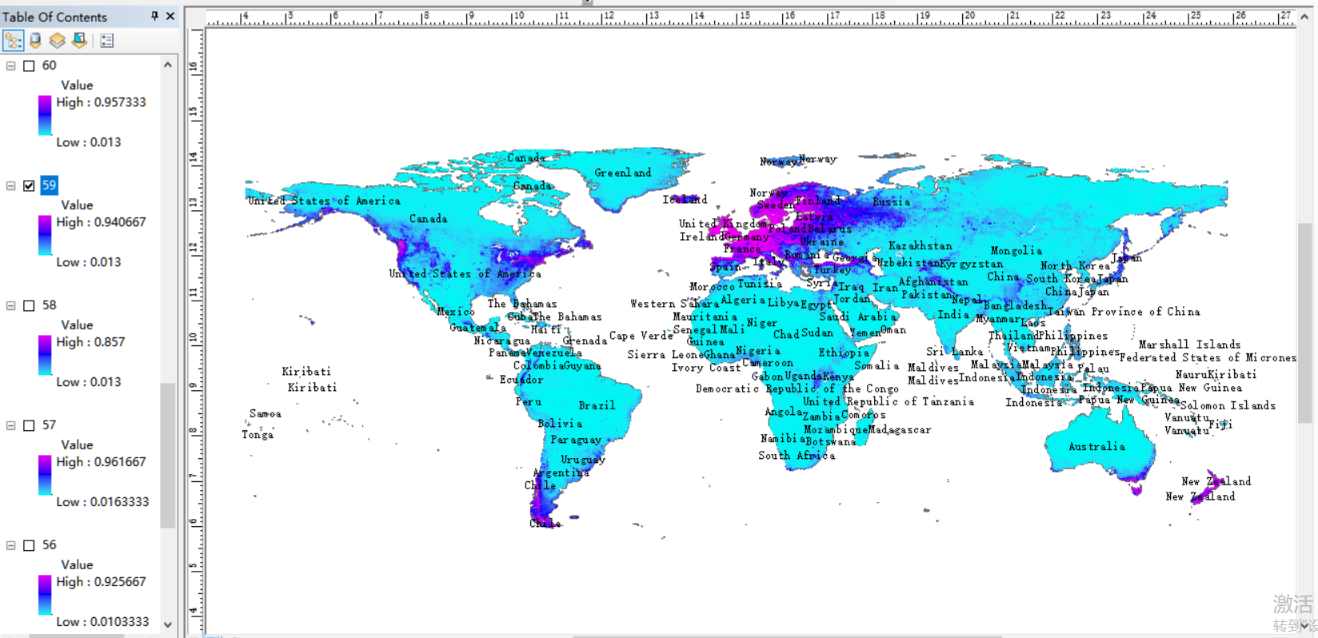


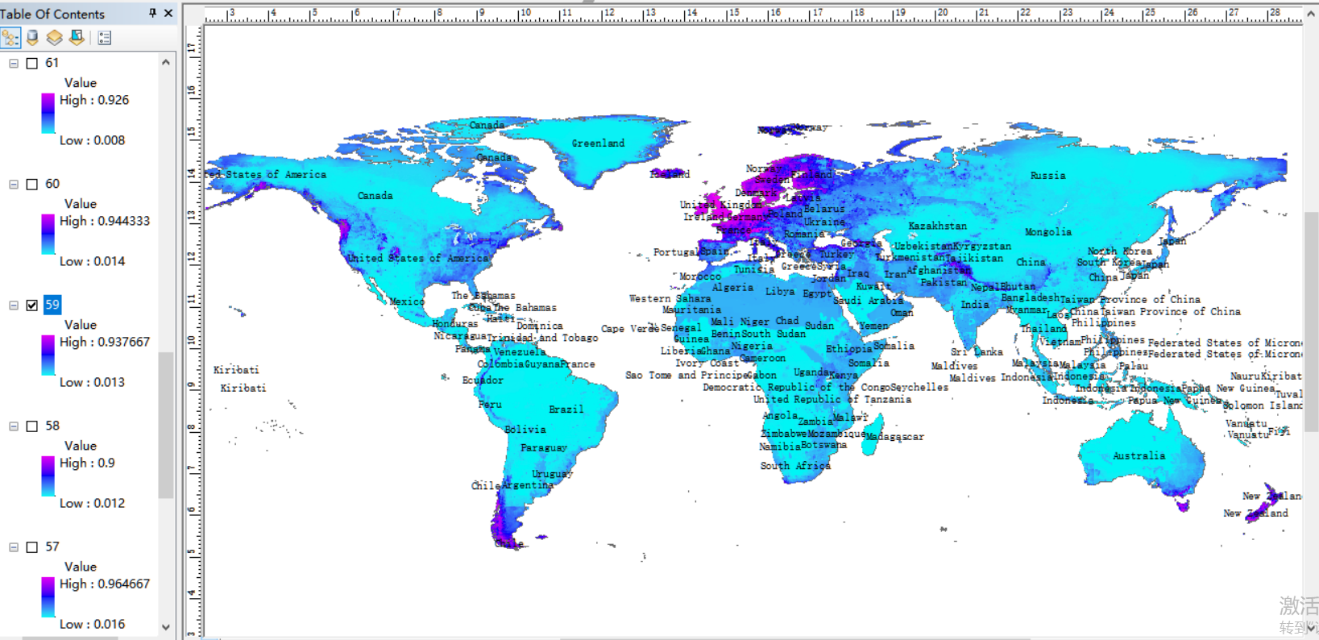


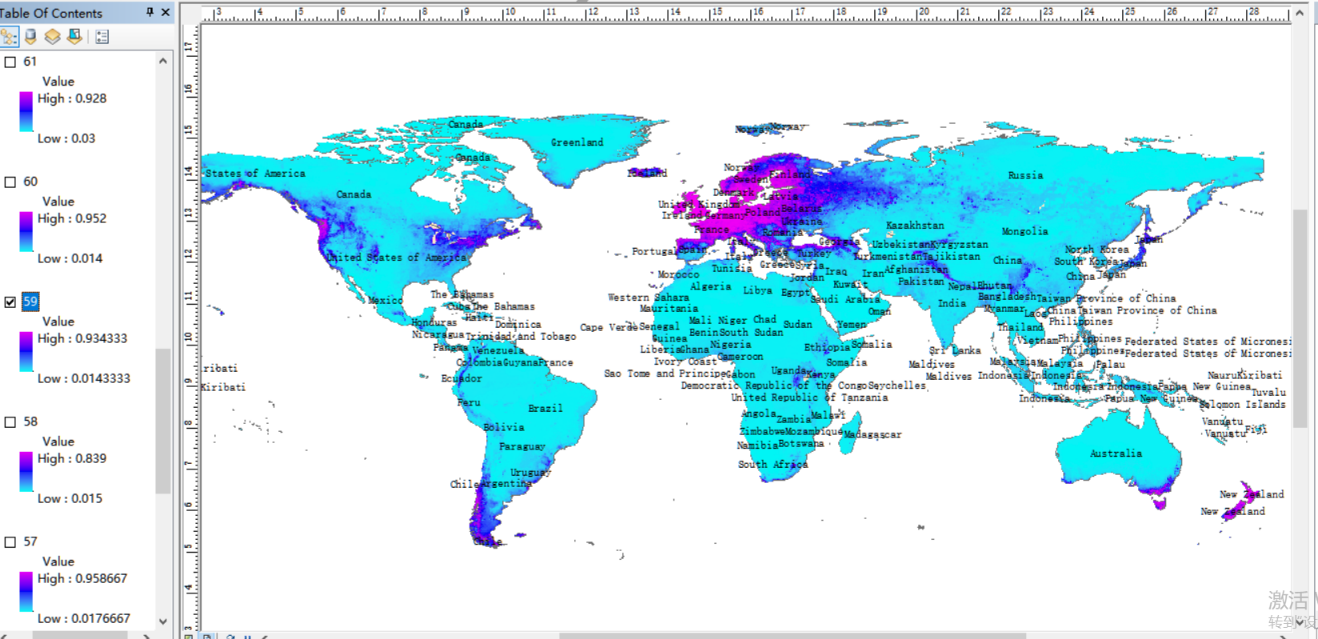


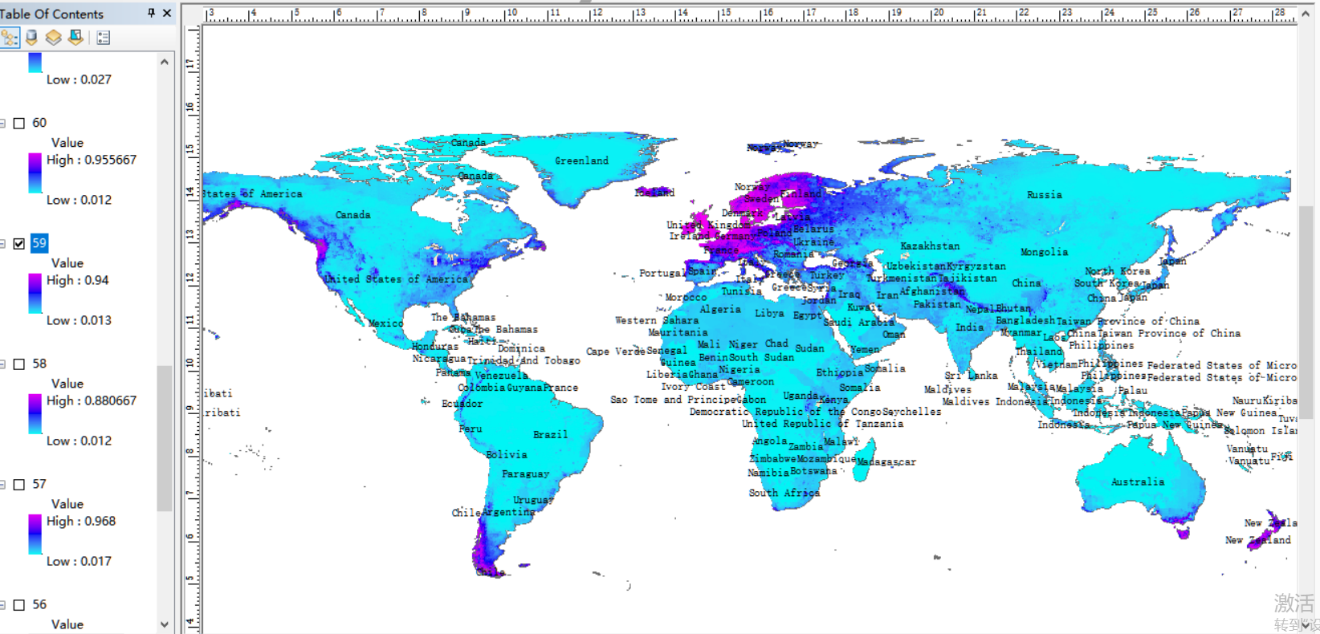


*Anoplolepis gracilipes*  Current, F126, F585, M126 and M585 in this order


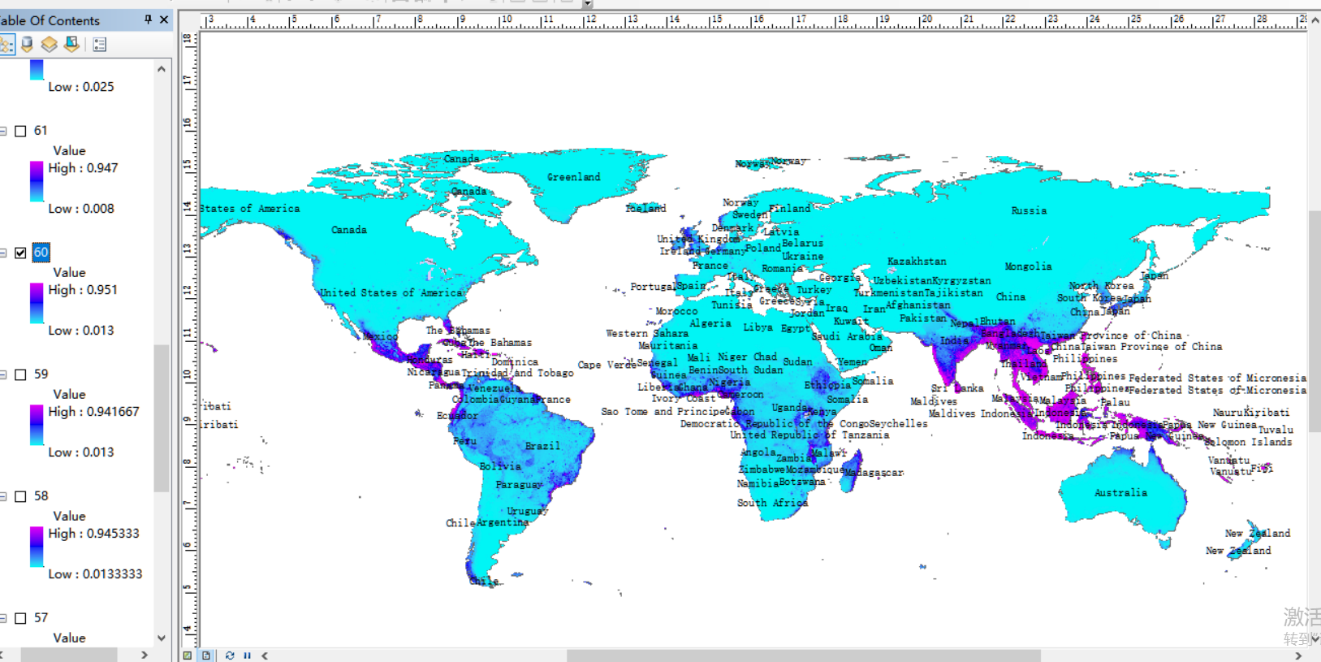


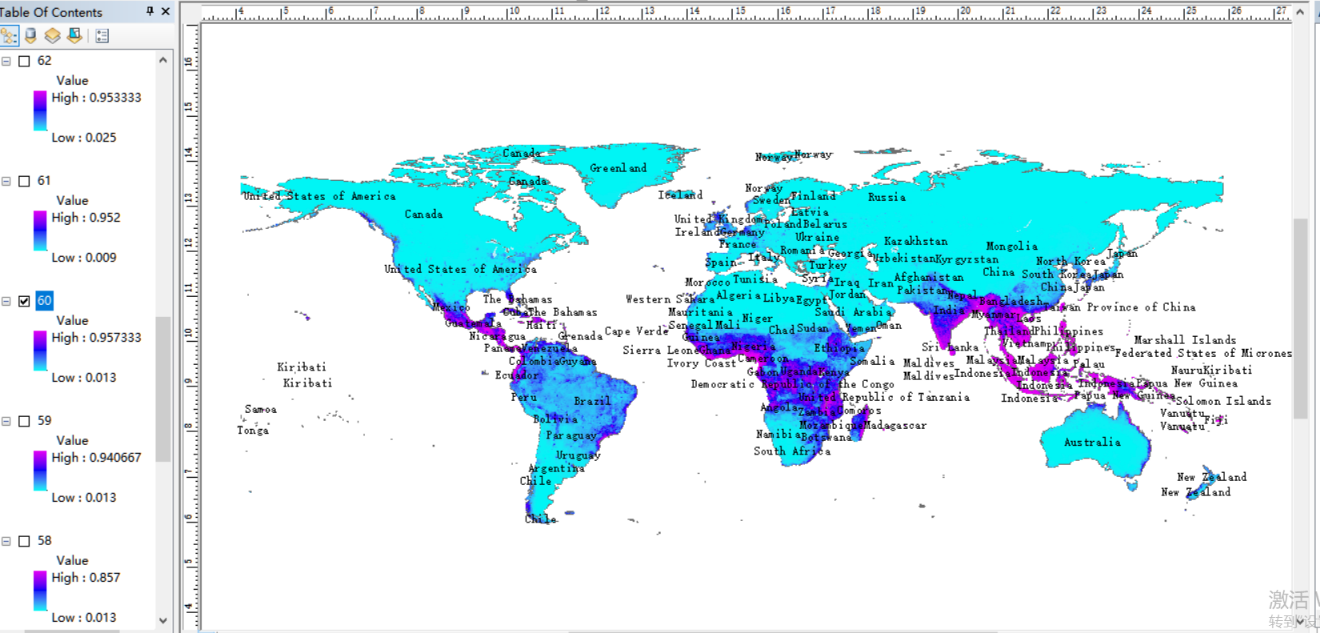


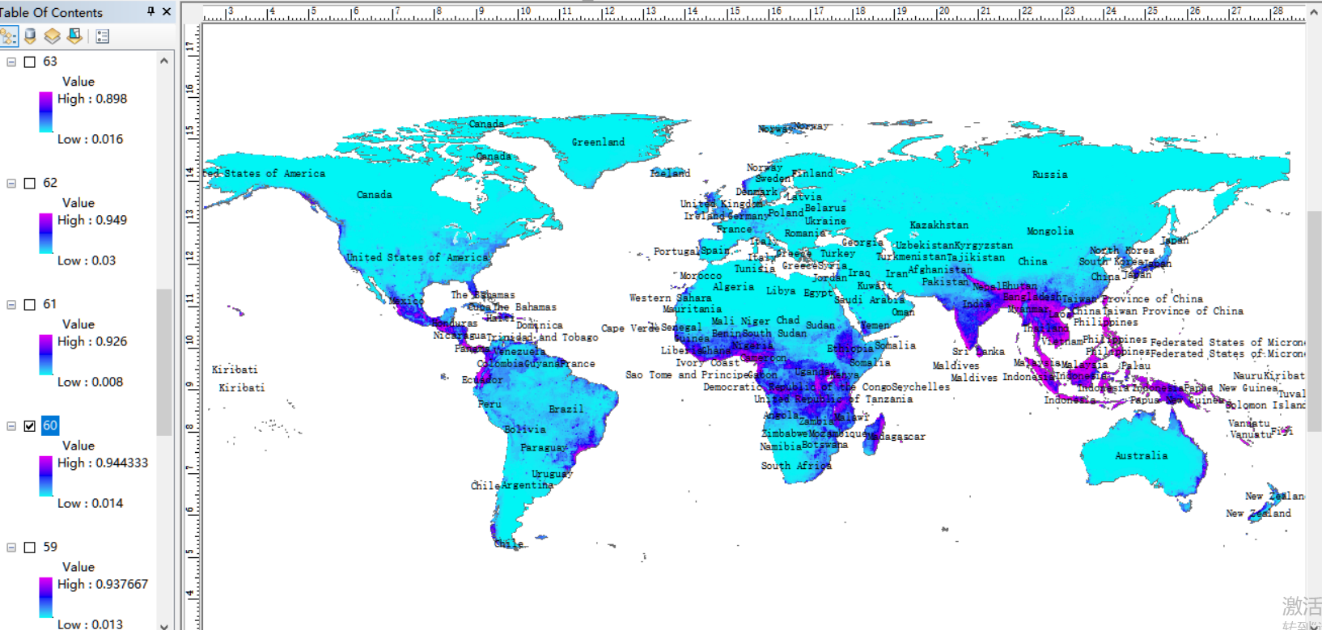


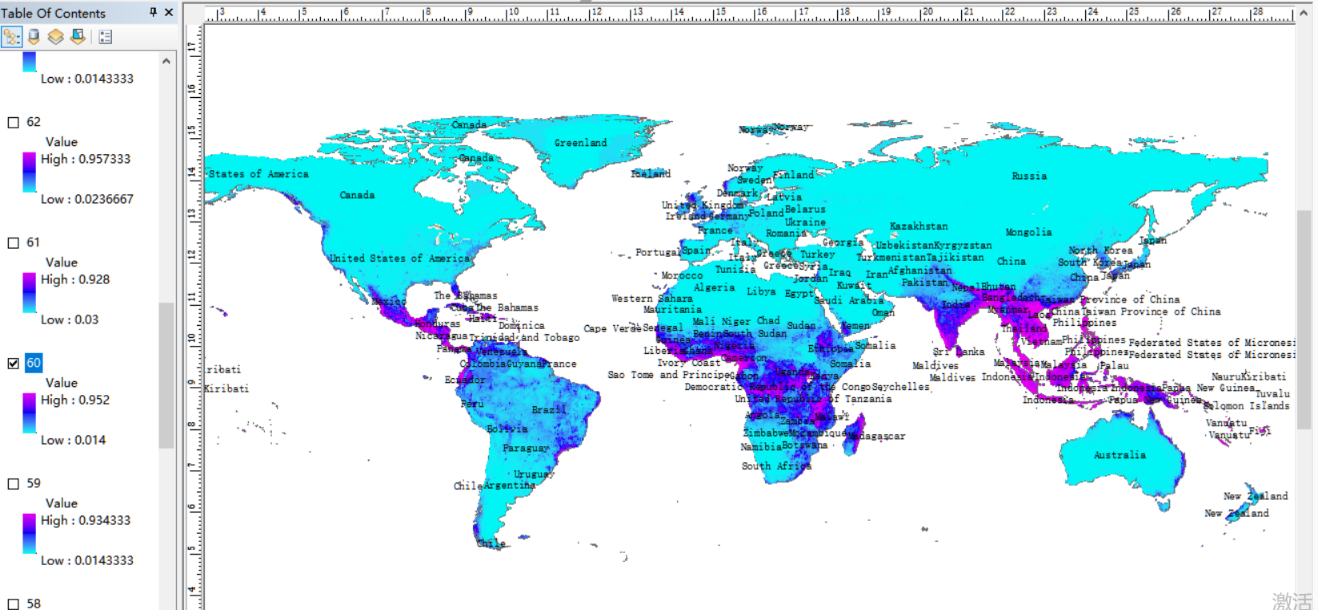


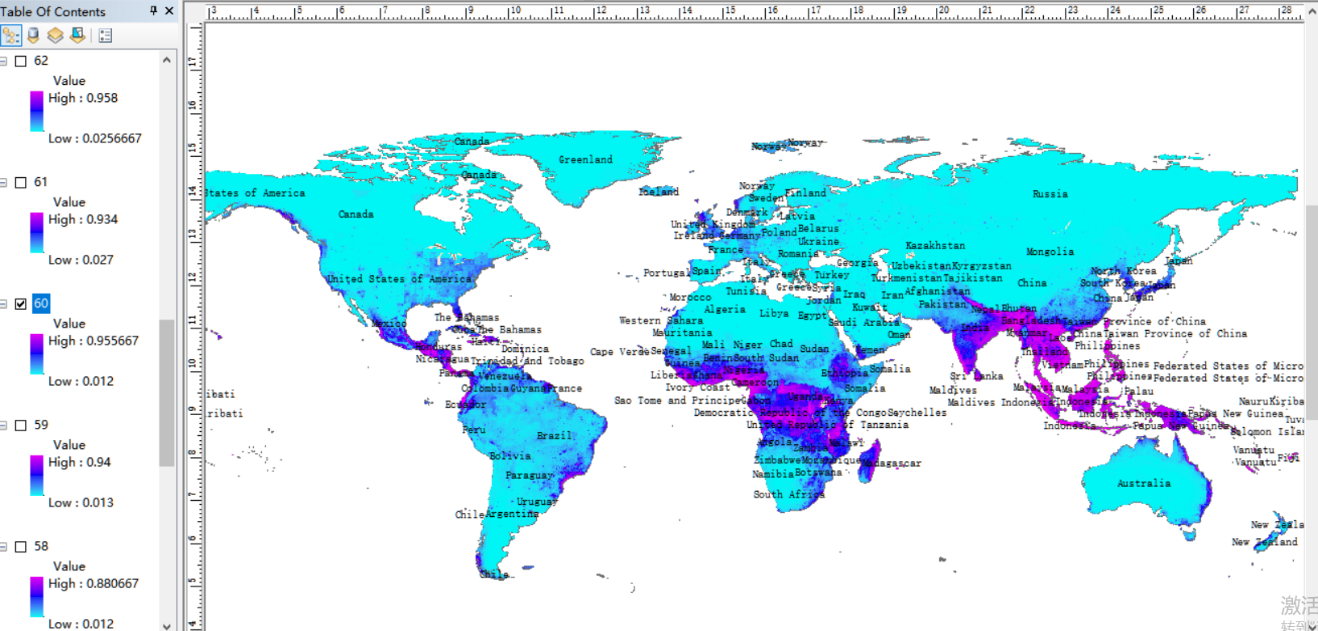


*Cinara cupressi* Current, F126, F585, M126 and M585 in this order


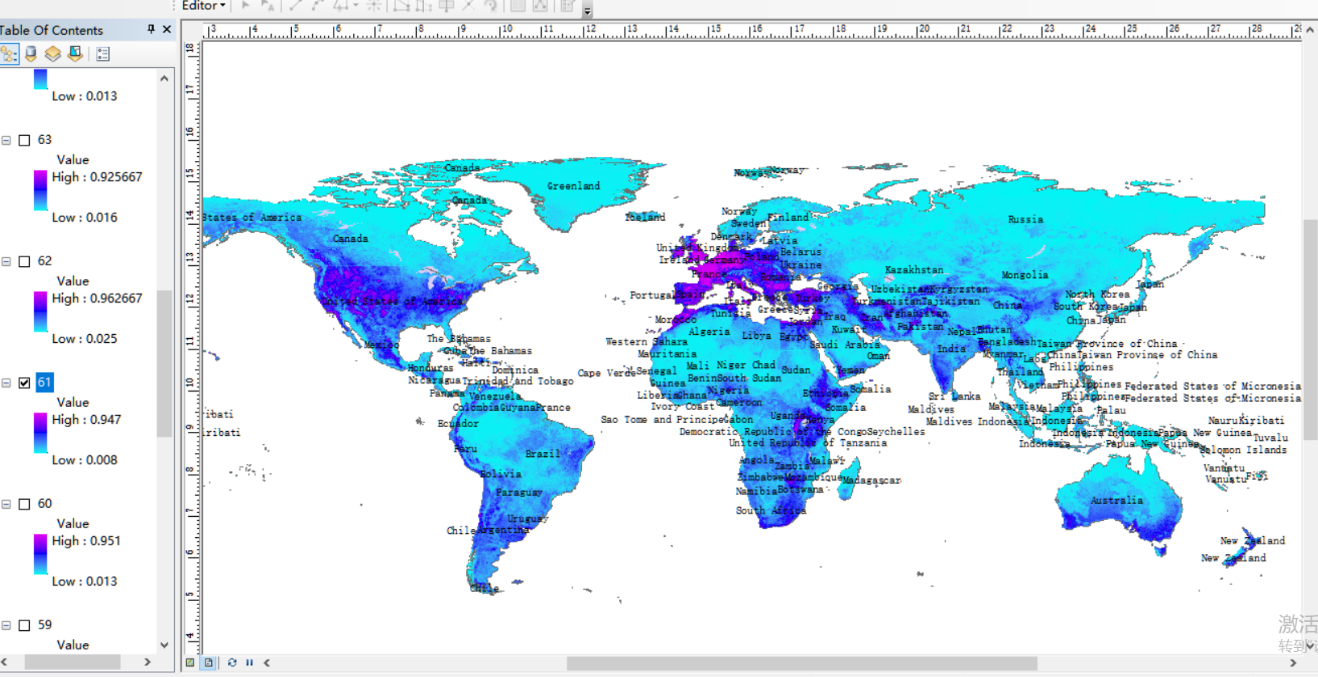


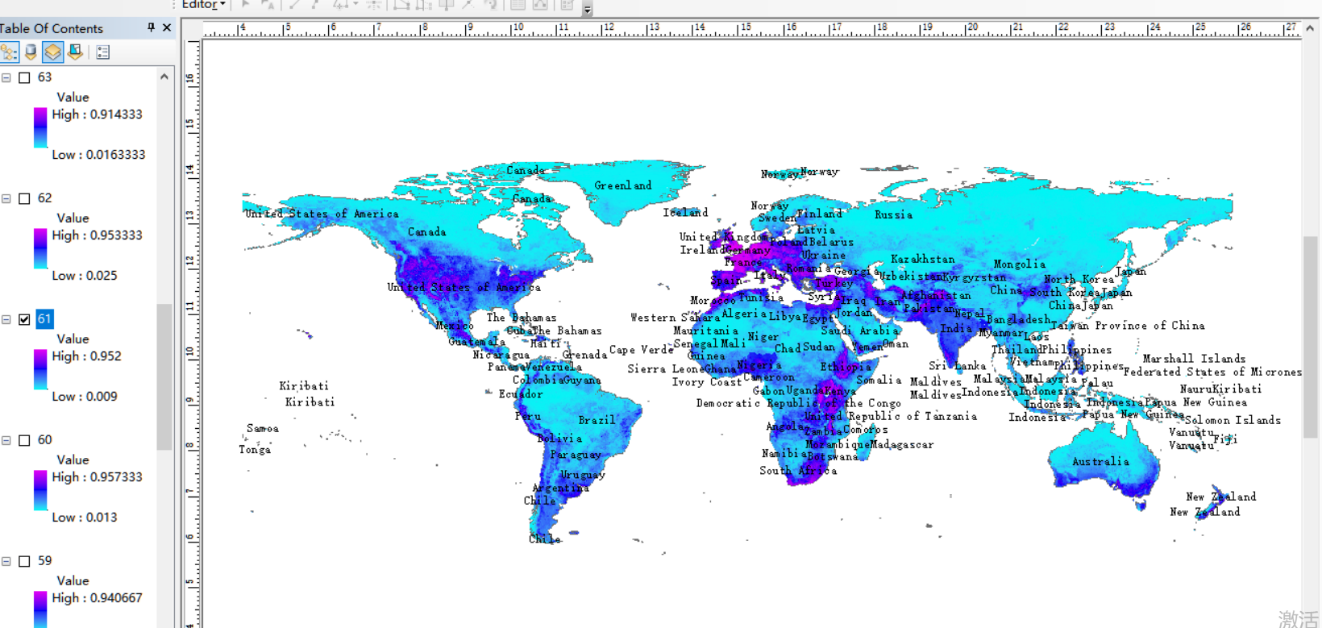


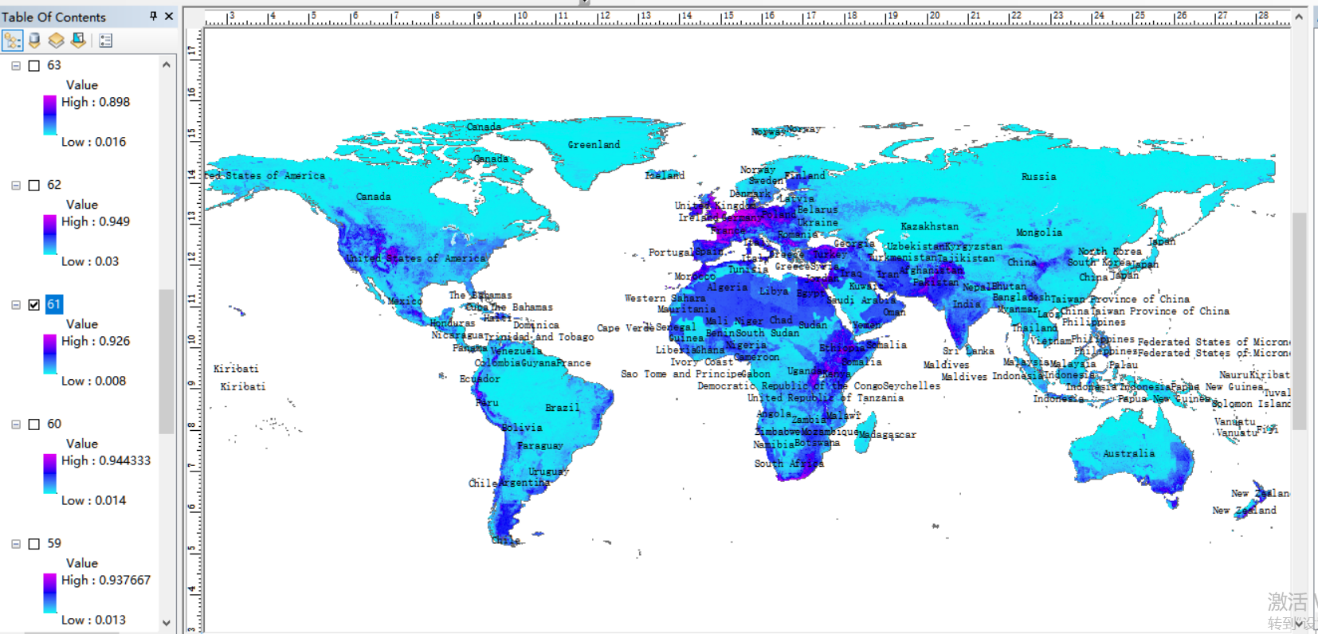


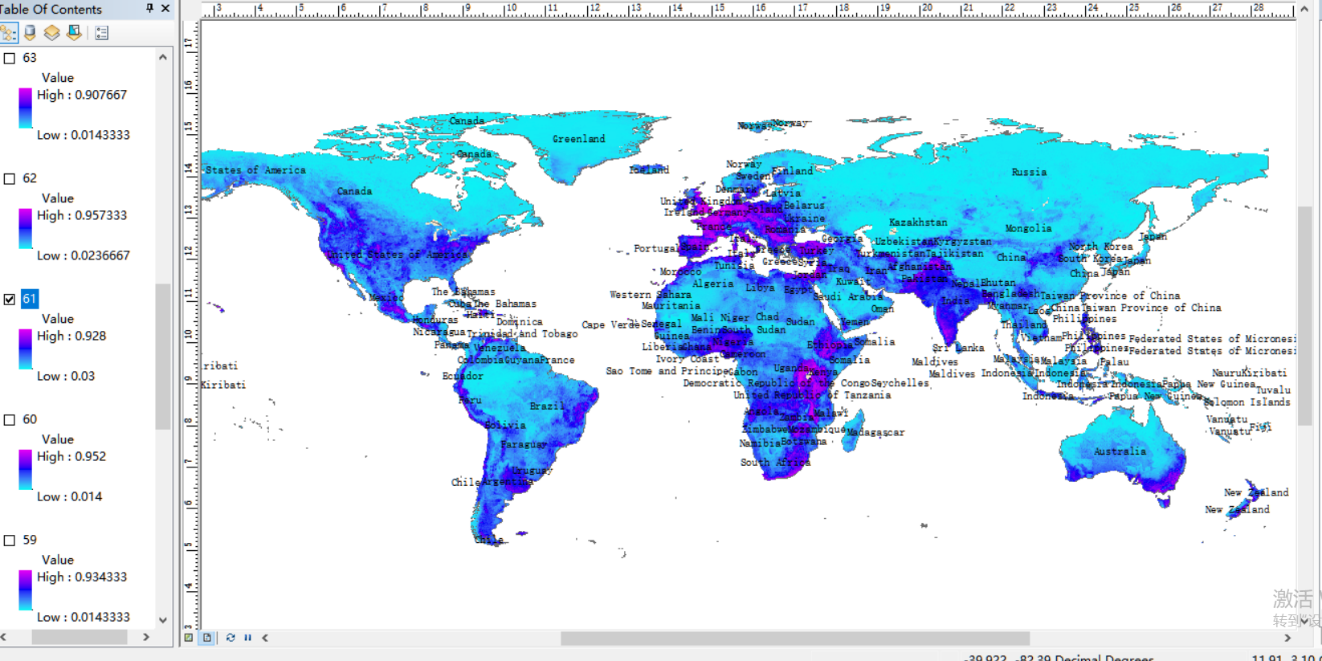


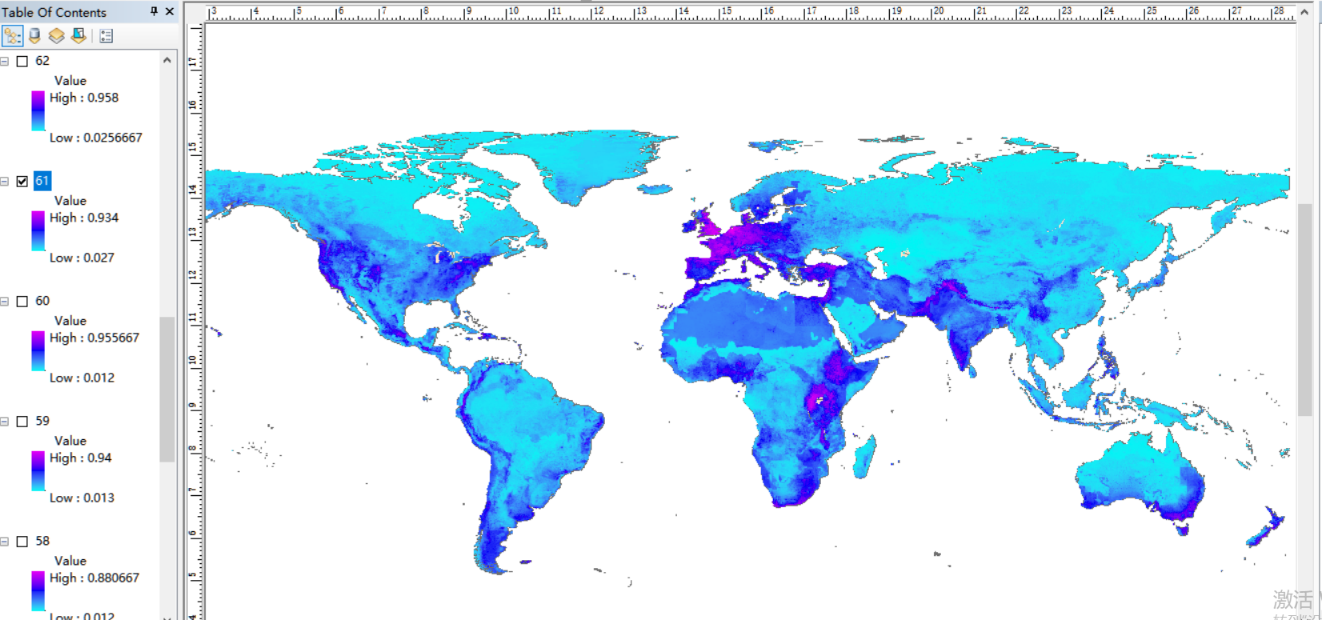


*Platydemus manokwari* Current, F126, F585, M126 and M585 in this order


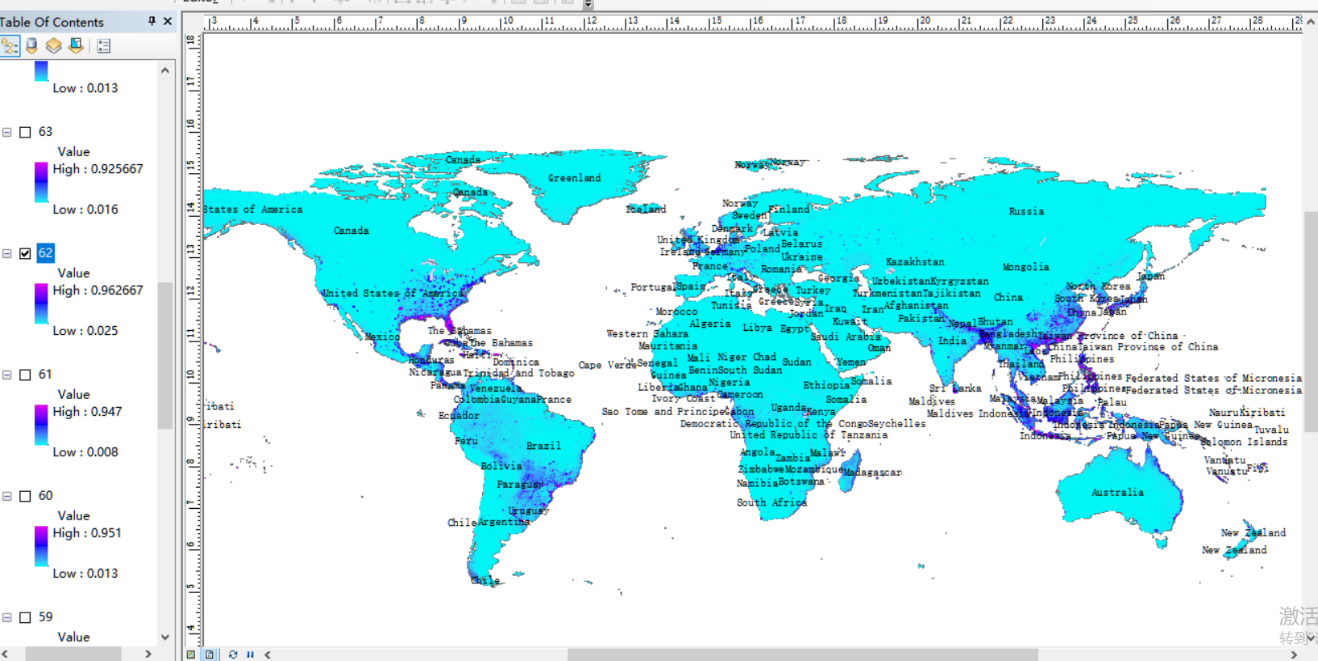


*
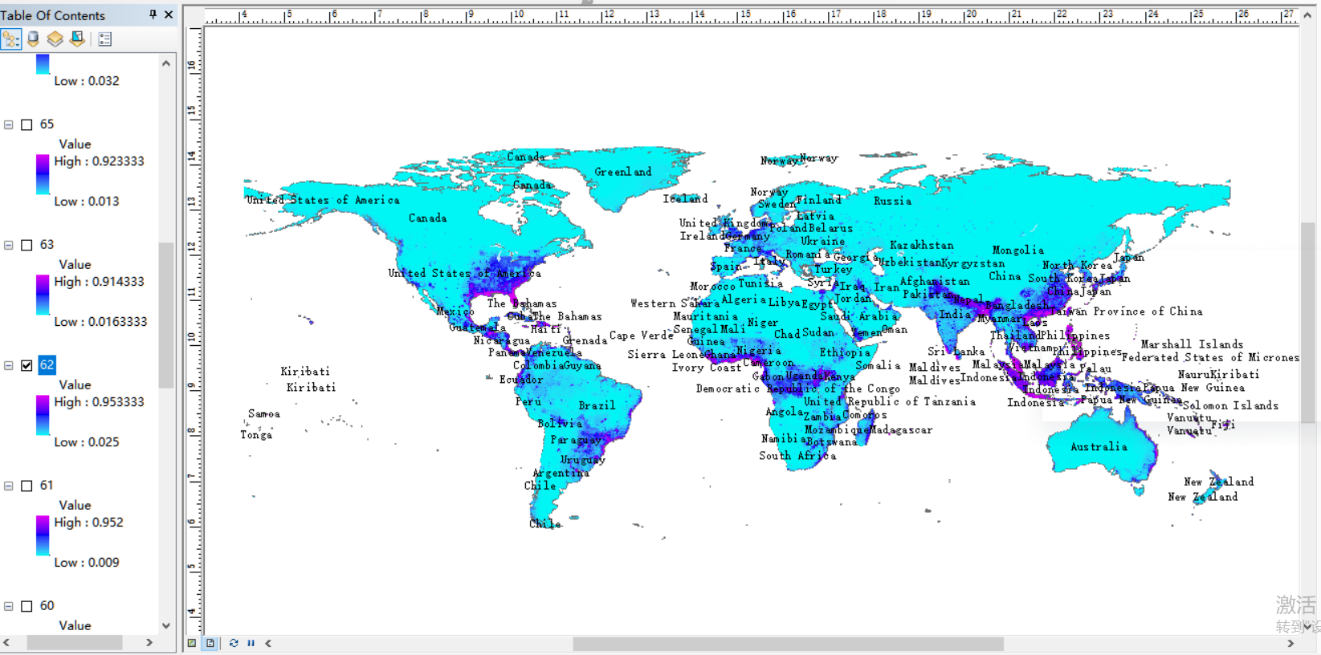
*

*
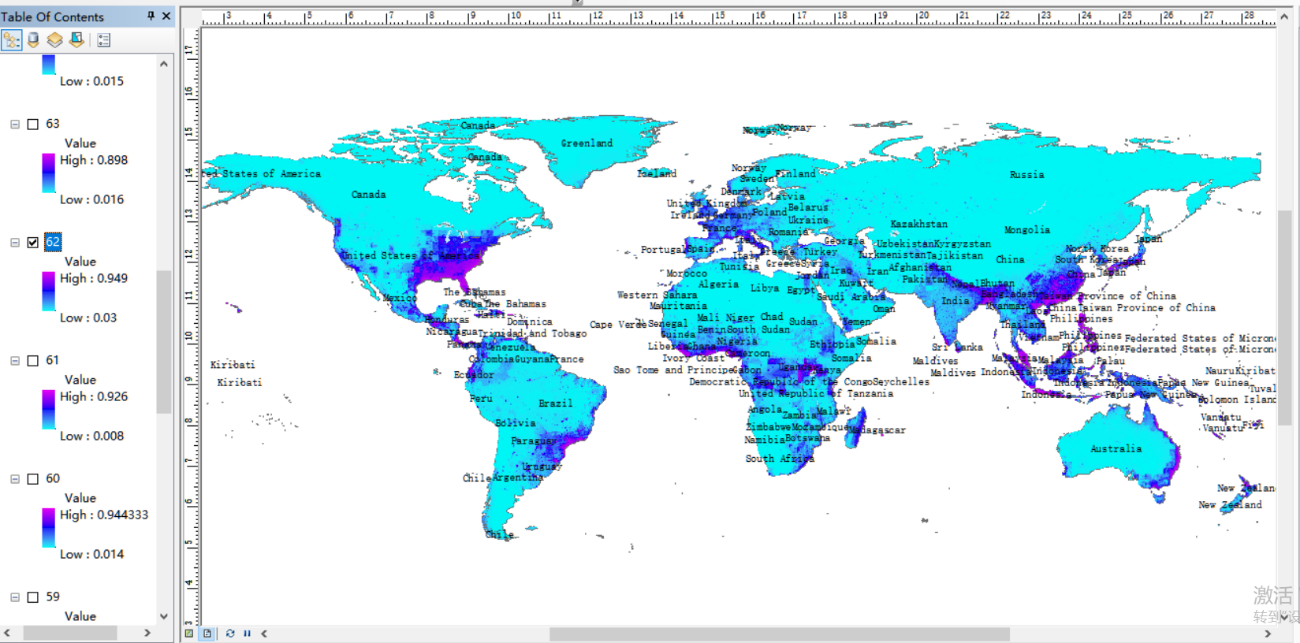
*

*
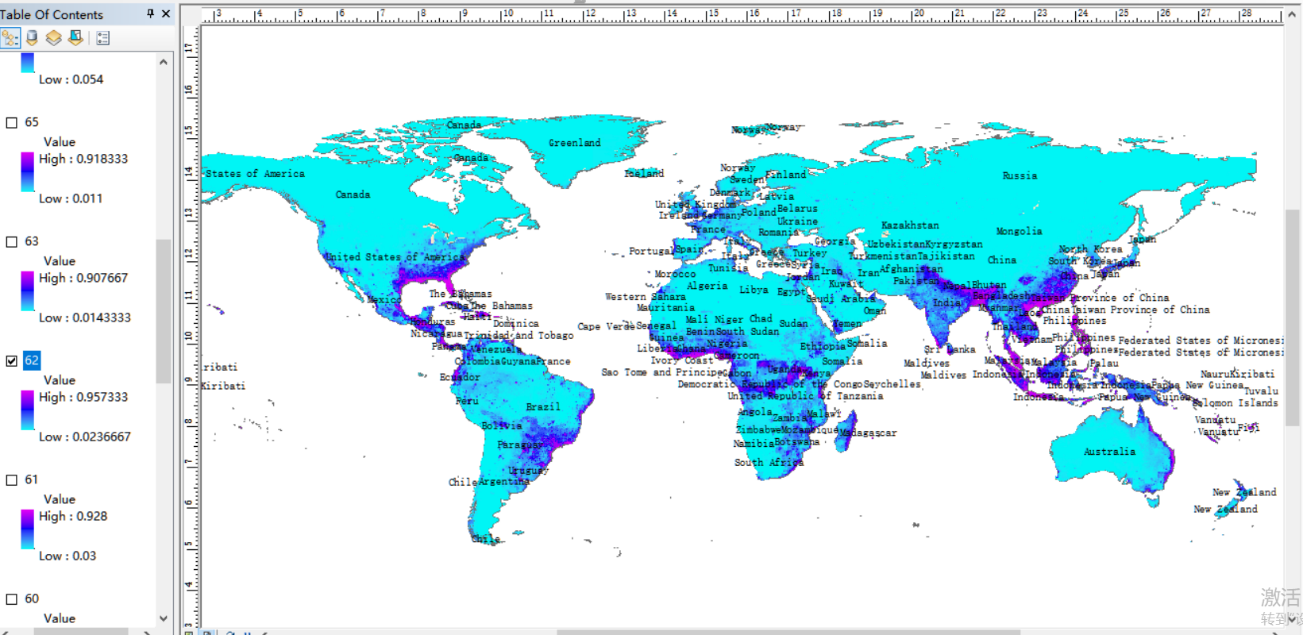
*

*
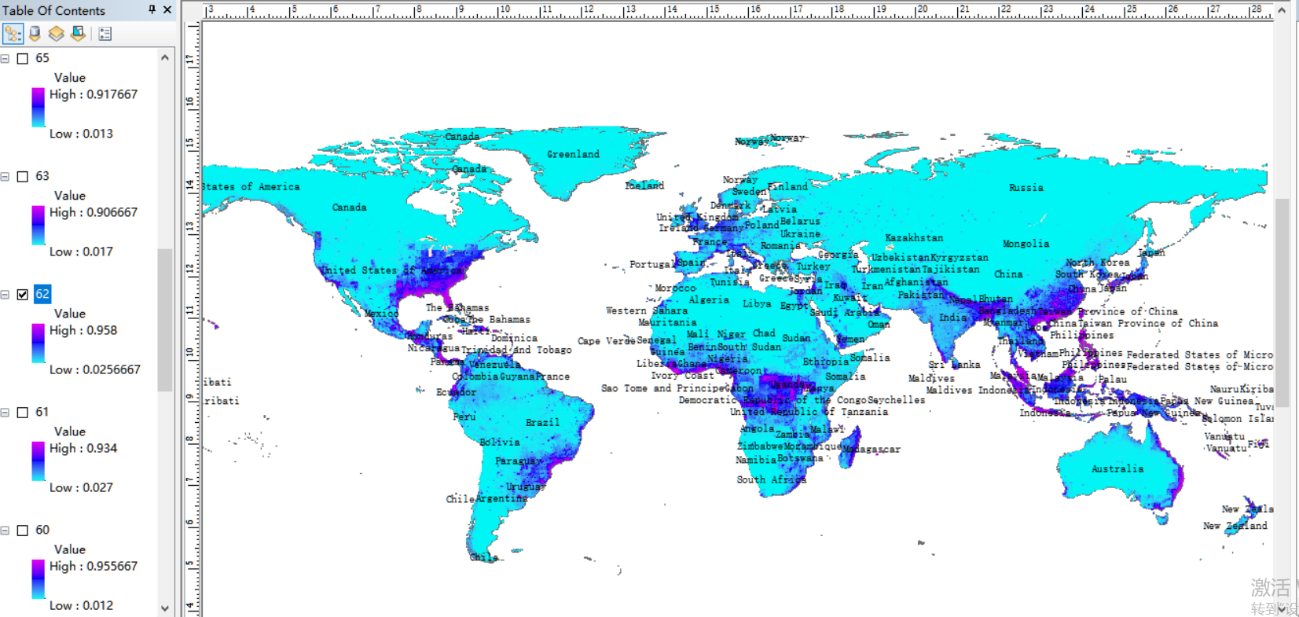
*

*Coptotermes formosanus shiraki*  Current, F126, F585, M126 and M585 in this order


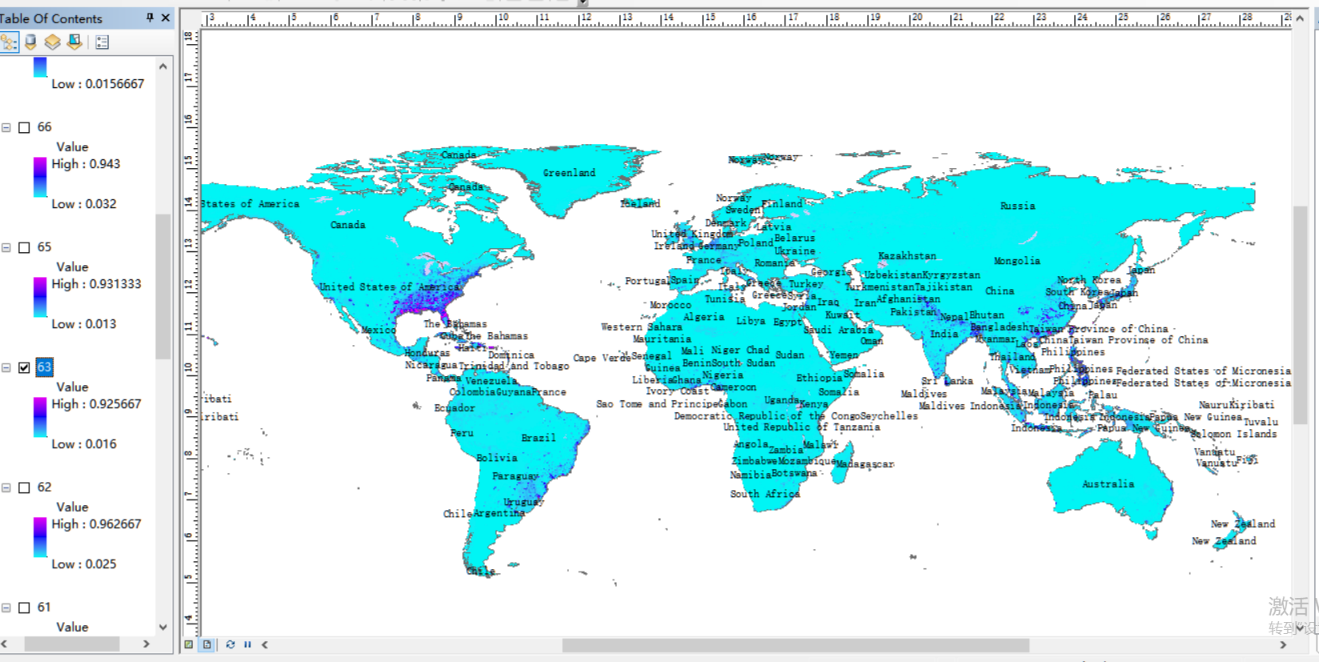


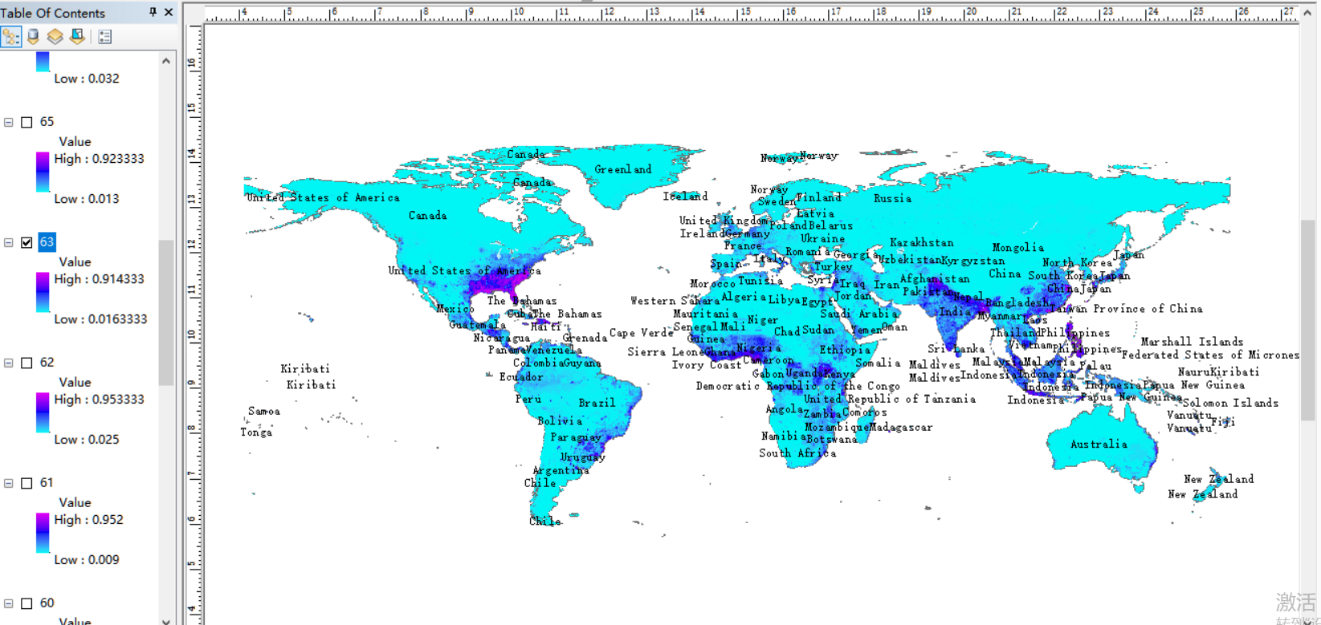


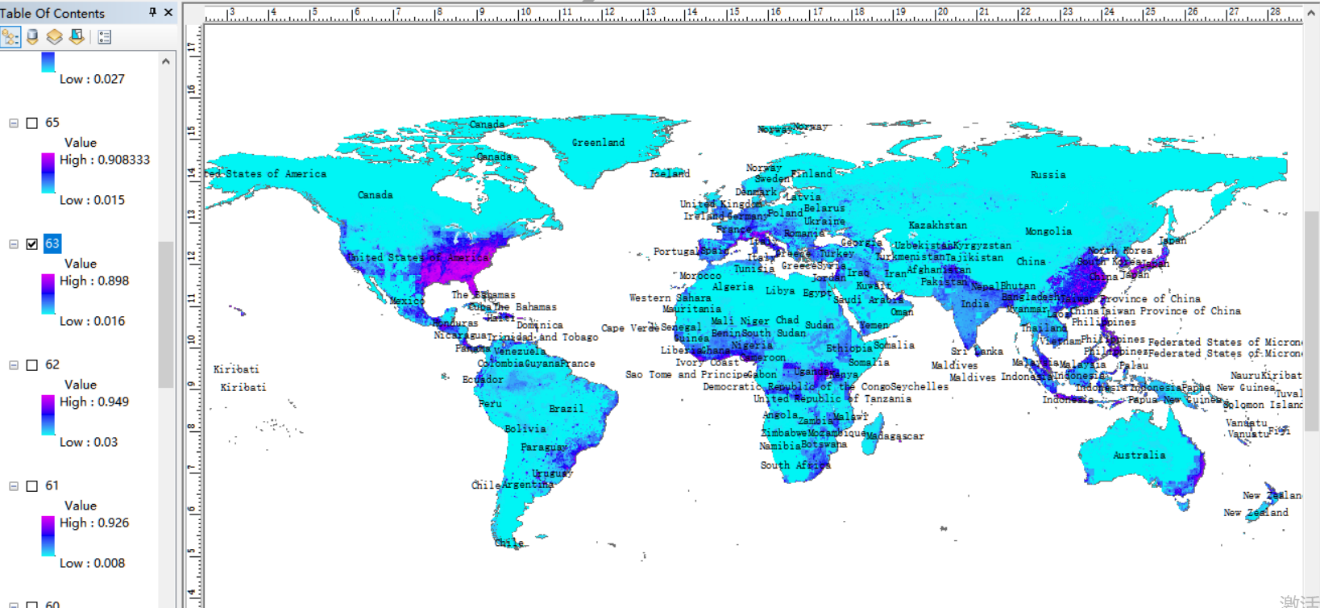


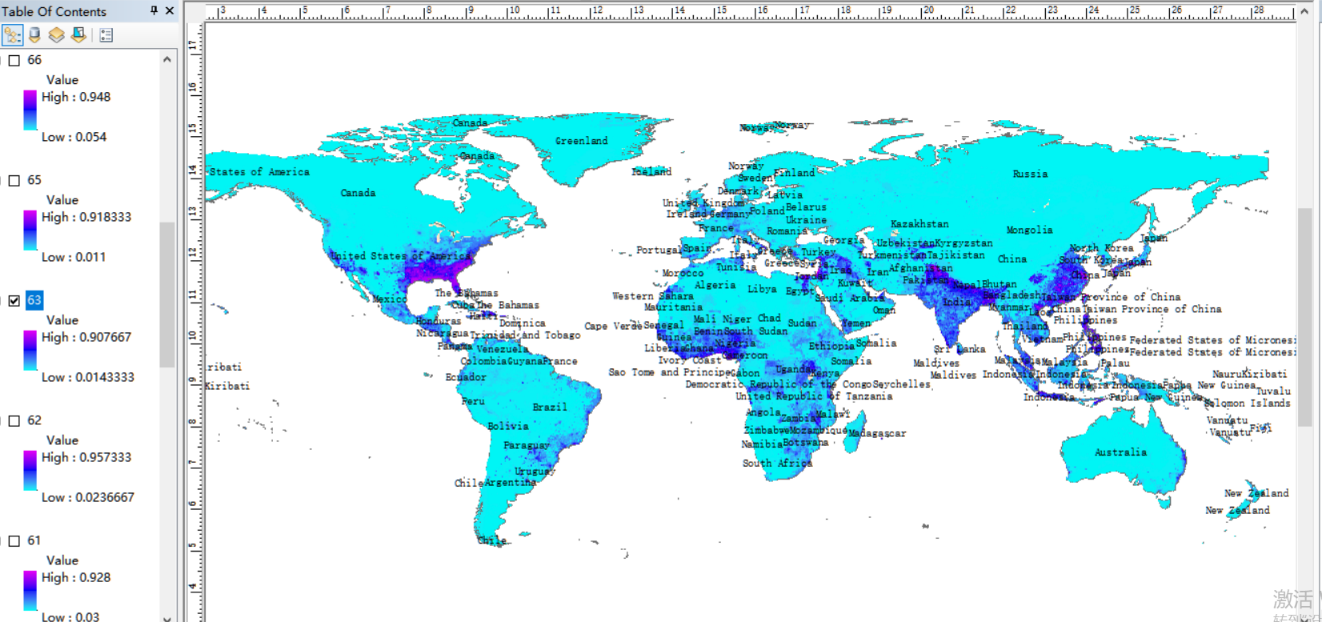


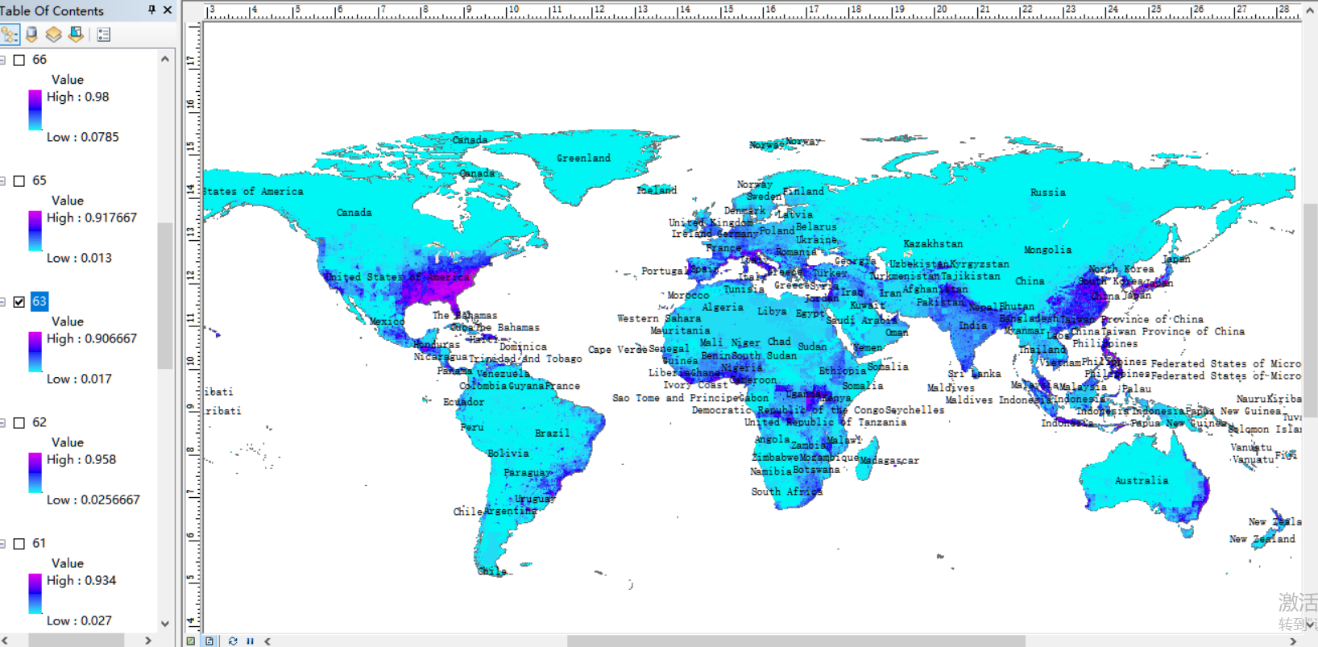


*Lymantria dispar* Current, F126, F585, M126 and M585 in this order


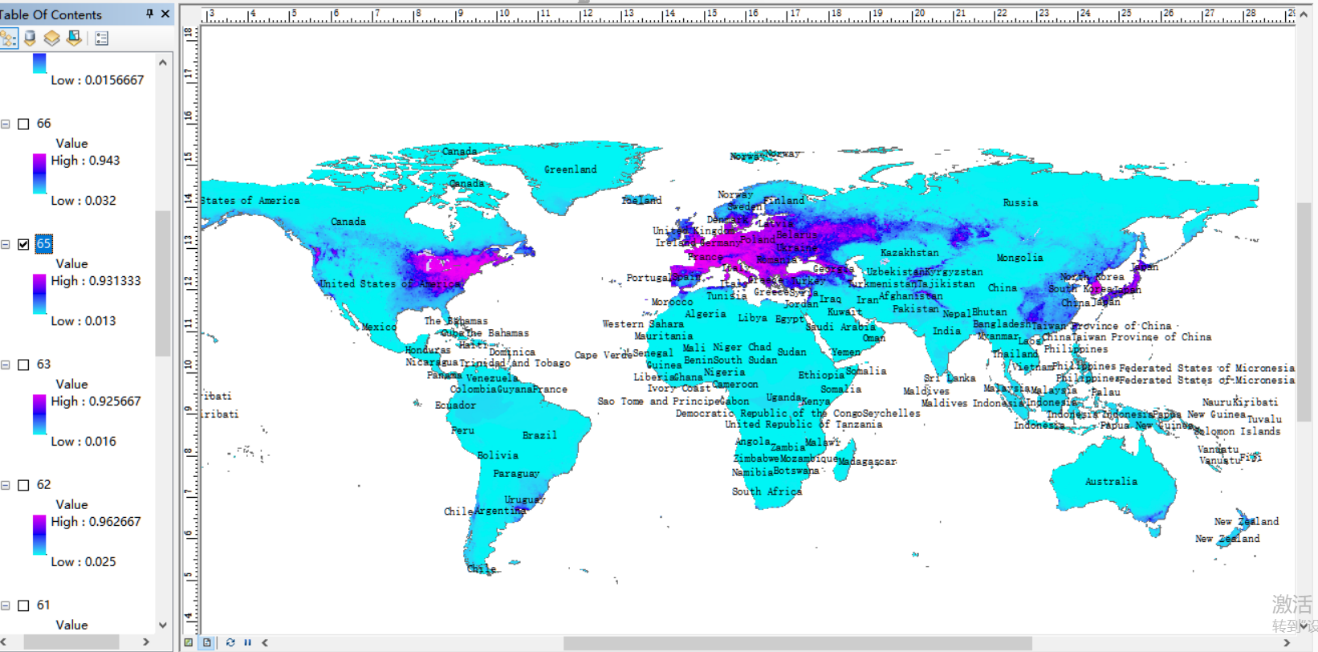


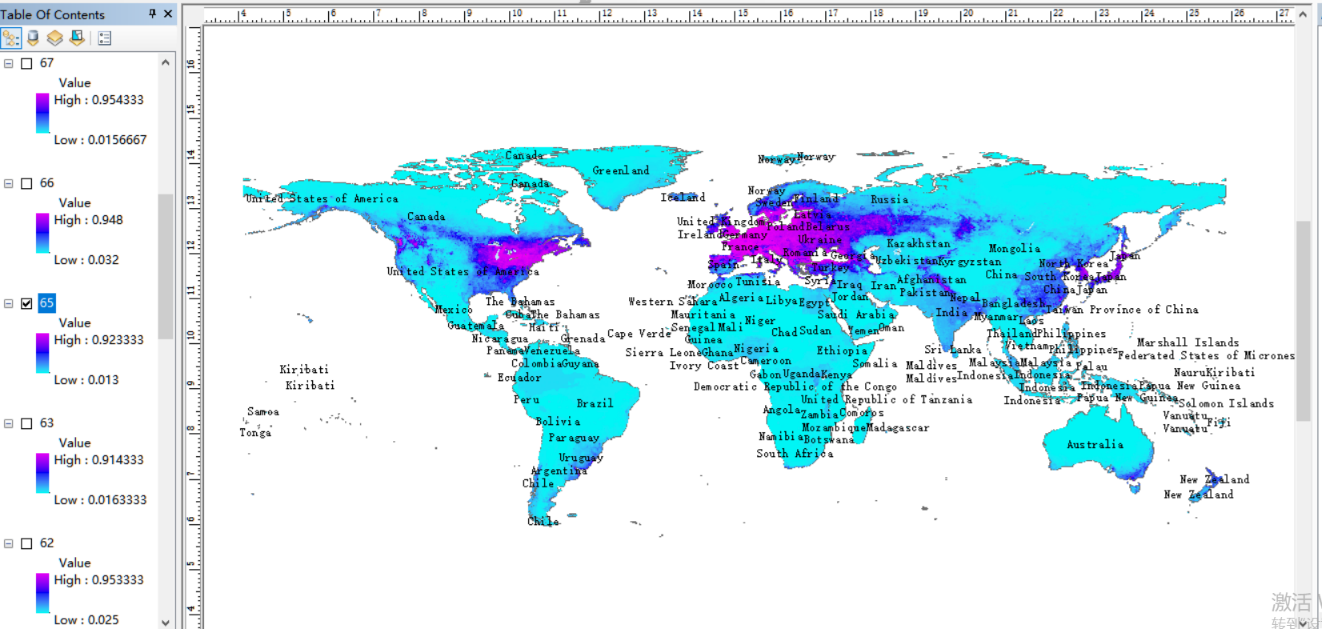


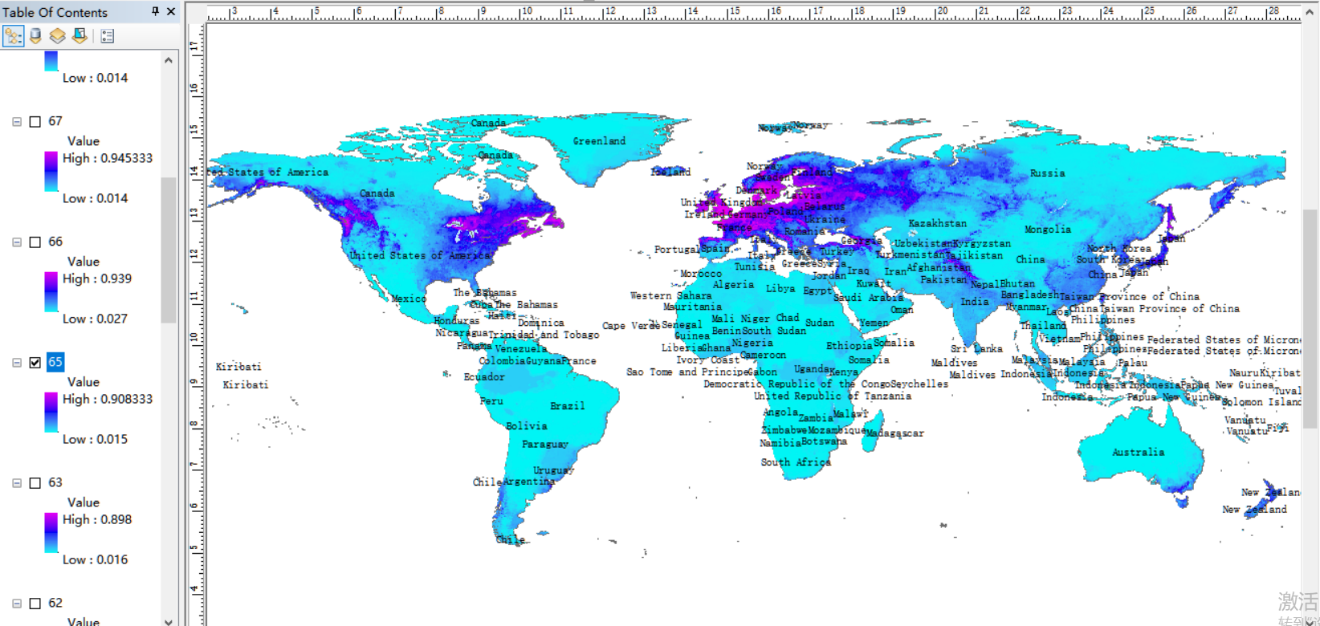


*
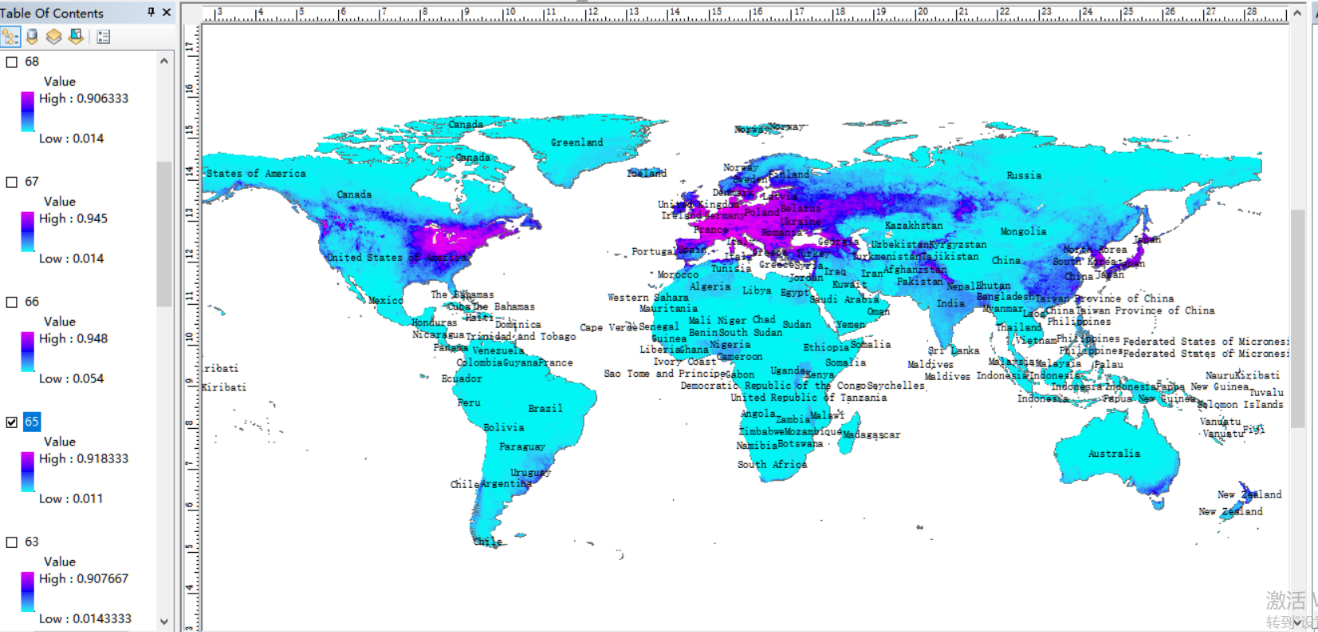
*

*
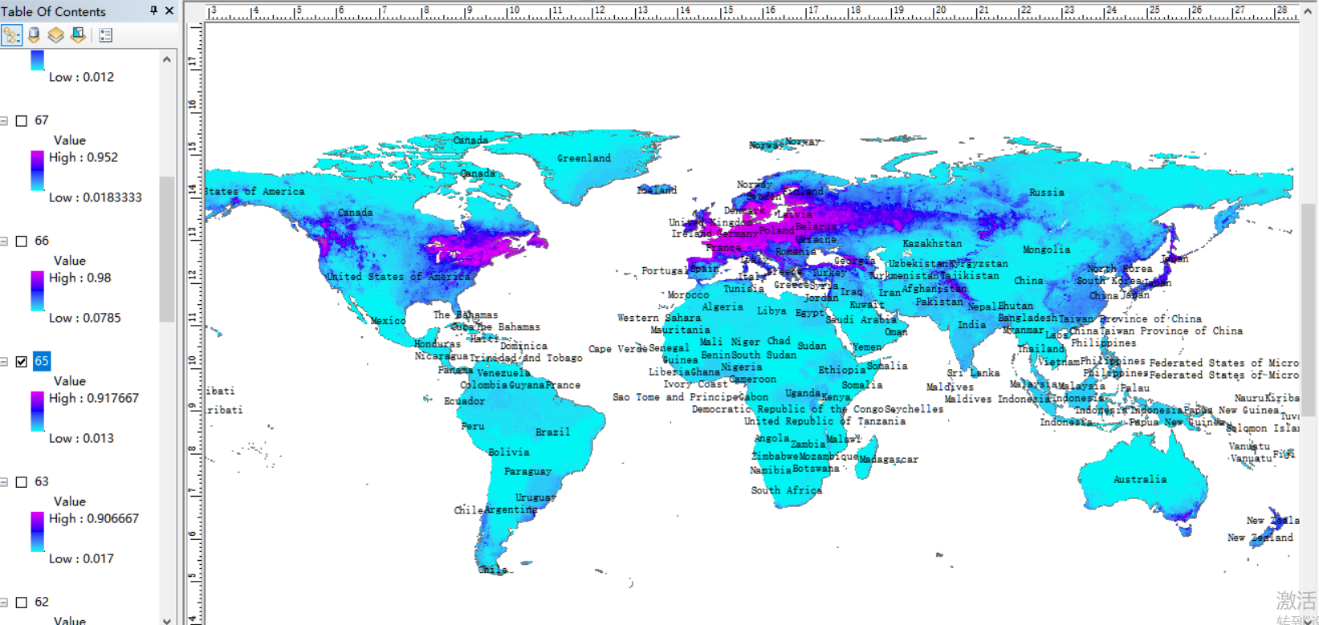
*

*Trogoderma granarium*  Current, F126, F585, M126 and M585 in this order


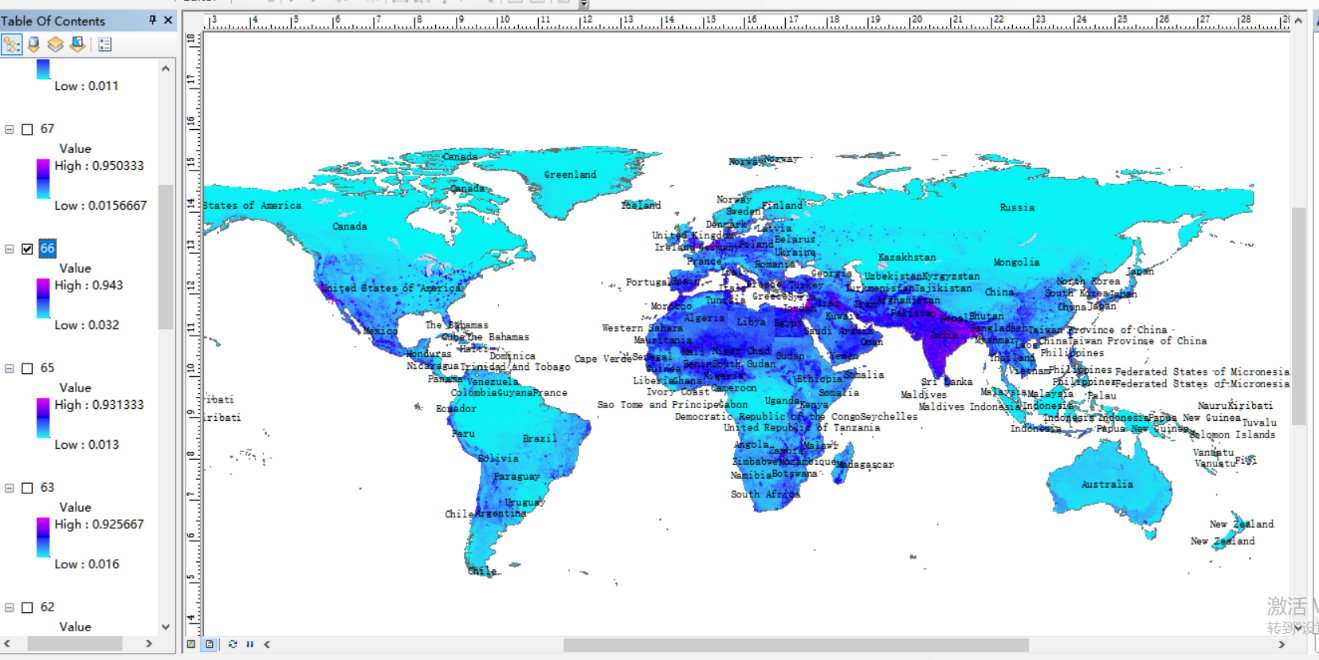


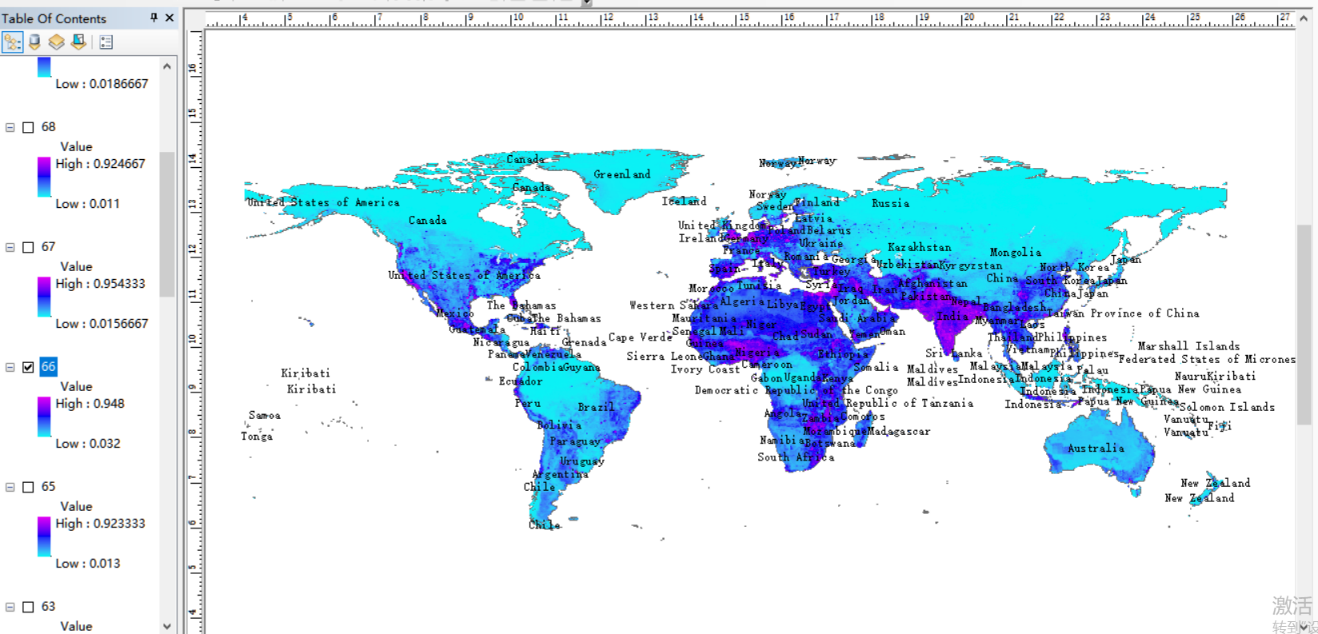


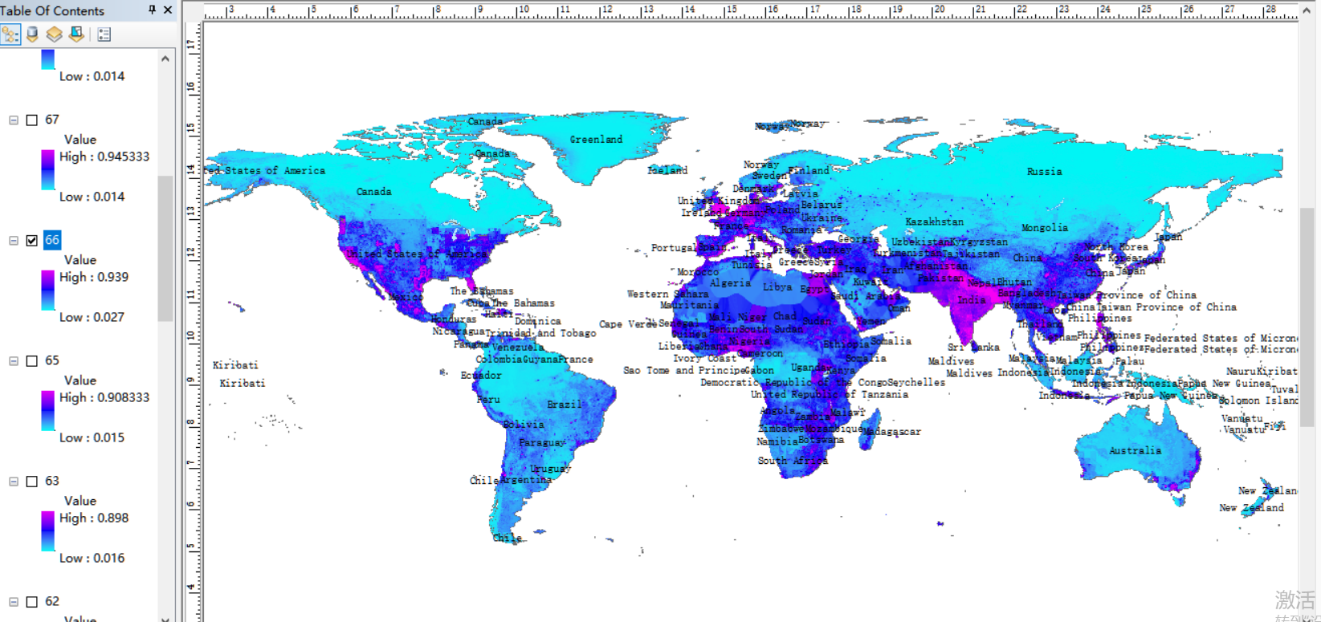


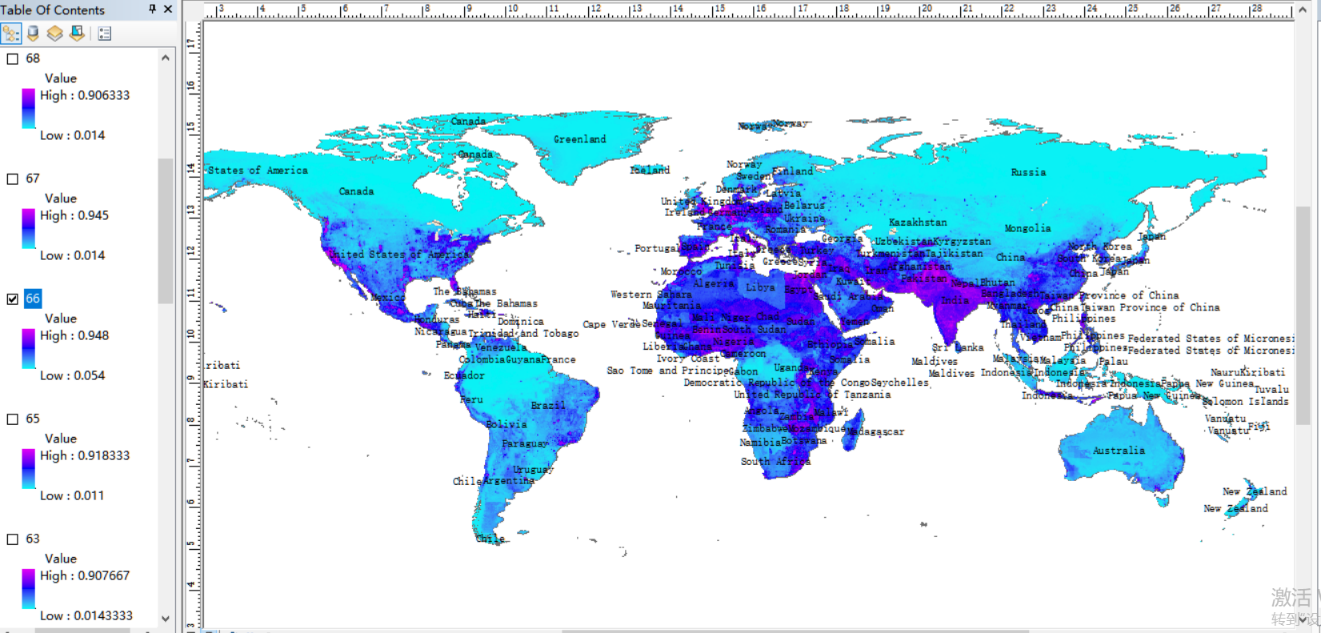


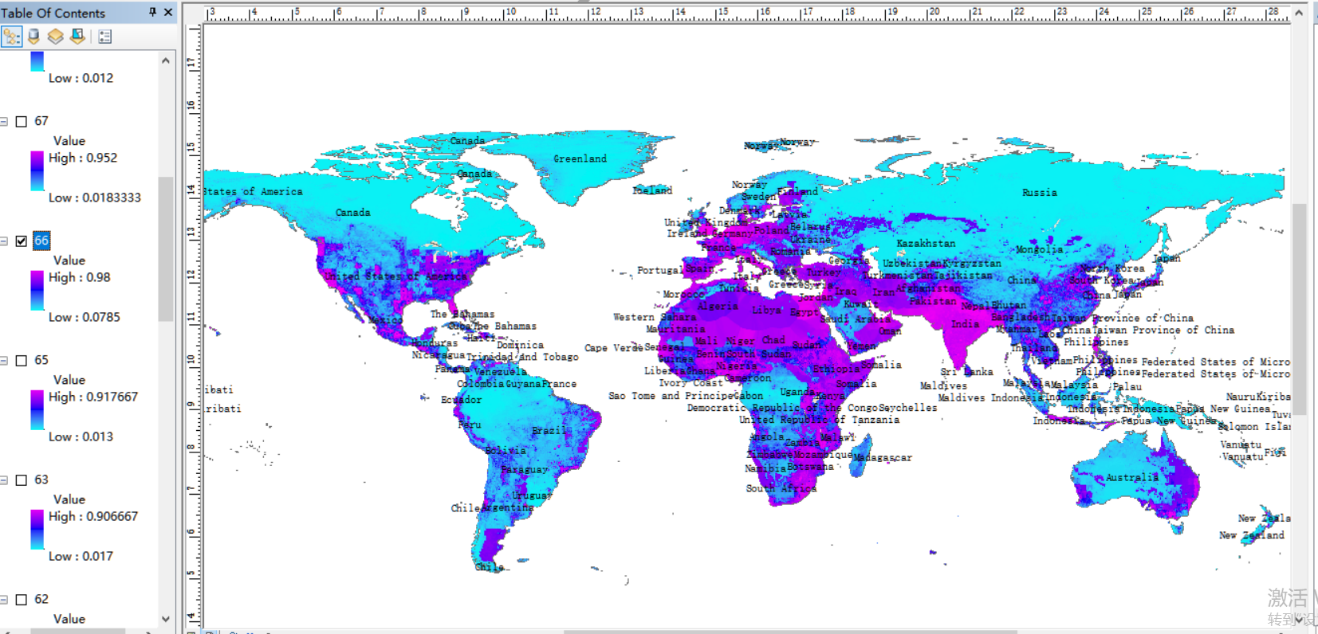


*Wasmannia auropunctata* Current, F126, F585, M126 and M585 in this order


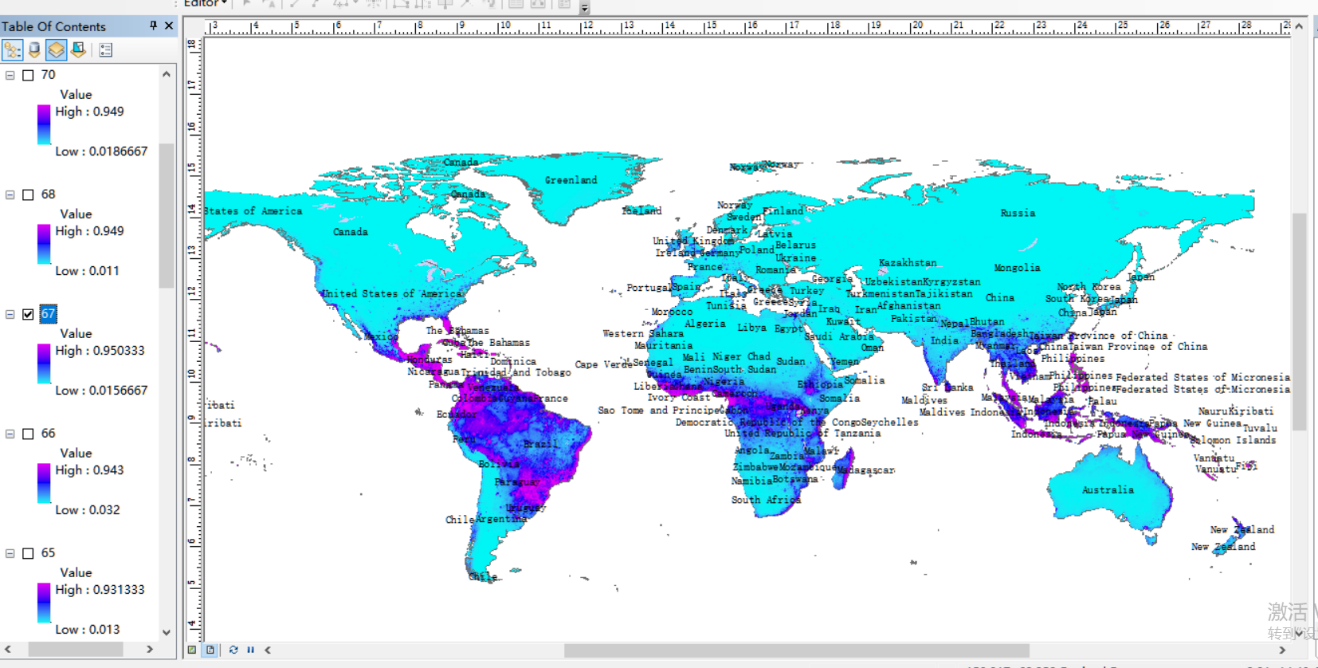


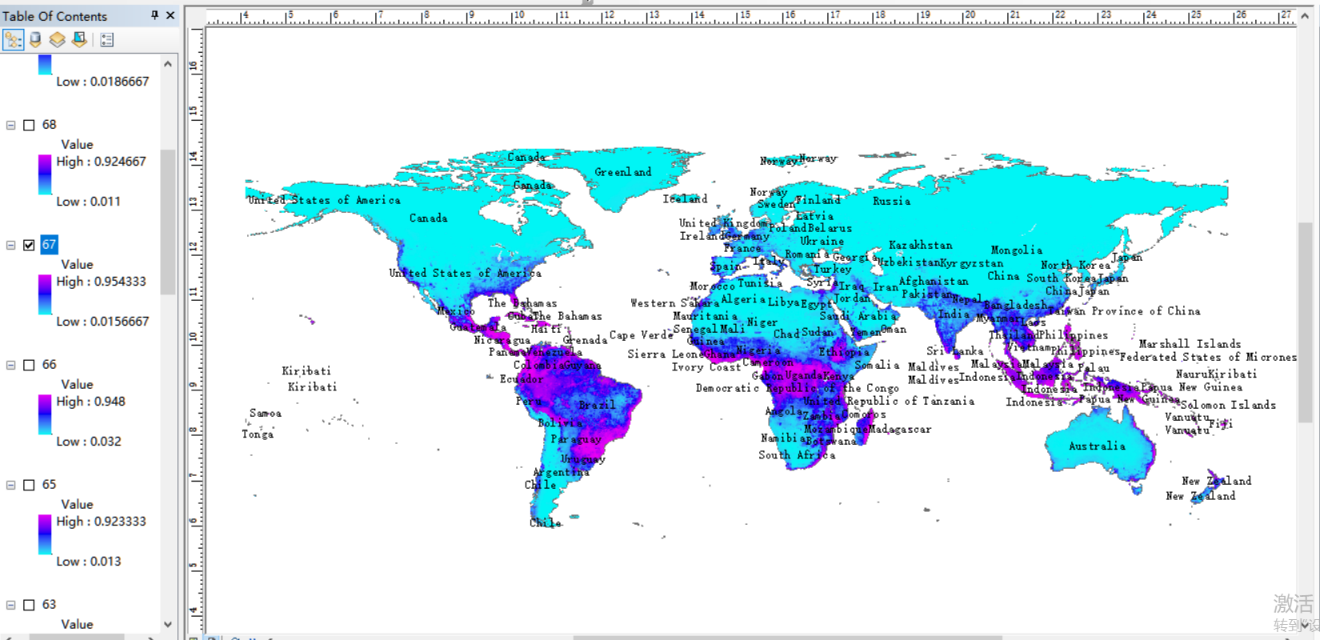


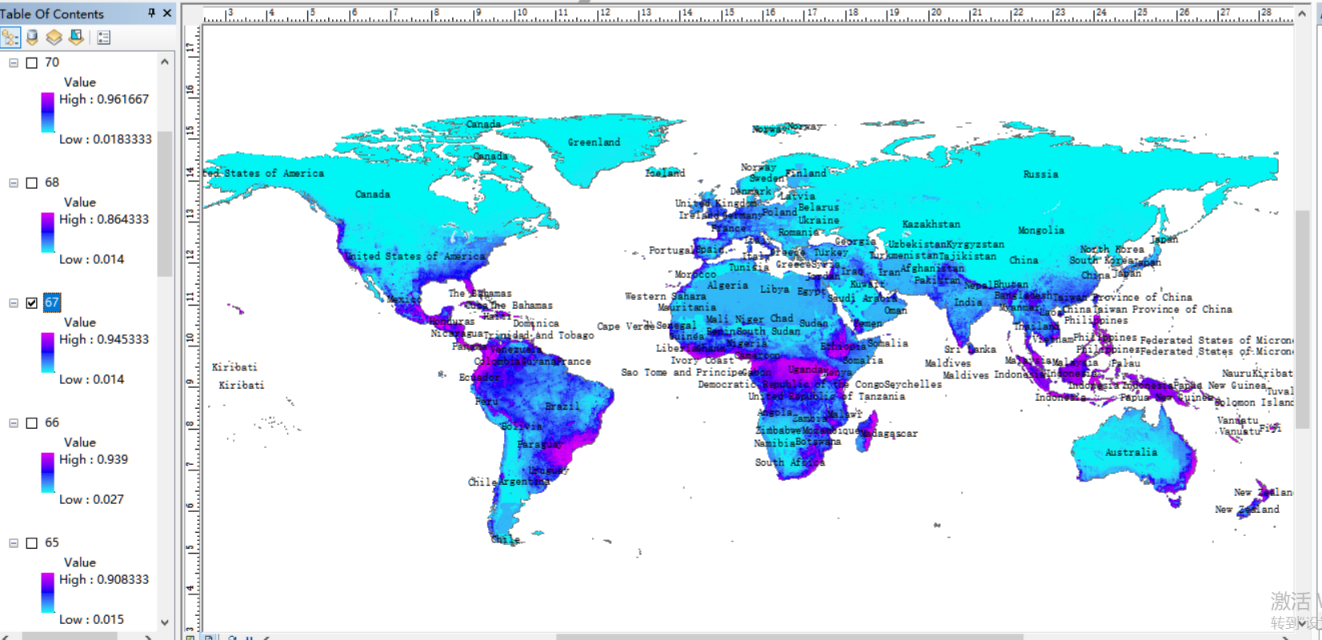


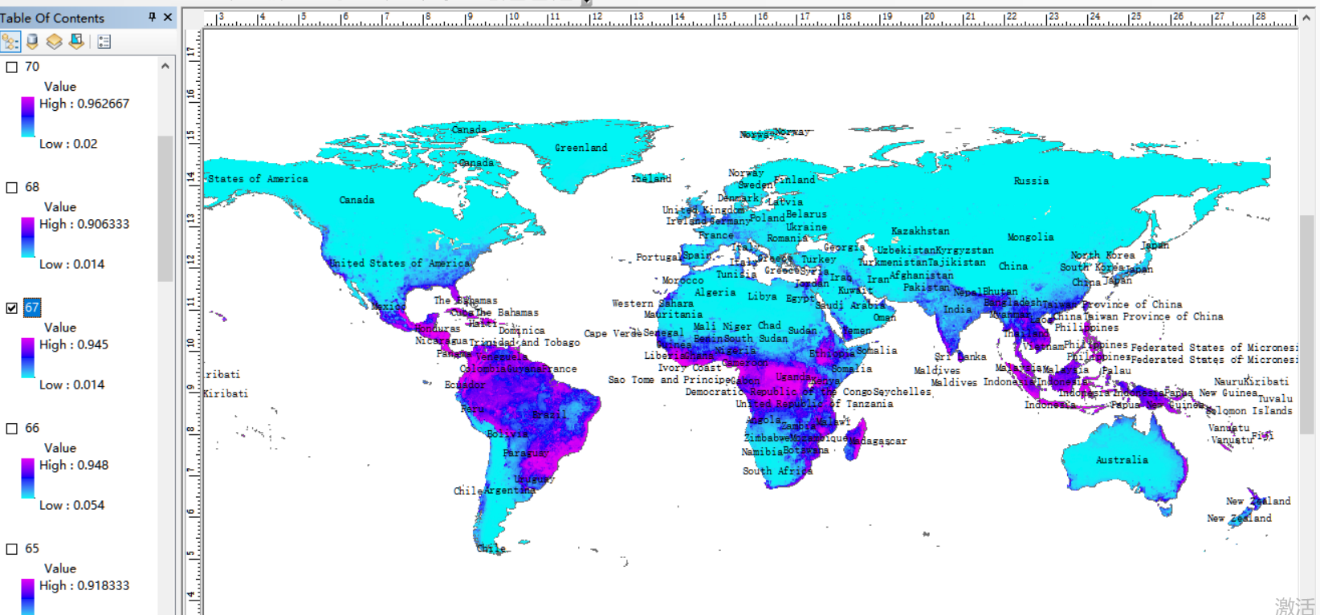


*
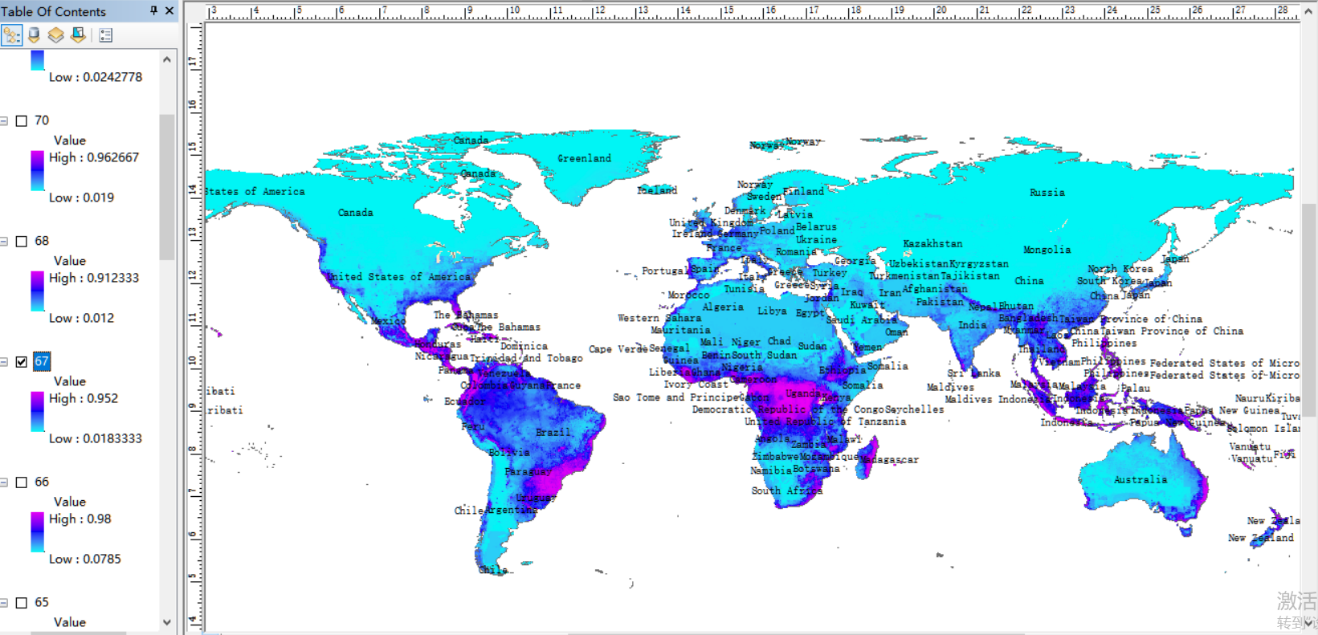
*

*Solenopsis invicta* Current, F126, F585, M126 and M585 in this order


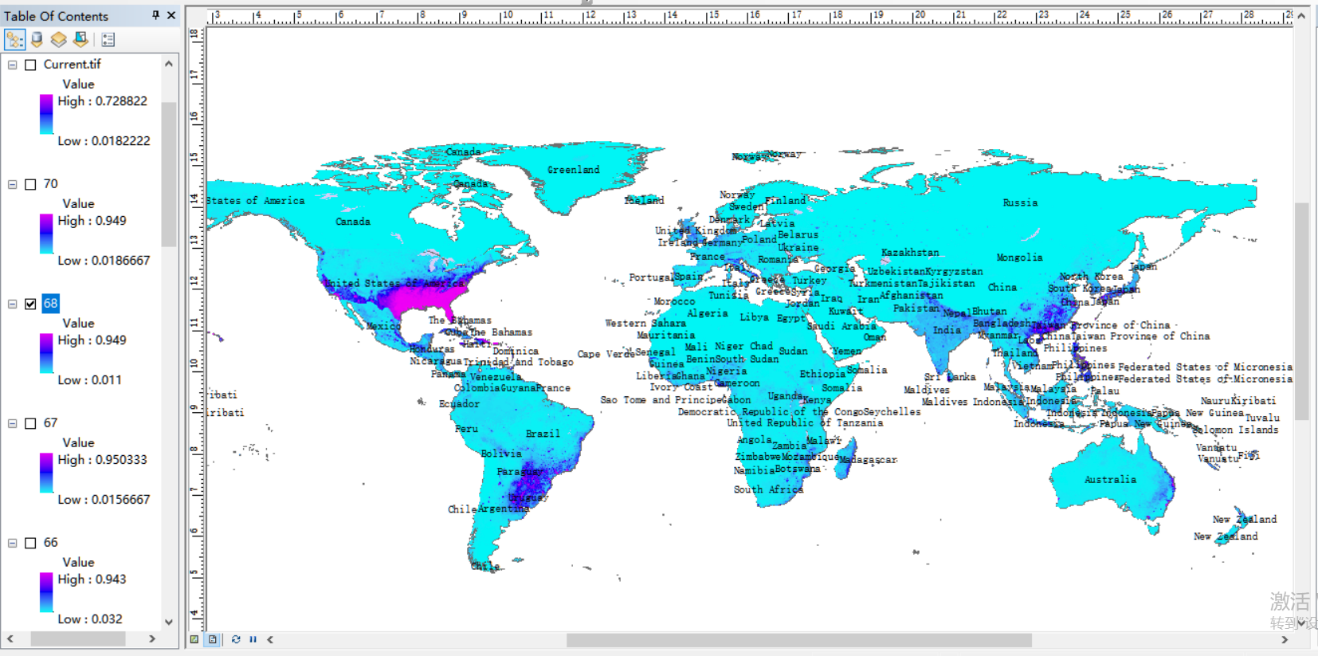


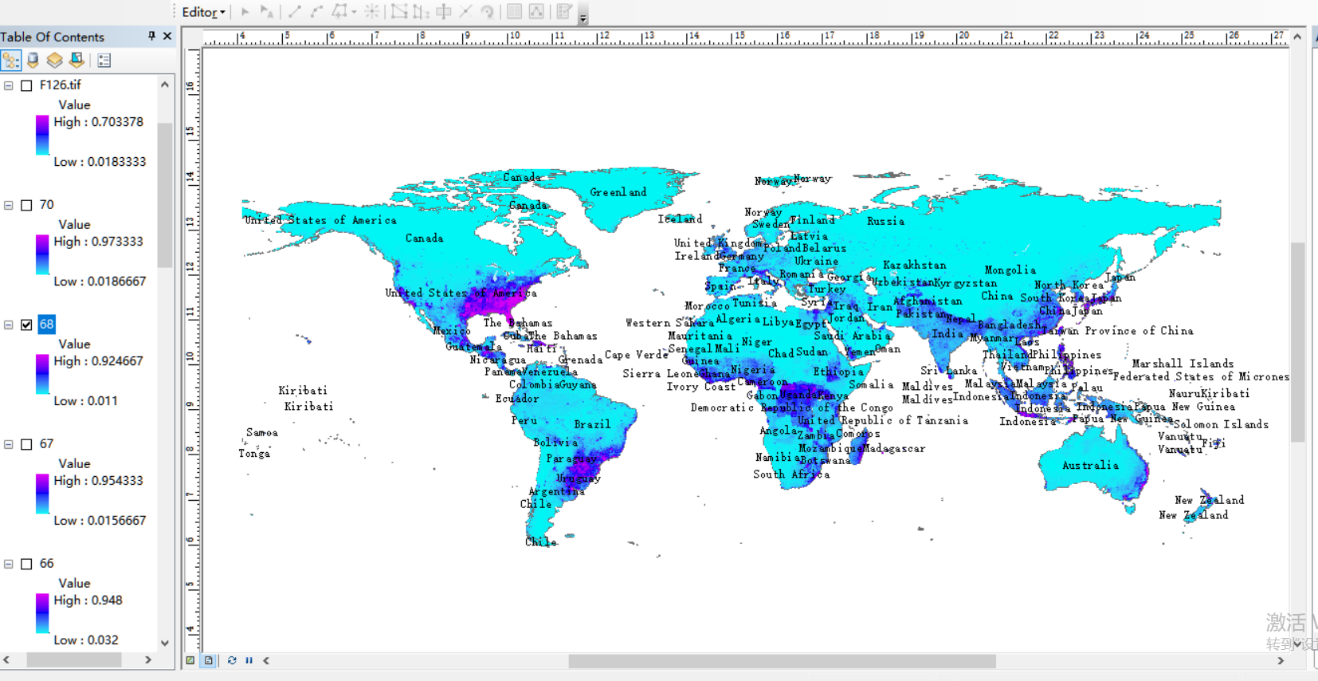


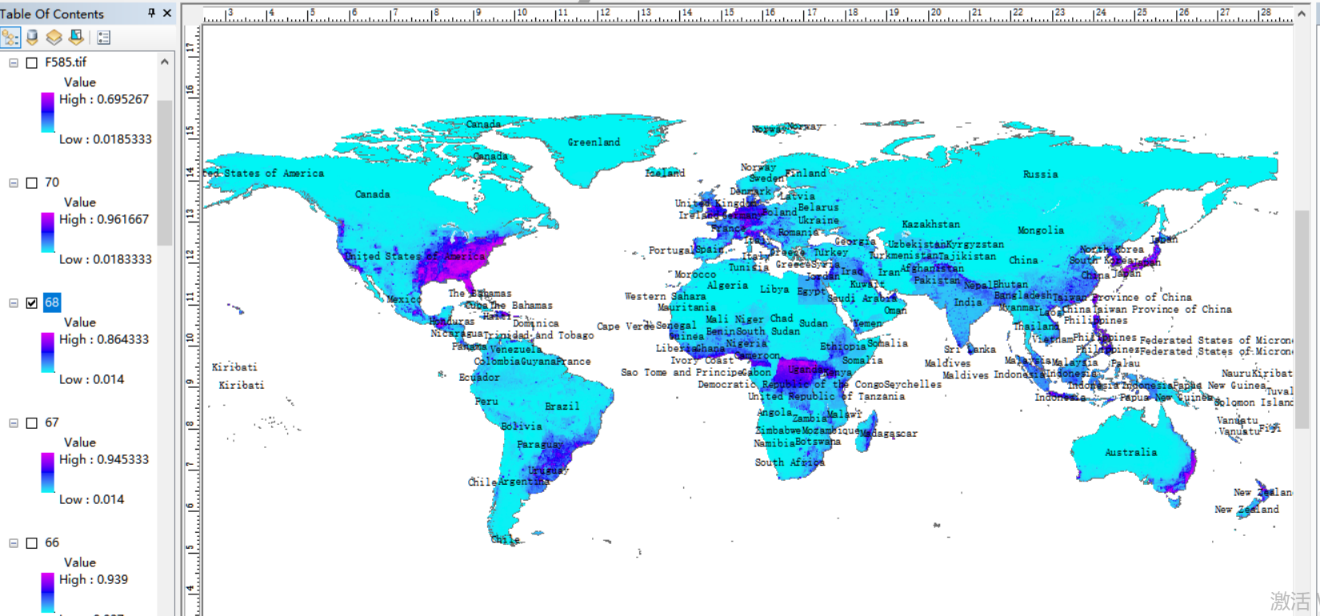


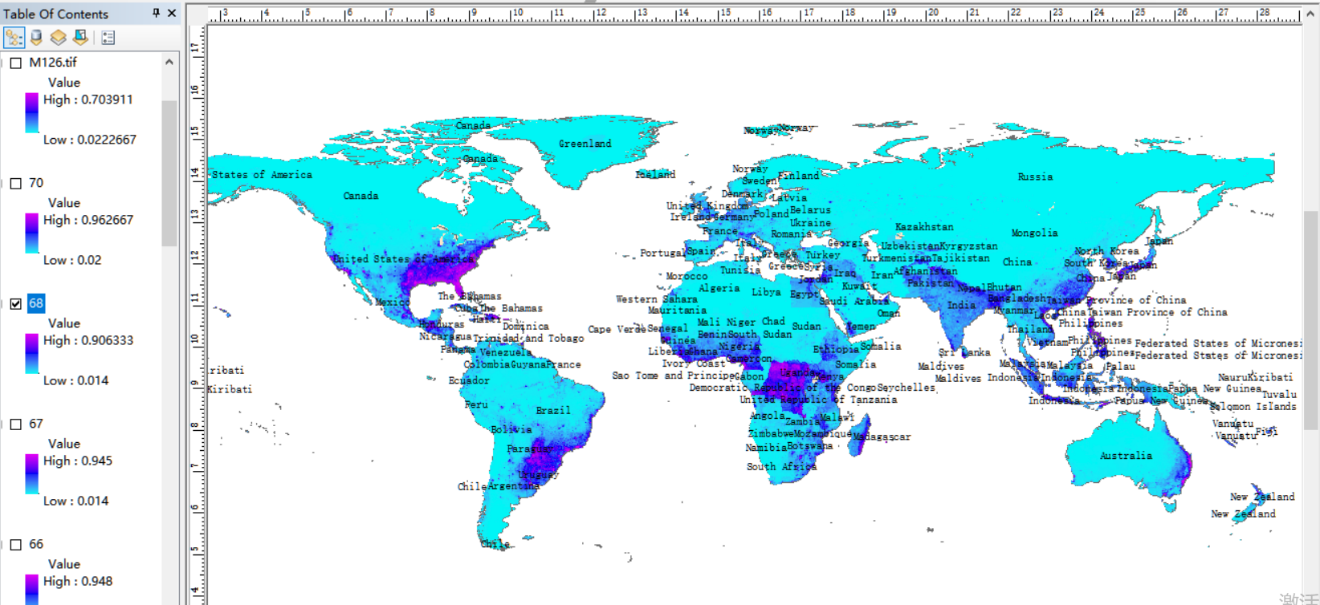


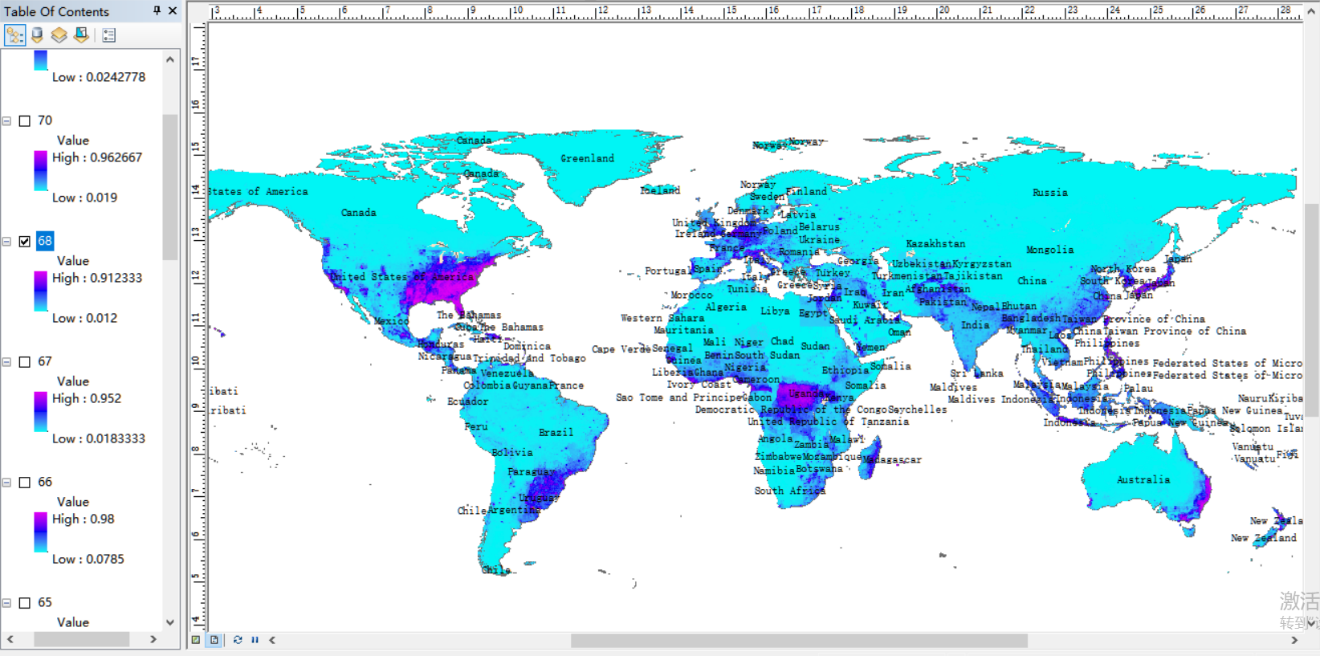


*Bemisia tabaci* Current, F126, F585, M126 and M585 in this order


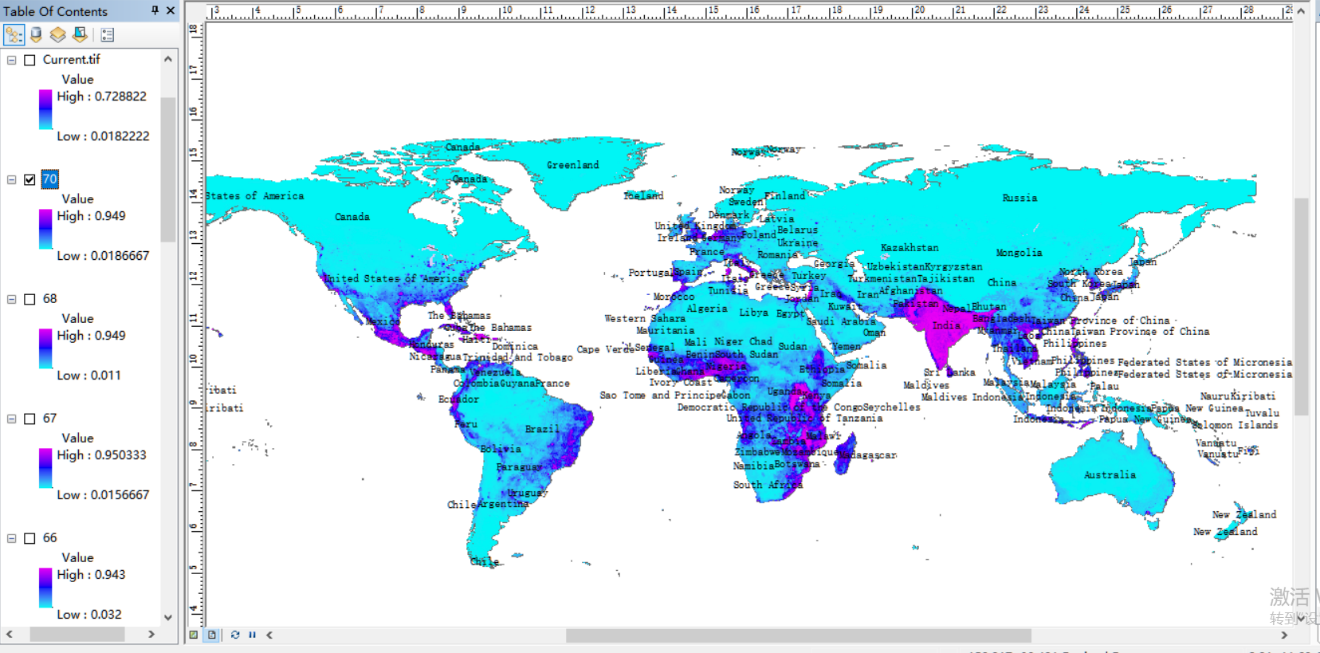


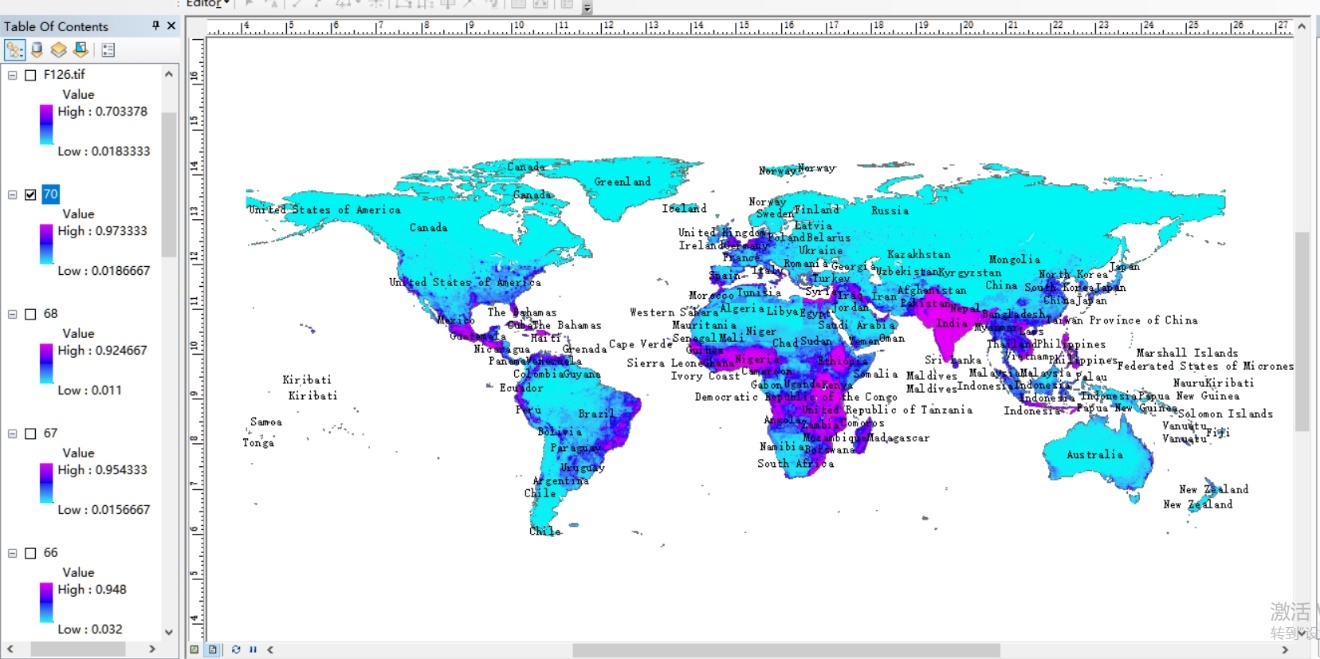


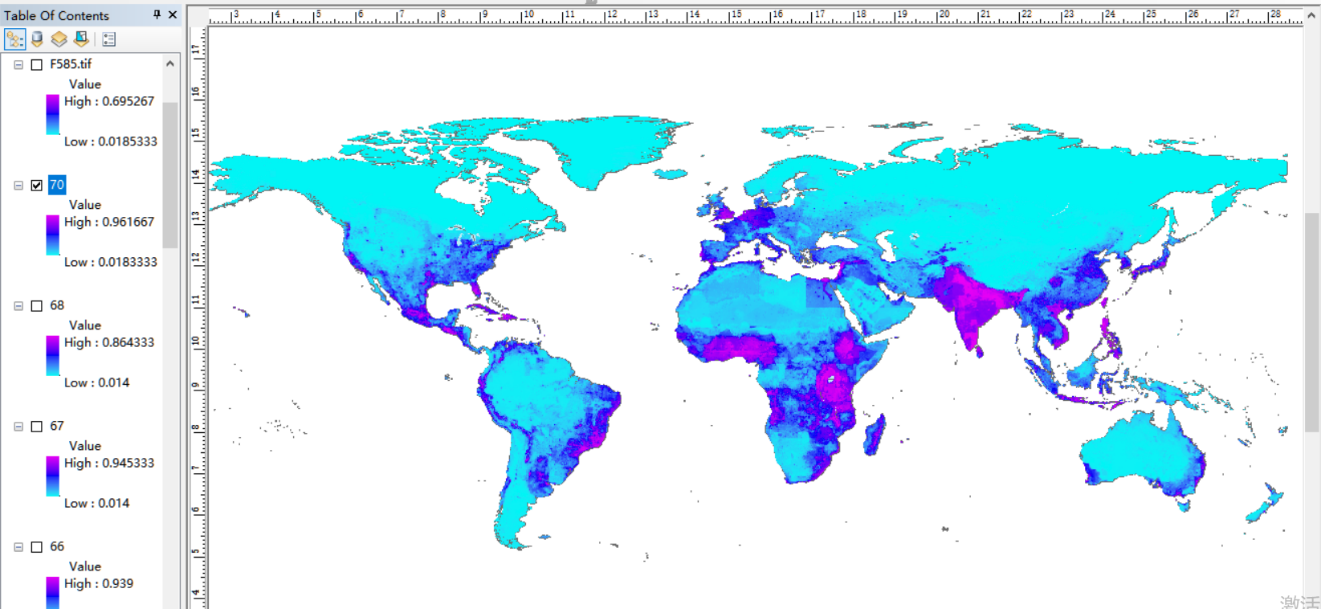


*
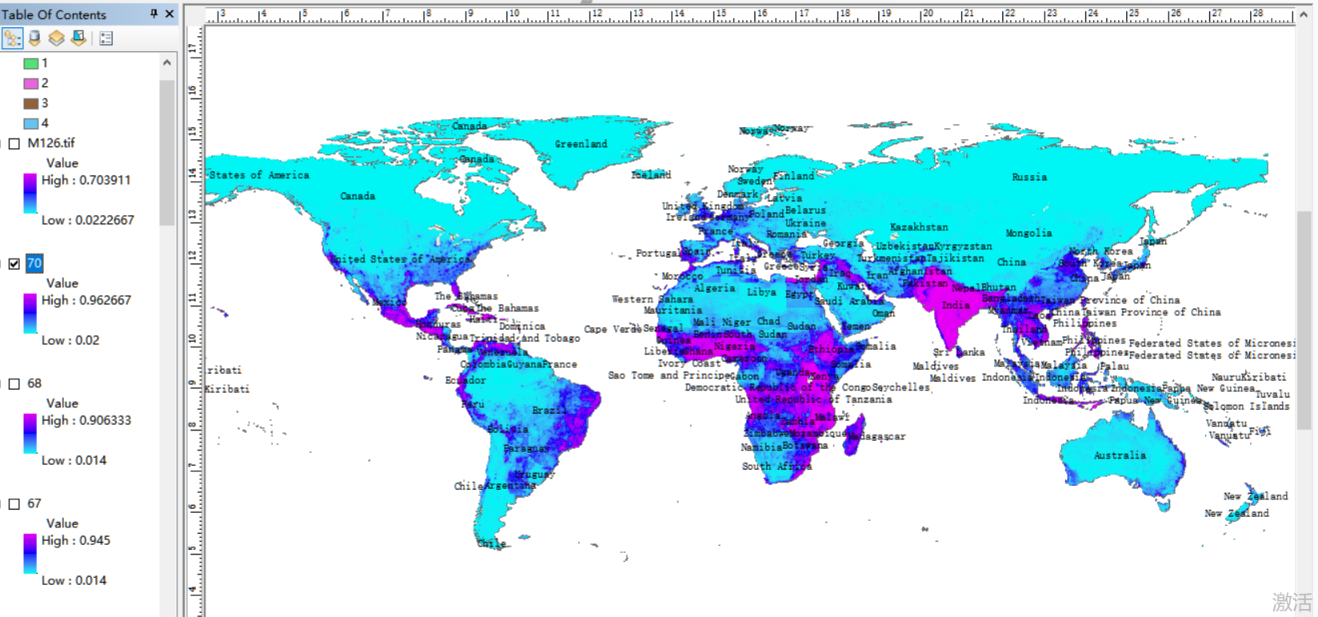
*


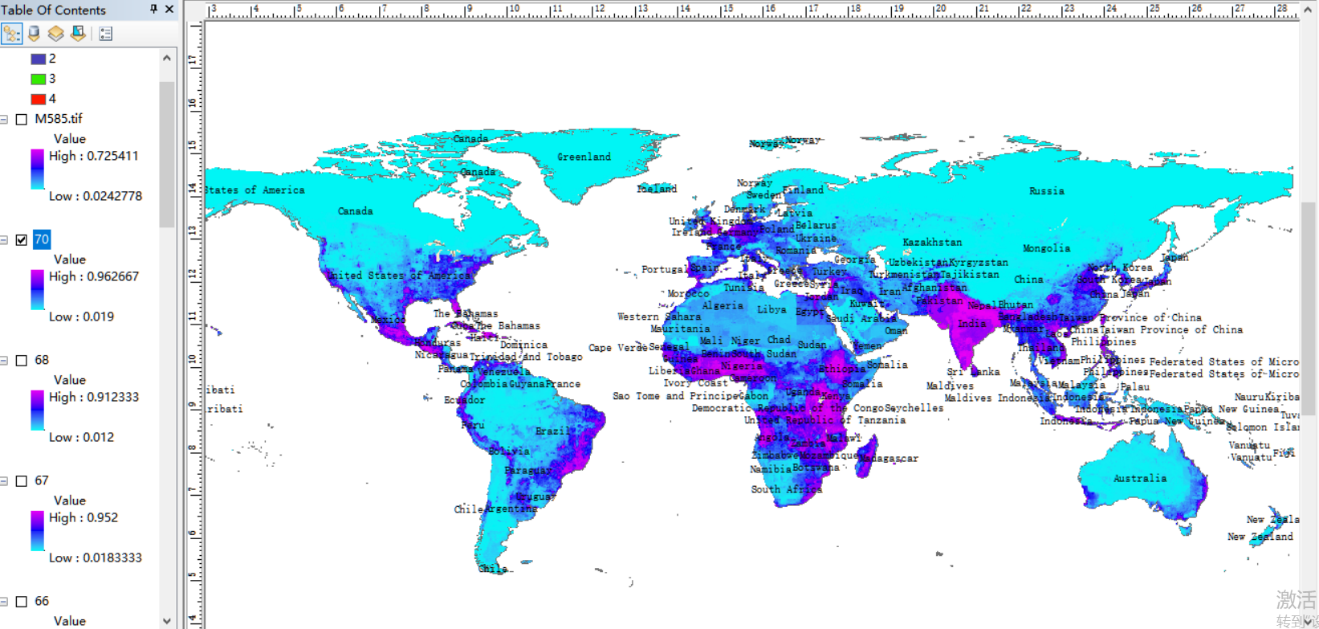

Supplement: Supplementary file 1 [file insects-15-00280-s001.zip › Supplemental Material S7.docx]
