# Supplementary material for: Future Climate Change and Anthropogenic Disturbance Promote the Invasions of the World’s Worst Invasive Insect Pests
Source: Insects. 2024 Apr 16;15(4):280. doi: 10.3390/insects15040280 (PMC11050065; doi:10.3390/insects15040280)
Supplement: Supplementary file 1 [file insects-15-00280-s001.zip › Supplemental Material S9.pdf]

S9 Potential ranges of 15 worst insect pest

Linepithema humile Current, F126, F585, M126 and M585 in this order

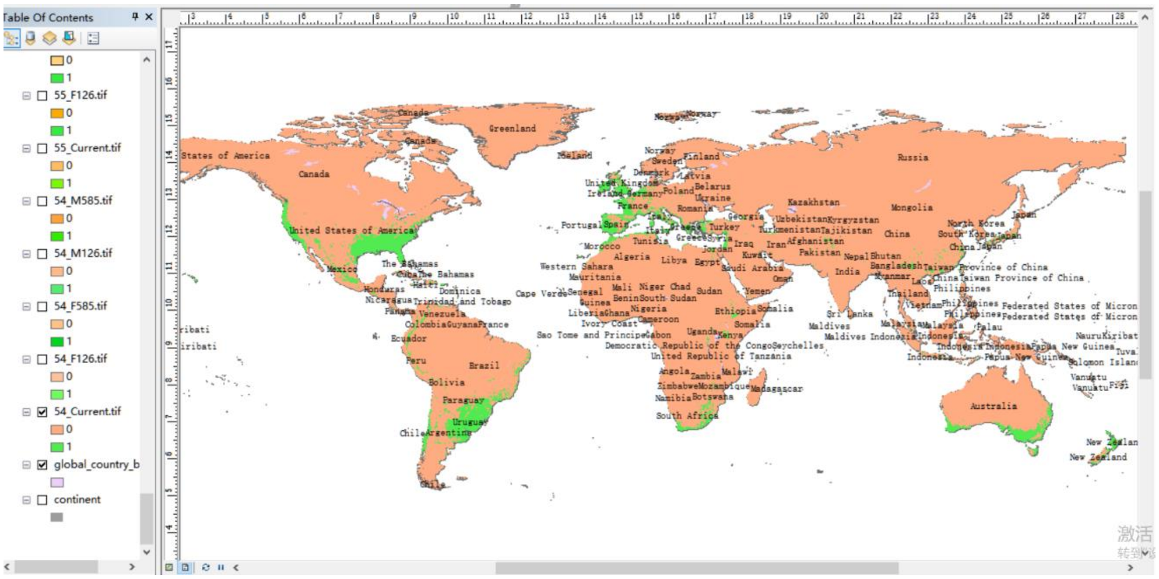

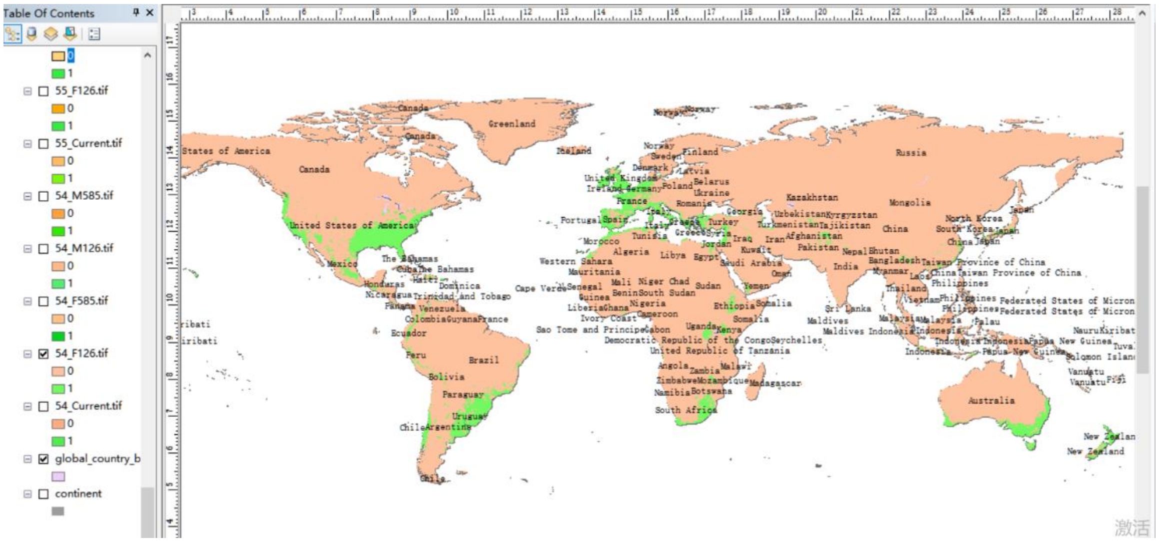

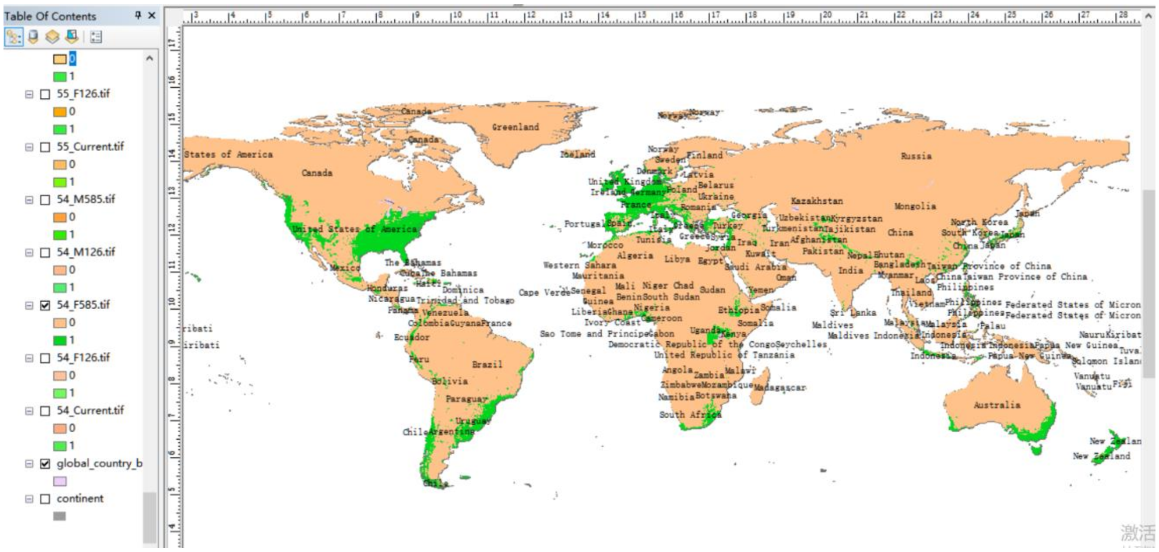
Table Of Contents
0
1
55_F126.tif
0
1
55_Current.tif
0
1
54_M585.tif
0
1
54_M126.tif
0
1
54_F585.tif
0
1
54_F126.tif
0
1
54_Current.tif
0
1
global_country_b
continent

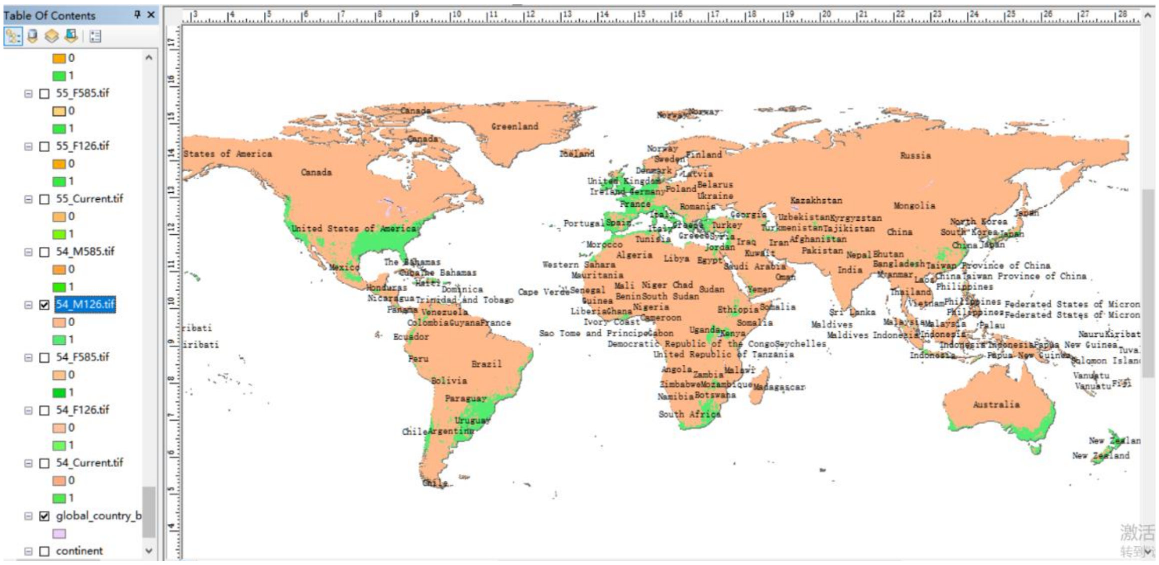
Table Of Contents
0
1
55_F585.tif
0
1
55_F126.tif
0
1
55_Current.tif
0
1
54_M585.tif
0
1
54_M126.tif
0
1
54_F585.tif
0
1
54_F126.tif
0
1
54_Current.tif
0
1
global_country_b
continent

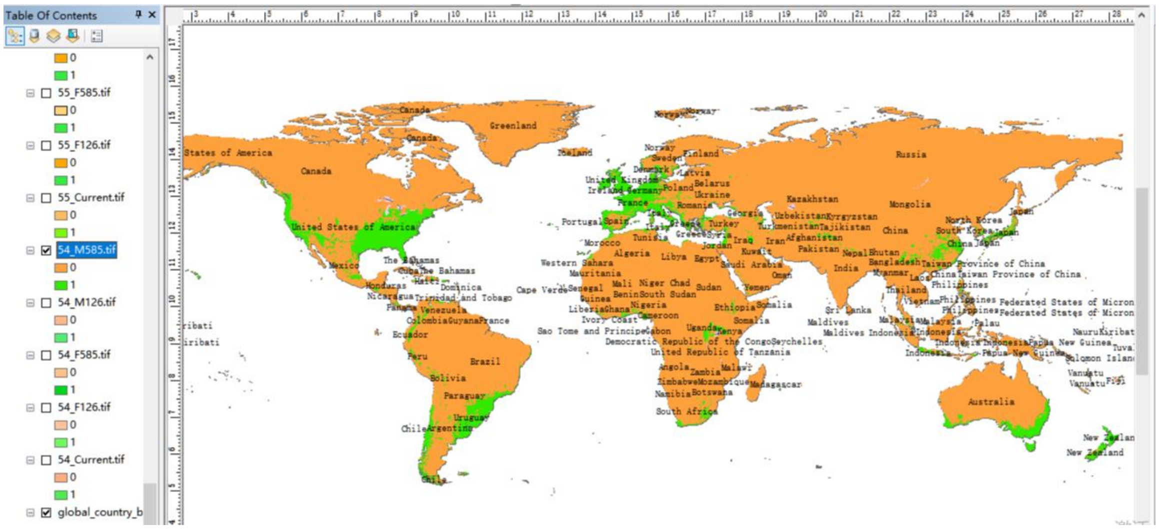
Table Of Contents
0
1
55_F585.tif
0
1
55_F126.tif
0
1
55_Current.tif
0
1
54_M585.tif
0
1
54_M126.tif
0
1
54_F585.tif
0
1
54_F126.tif
0
1
54_Current.tif
0
1
global_country_b

Anoplophora glabripennis Current, F126, F585, M126 and M585 in this order

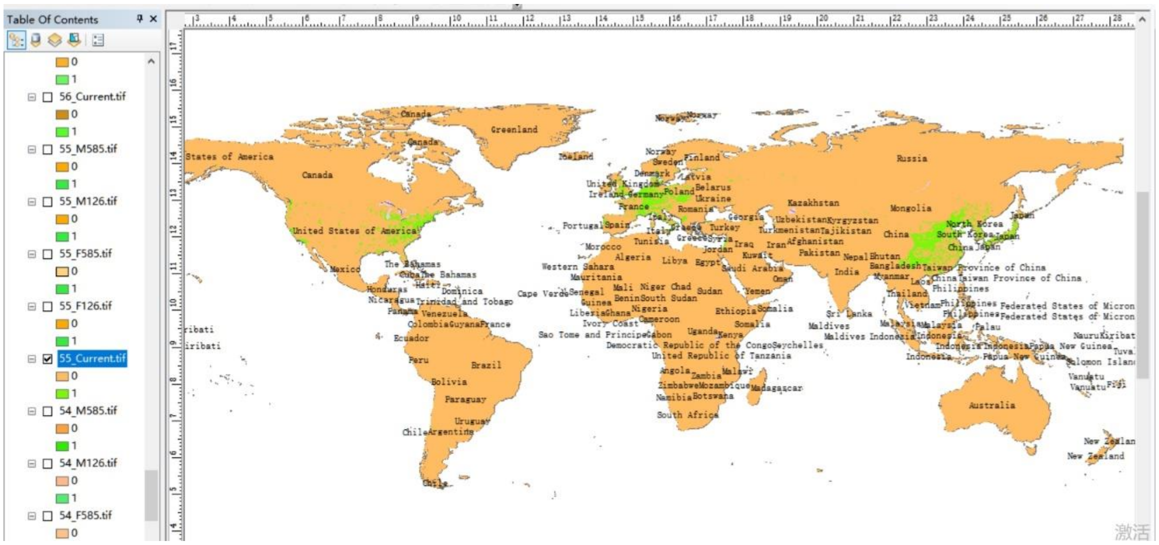

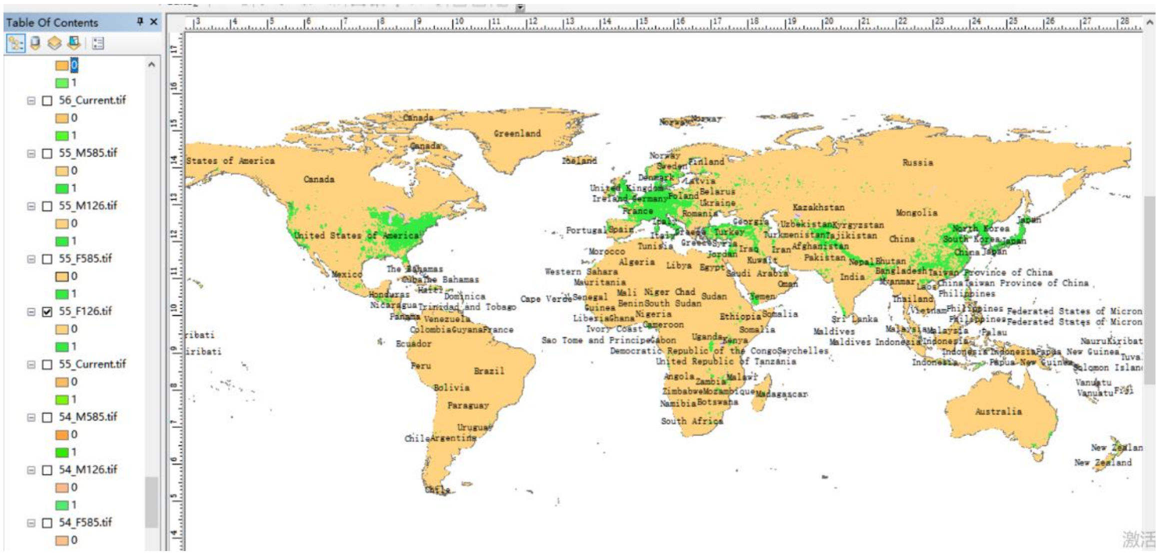

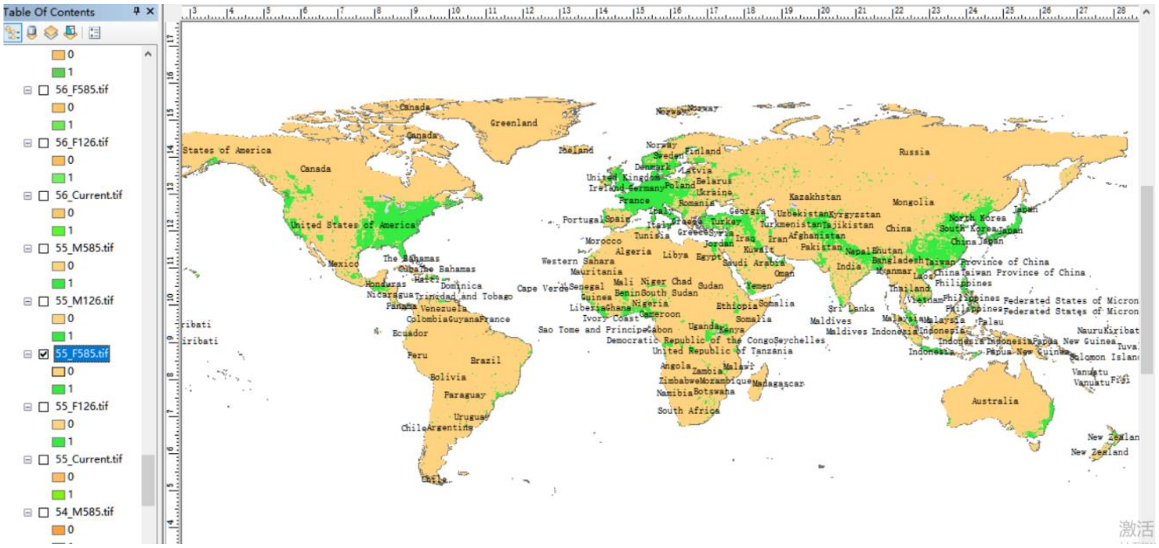
Table Of Contents
0
1
56_F585.tif
0
1
56_F126.tif
0
1
56_Current.tif
0
1
55_M585.tif
0
1
55_M126.tif
0
1
55_F585.tif
0
1
55_F126.tif
0
1
55_Current.tif
0
1
54_M585.tif
0

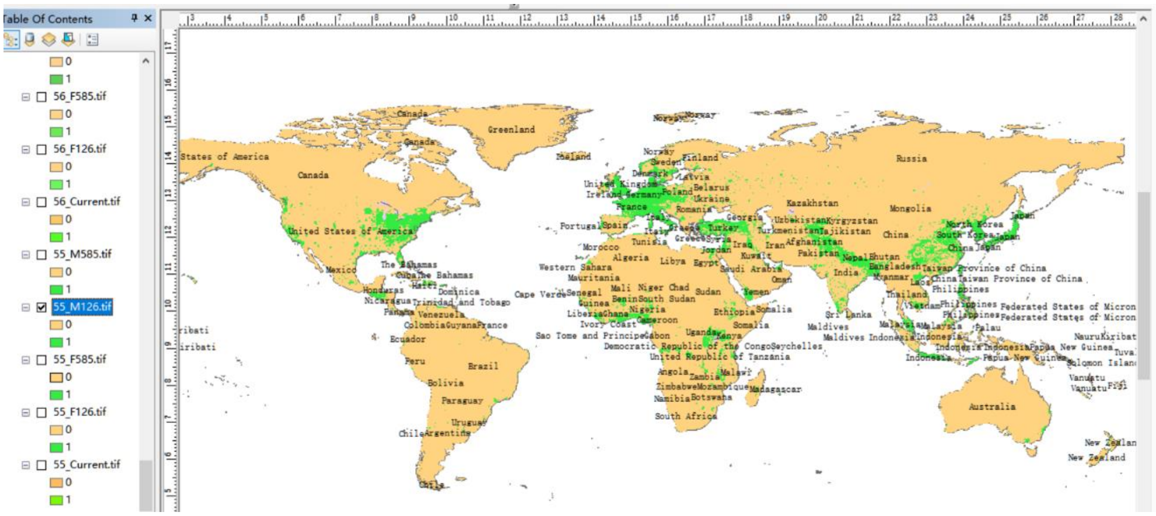
Table Of Contents
0
1
56_F585.tif
0
1
56_F126.tif
0
1
56_Current.tif
0
1
55_M585.tif
0
1
55_M126.tif
0
1
55_F585.tif
0
1
55_F126.tif
0
1
55_Current.tif
0
1

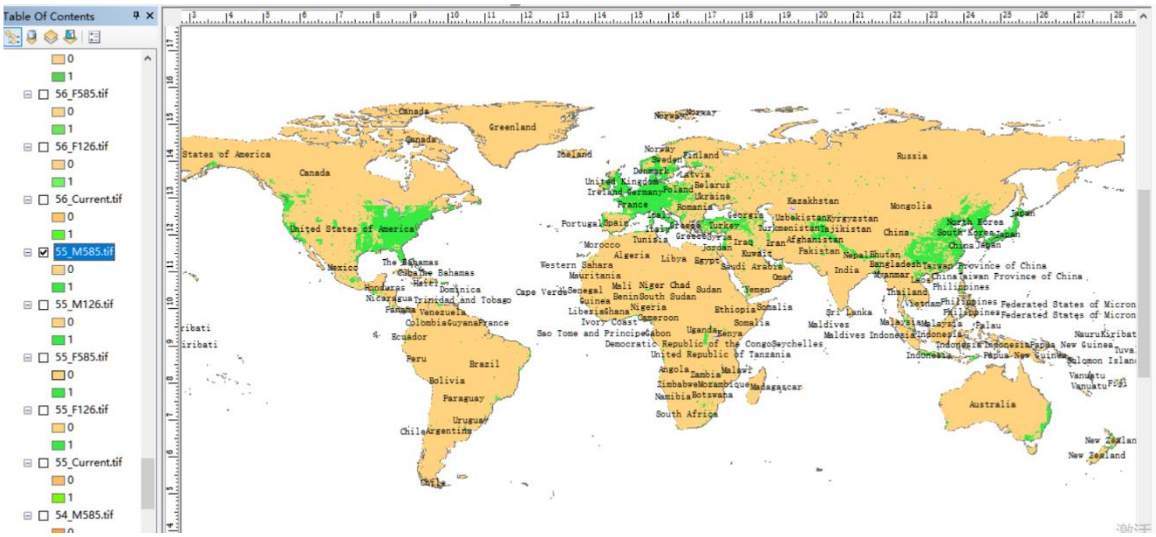
Table Of Contents
0
1
56_F585.tif
0
1
56_F126.tif
0
1
56_Current.tif
0
1
55_M585.tif
0
1
55_M126.tif
0
1
55_F585.tif
0
1
55_F126.tif
0
1
55_Current.tif
0
1
54_M585.tif

Aedes albopictus Current, F126, F585, M126 and M585 in this order

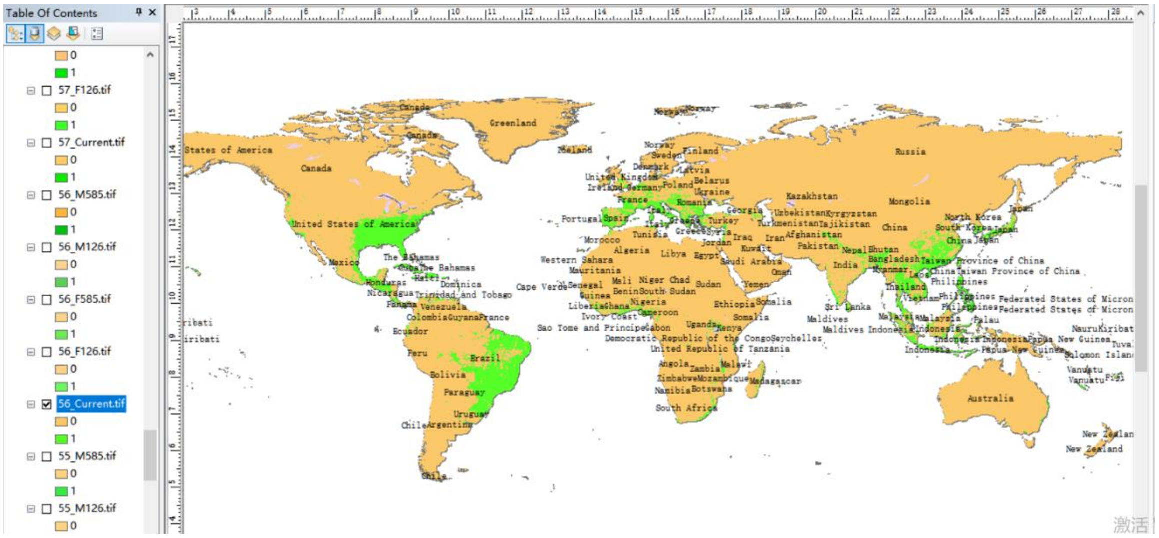

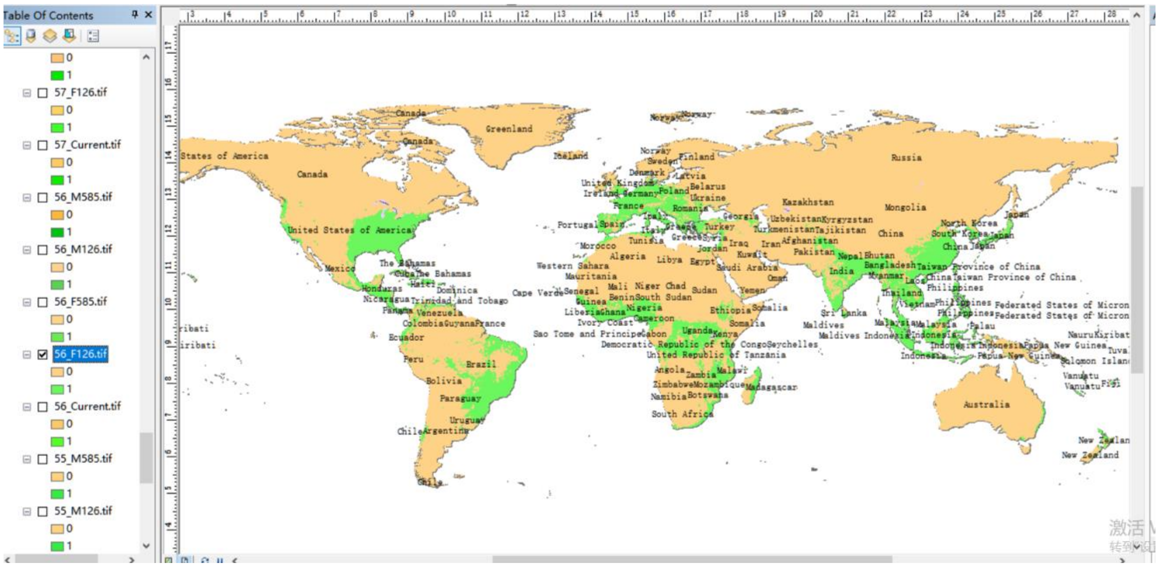

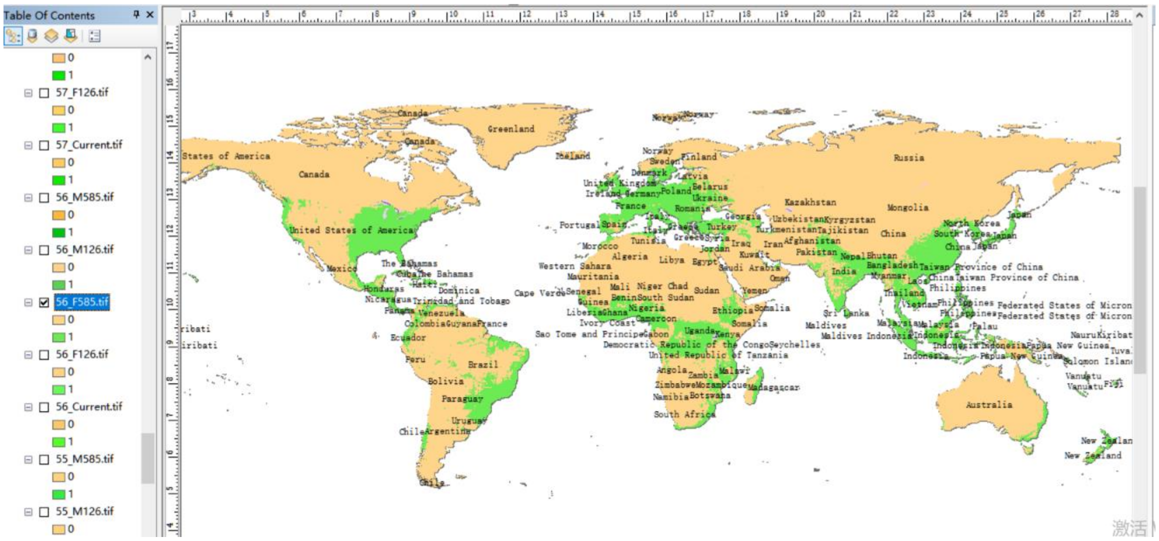
Table Of Contents
57_F126.tif
57_Current.tif
56_M585.tif
56_M126.tif
56_F585.tif
56_F126.tif
56_Current.tif
55_M585.tif
55_M126.tif

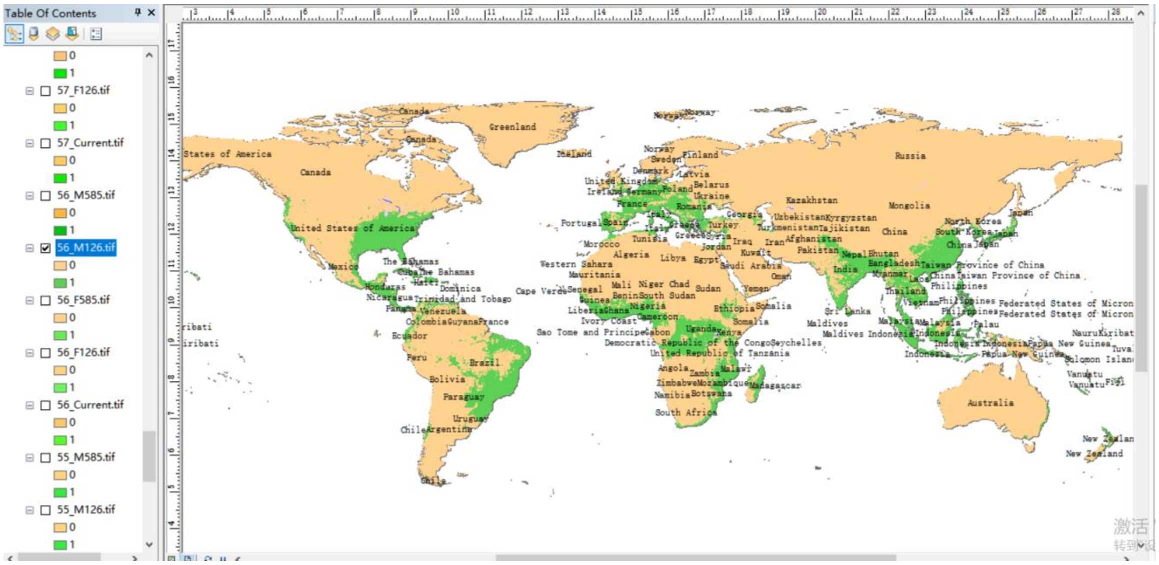
Table Of Contents
57_F126.tif
57_Current.tif
56_M585.tif
56_M126.tif
56_F585.tif
56_F126.tif
56_Current.tif
55_M585.tif
55_M126.tif

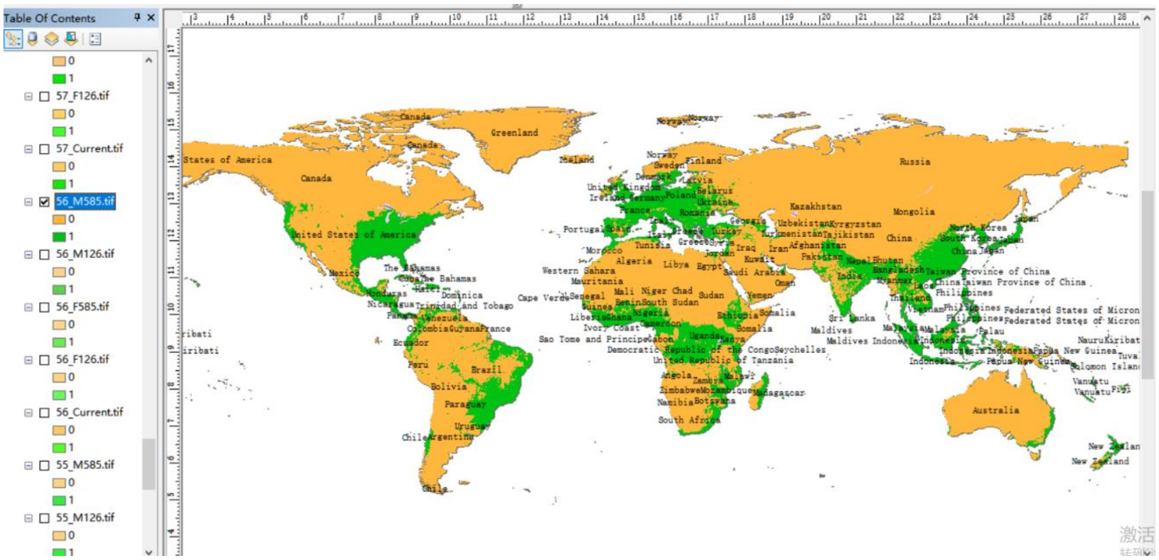
Table Of Contents
57_F126.tif
57_Current.tif
56_M585.tif
56_M126.tif
56_F585.tif
56_F126.tif
56_Current.tif
55_M585.tif
55_M126.tif

Pheidole megacephala Current, F126, F585, M126 and M585 in this order

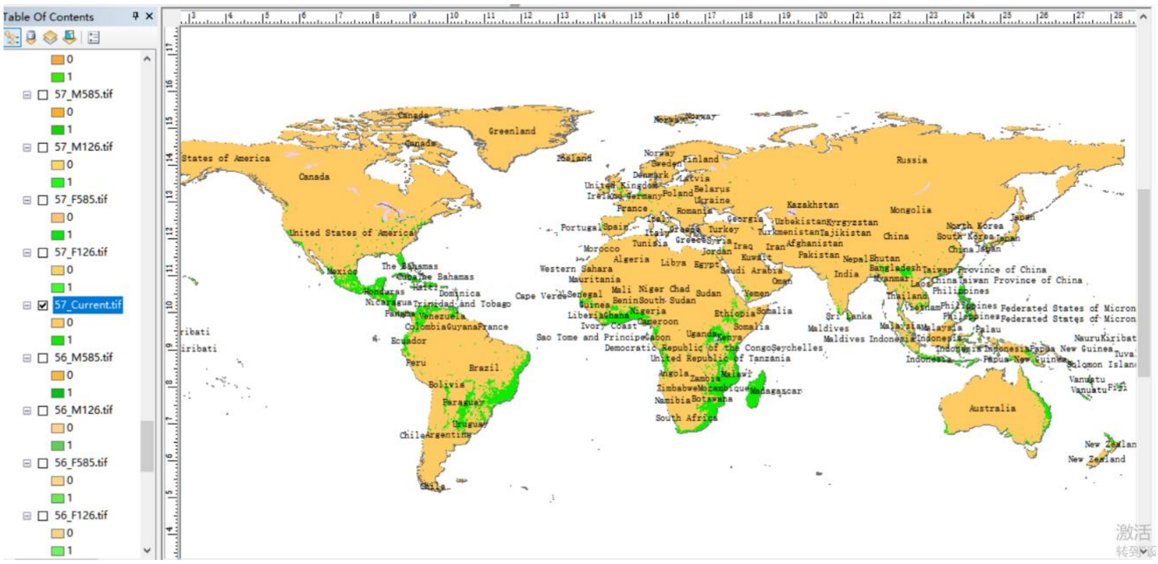

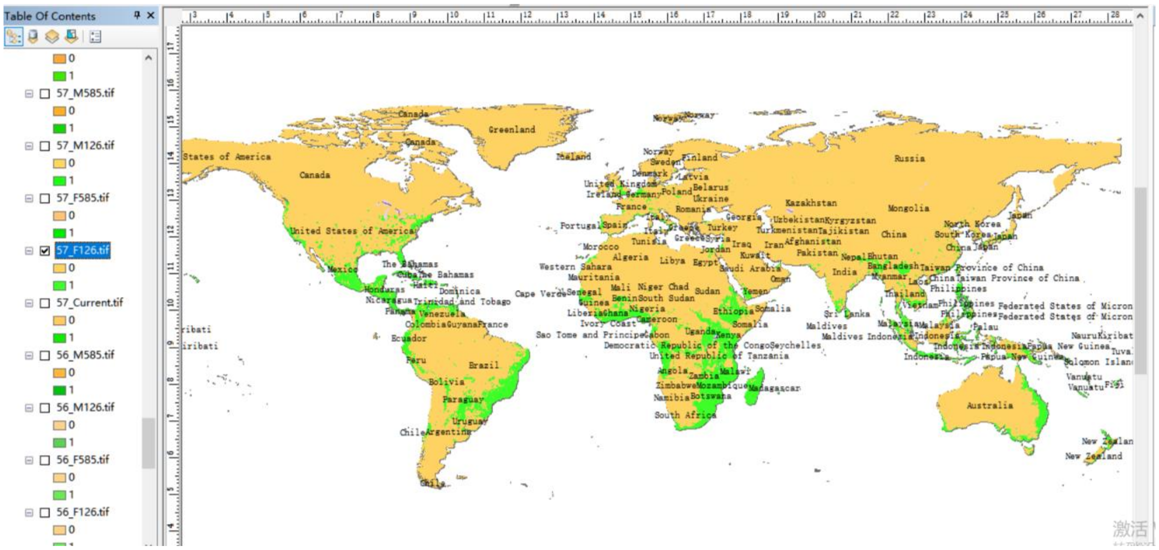

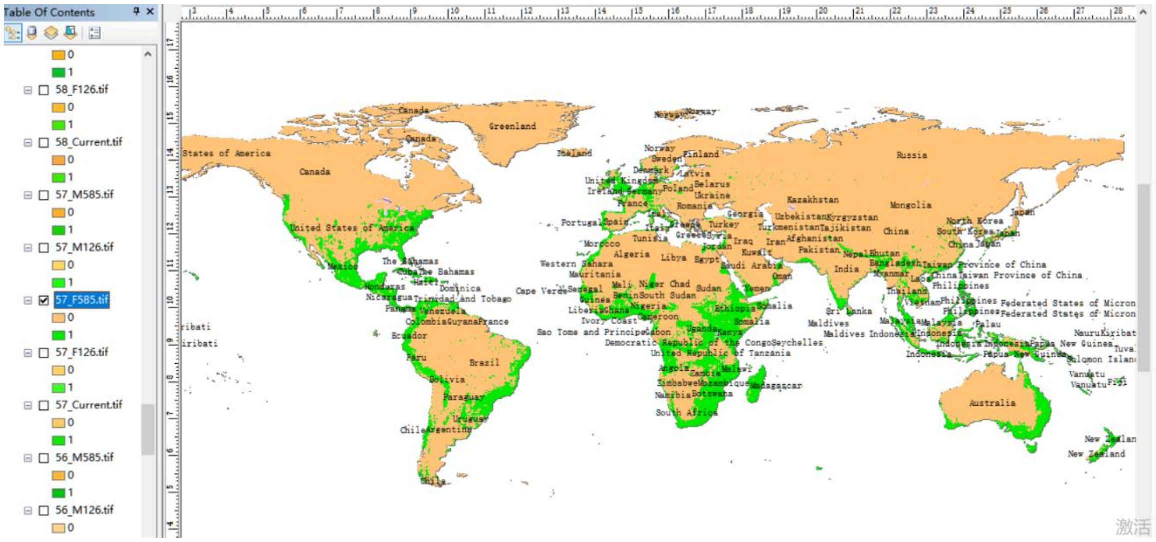
Table Of Contents
0
1
58_F126.tif
0
1
58_Current.tif
0
1
57_M585.tif
0
1
57_M126.tif
0
1
57_F585.tif
0
1
57_F126.tif
0
1
57_Current.tif
0
1
56_M585.tif
0
1
56_M126.tif
0
Greenland
Canada
Russia
United States of America
Brazil
Australia
Kazakhstan
Mongolia
China
India

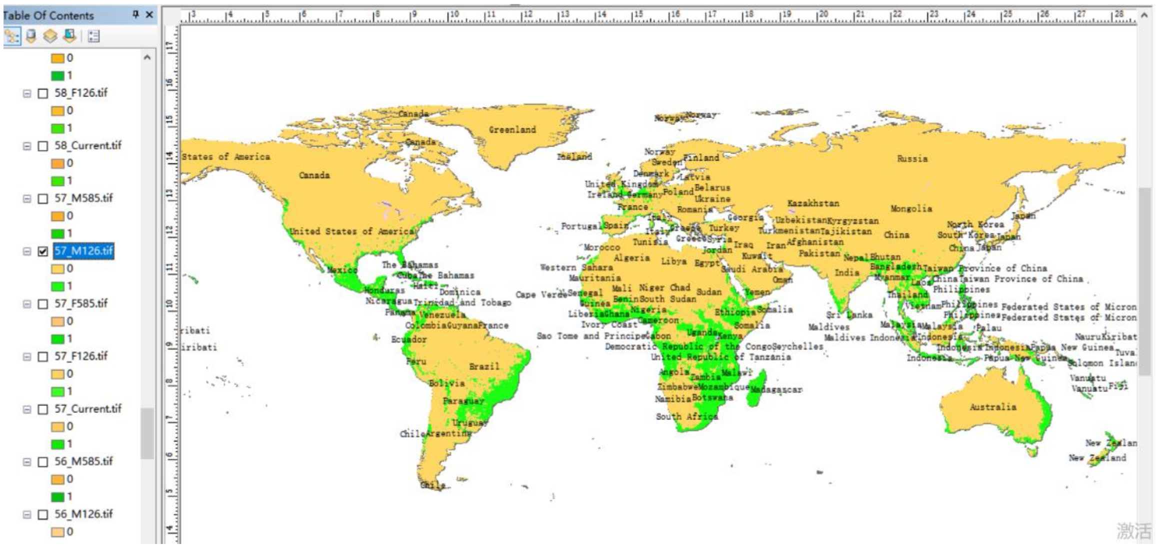
Table Of Contents
0
1
58_F126.tif
0
1
58_Current.tif
0
1
57_M585.tif
0
1
57_M126.tif
0
1
57_F585.tif
0
1
57_F126.tif
0
1
57_Current.tif
0
1
56_M585.tif
0
1
56_M126.tif
0
Greenland
Canada
Russia
United States of America
Brazil
Australia
Kazakhstan
Mongolia
China
India

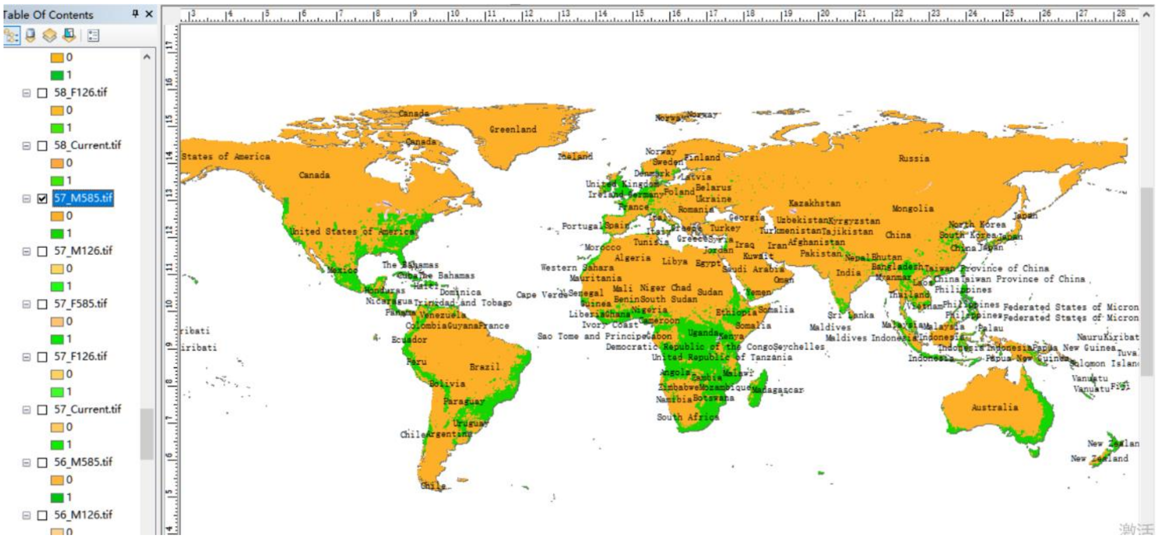
Table Of Contents
0
1
58_F126.tif
0
1
58_Current.tif
0
1
57_M585.tif
0
1
57_M126.tif
0
1
57_F585.tif
0
1
57_F126.tif
0
1
57_Current.tif
0
1
56_M585.tif
0
1
56_M126.tif
0
Greenland
Canada
Russia
United States of America
Brazil
Australia
Kazakhstan
Mongolia
China
India

Anopheles quadrimaculatus Current, F126, F585, M126 and M585 in this order

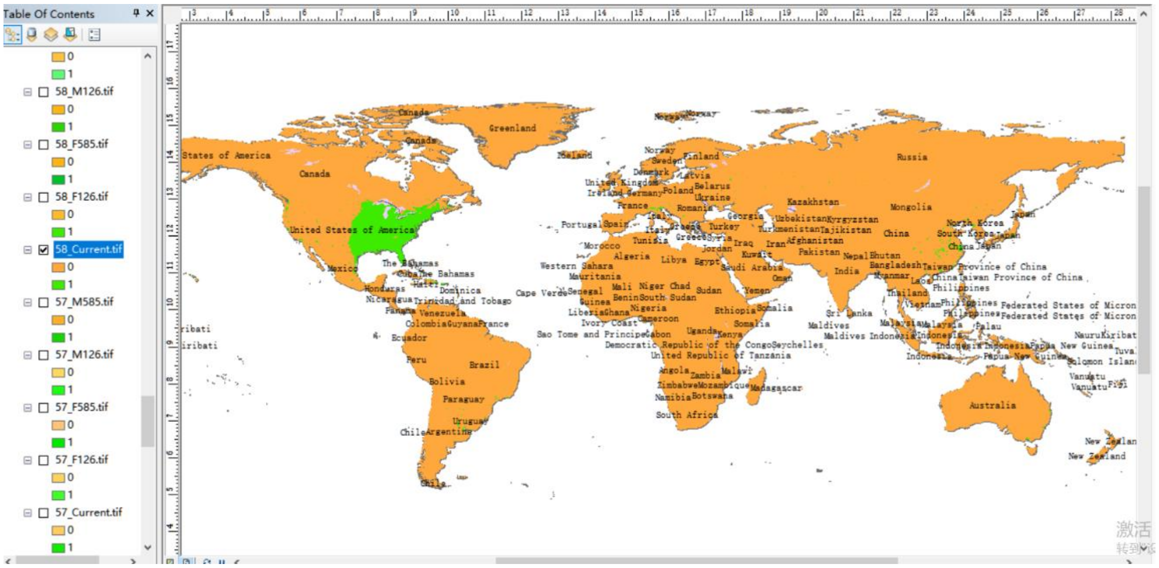

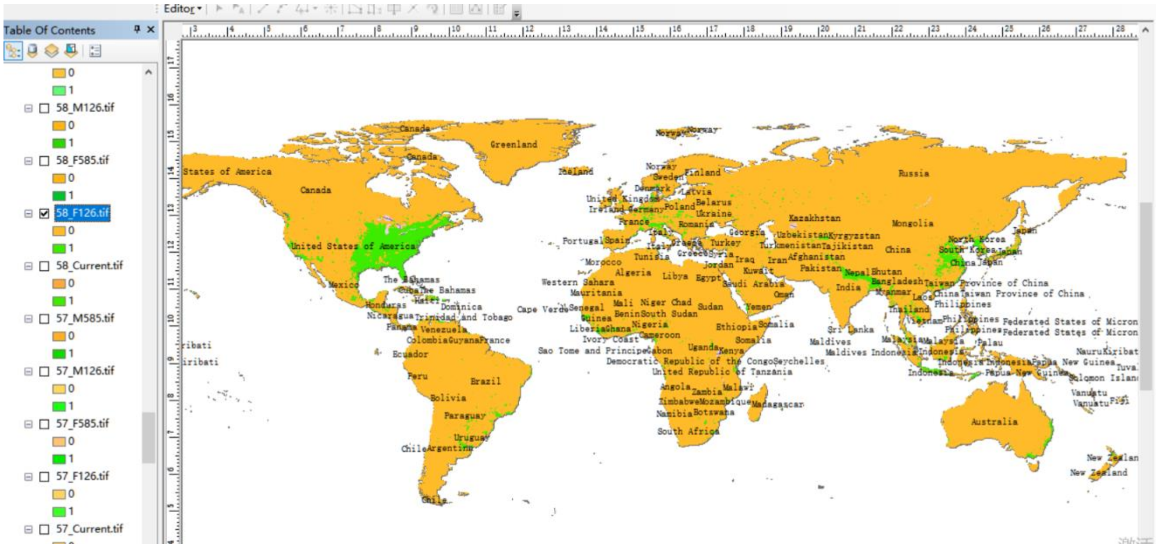

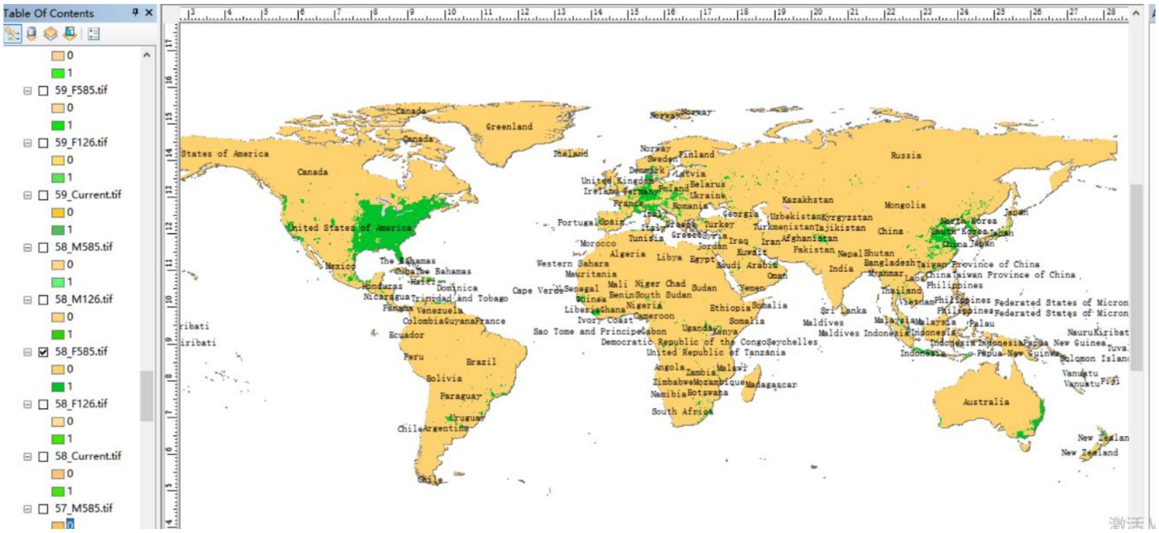
Table Of Contents
59_F585.tif
59_F126.tif
59_Current.tif
58_M585.tif
58_M126.tif
58_F585.tif
58_F126.tif
58_Current.tif
57_M585.tif

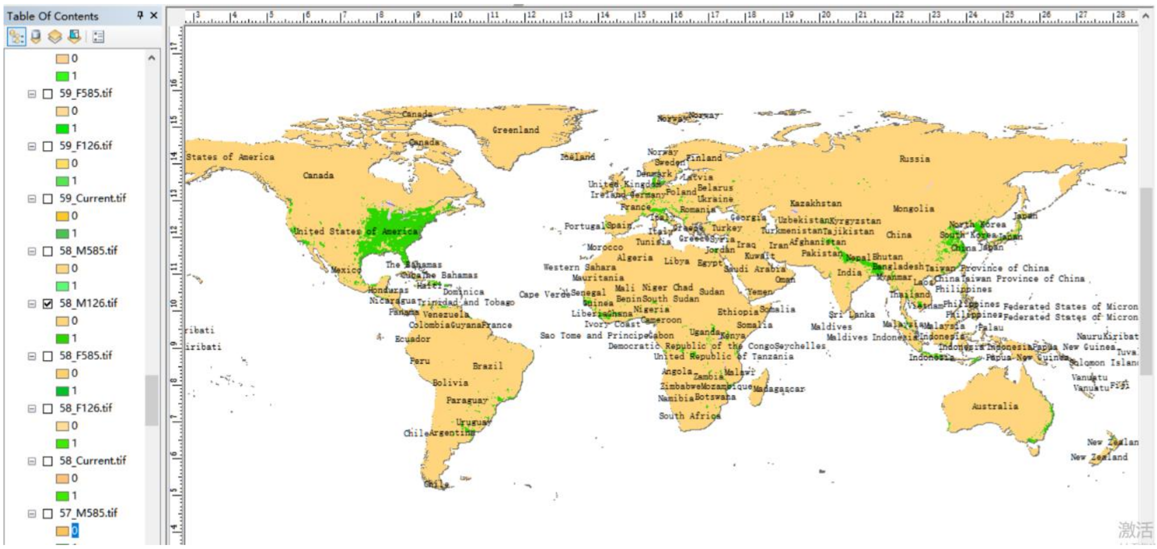
Table Of Contents
59_F585.tif
59_F126.tif
59_Current.tif
58_M585.tif
58_M126.tif
58_F585.tif
58_F126.tif
58_Current.tif
57_M585.tif

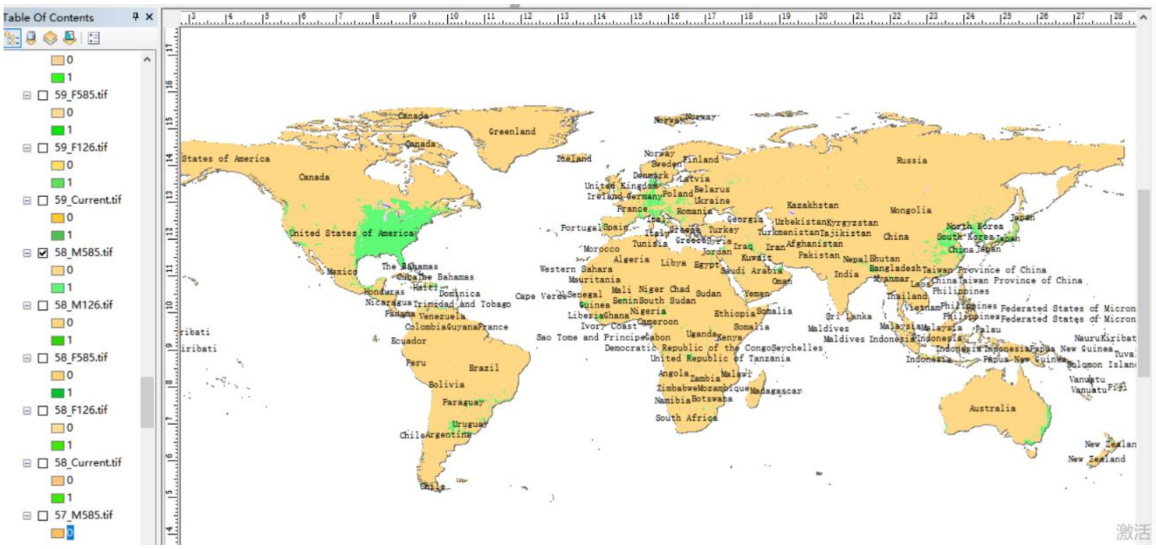
Table Of Contents
59_F585.tif
59_F126.tif
59_Current.tif
58_M585.tif
58_M126.tif
58_F585.tif
58_F126.tif
58_Current.tif
57_M585.tif

Vespula vulgaris Current, F126, F585, M126 and M585 in this order

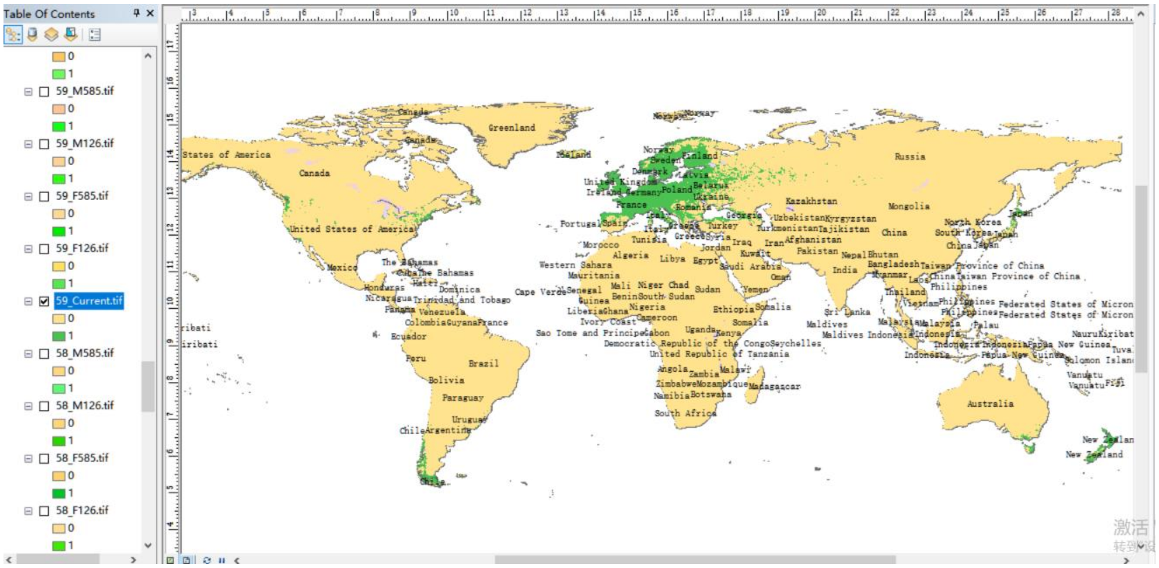

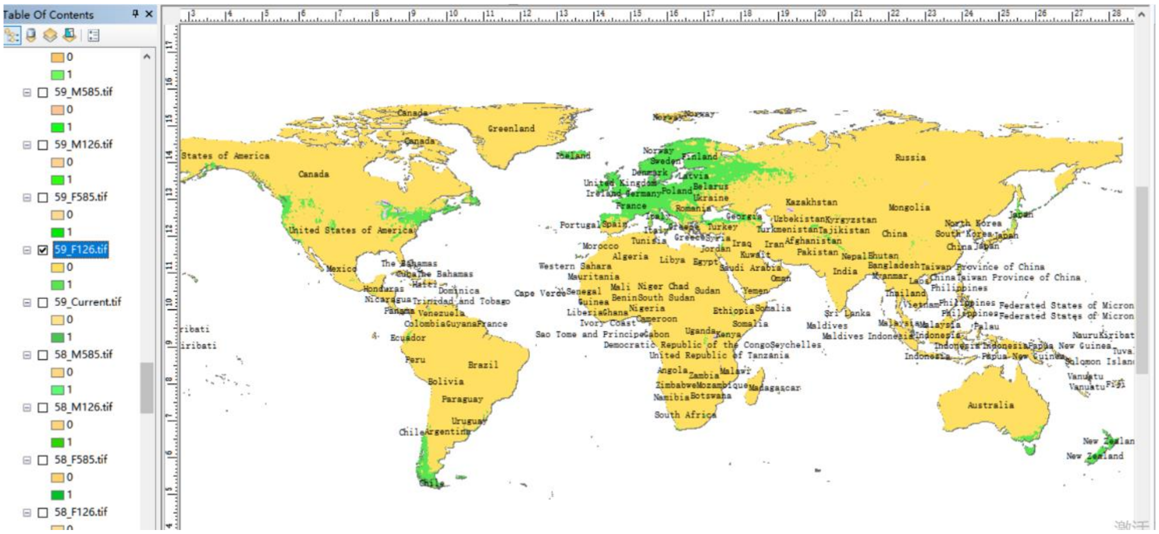

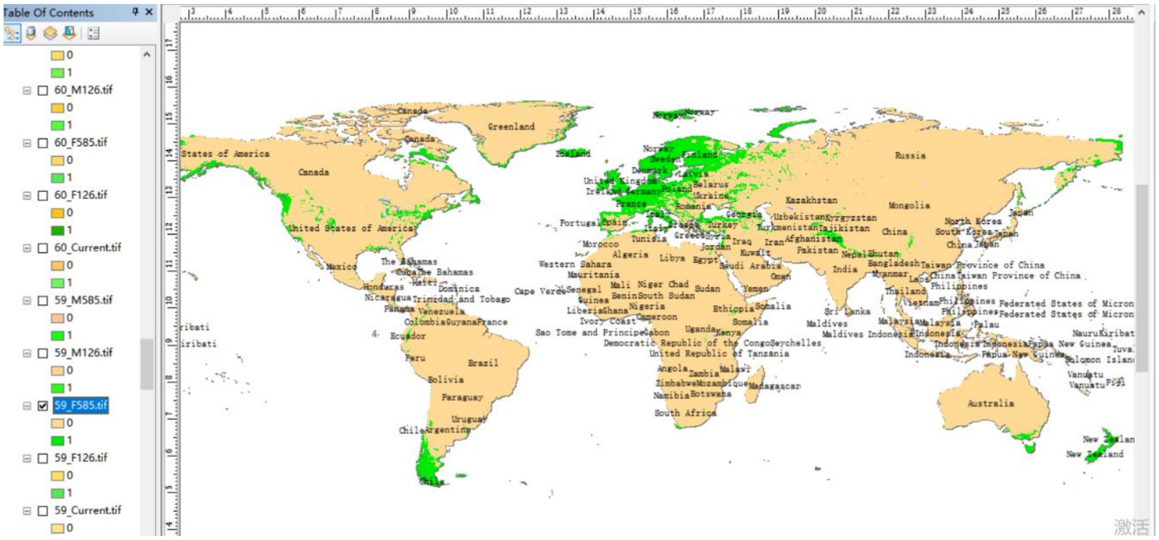
Table Of Contents
0
1
60_M126.tif
0
1
60_F585.tif
0
1
60_F126.tif
0
1
60_Current.tif
0
1
59_M585.tif
0
1
59_M126.tif
0
1
59_F585.tif
0
1
59_F126.tif
0
1
59_Current.tif
0

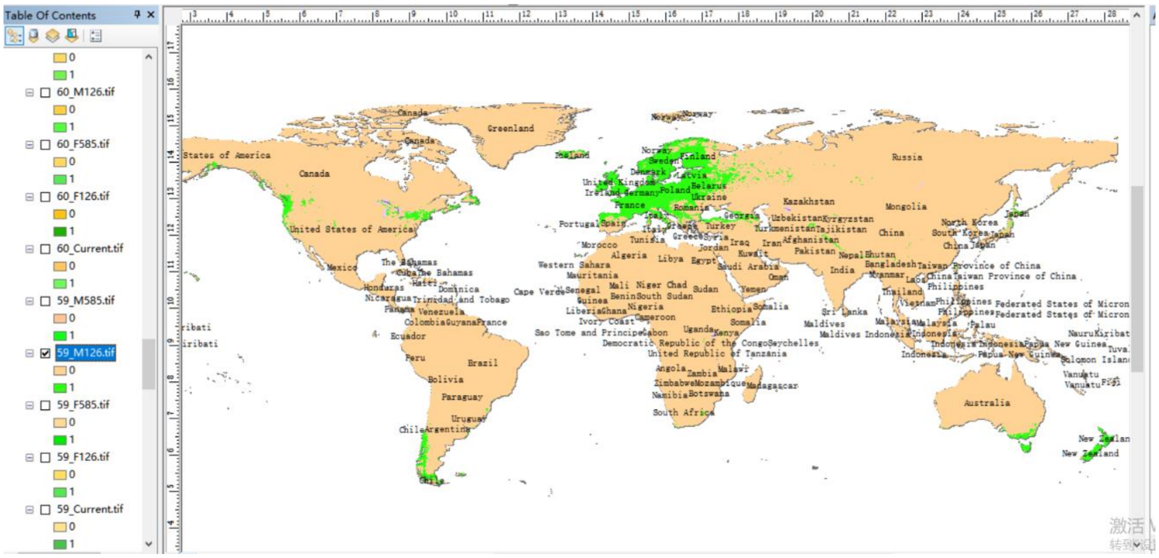
Table Of Contents
0
1
60_M126.tif
0
1
60_F585.tif
0
1
60_F126.tif
0
1
60_Current.tif
0
1
59_M585.tif
0
1
59_M126.tif
0
1
59_F585.tif
0
1
59_F126.tif
0
1
59_Current.tif
0
1

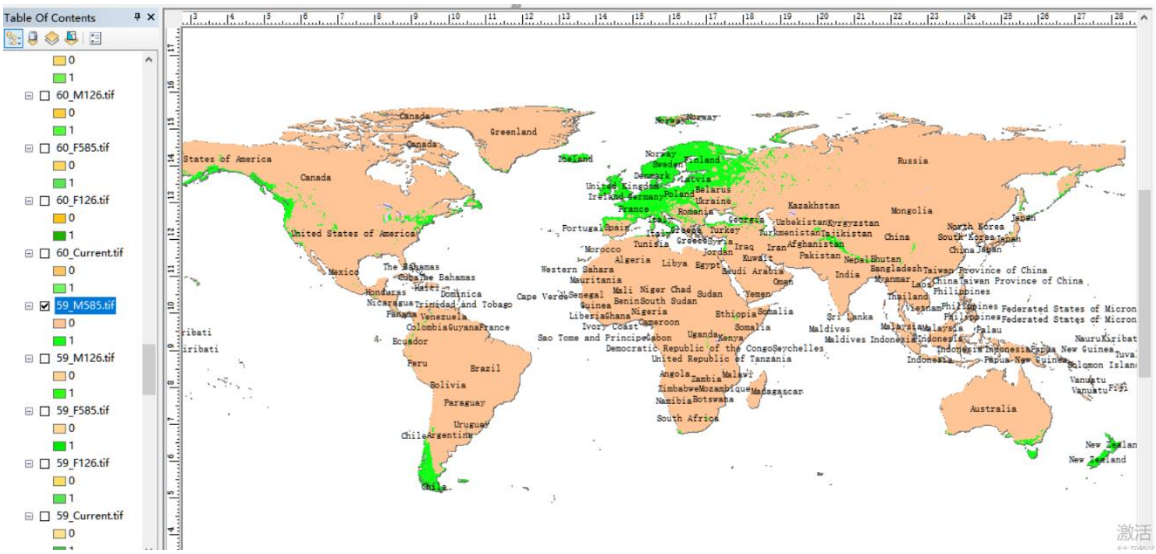
Table Of Contents
0
1
60_M126.tif
0
1
60_F585.tif
0
1
60_F126.tif
0
1
60_Current.tif
0
1
59_M585.tif
0
1
59_M126.tif
0
1
59_F585.tif
0
1
59_F126.tif
0
1
59_Current.tif
0

Anoplolepis gracilipes Current, F126, F585, M126 and M585 in this order

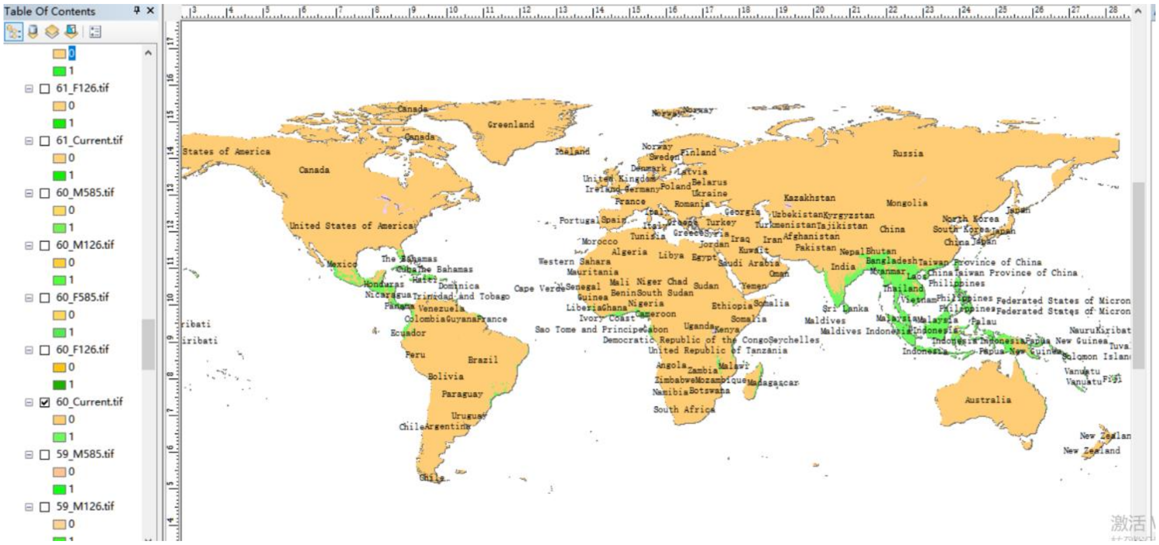

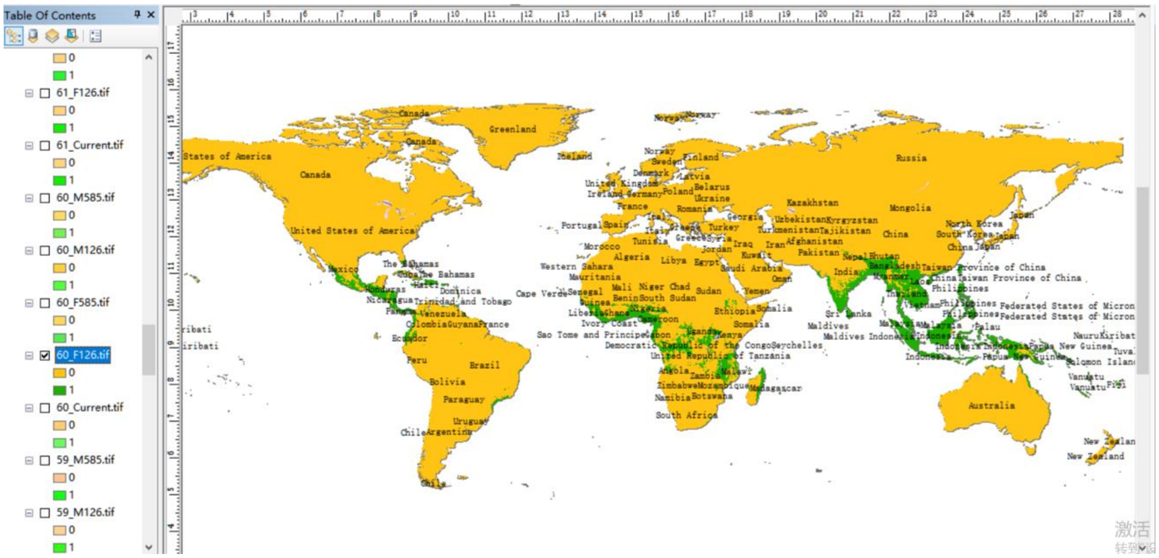

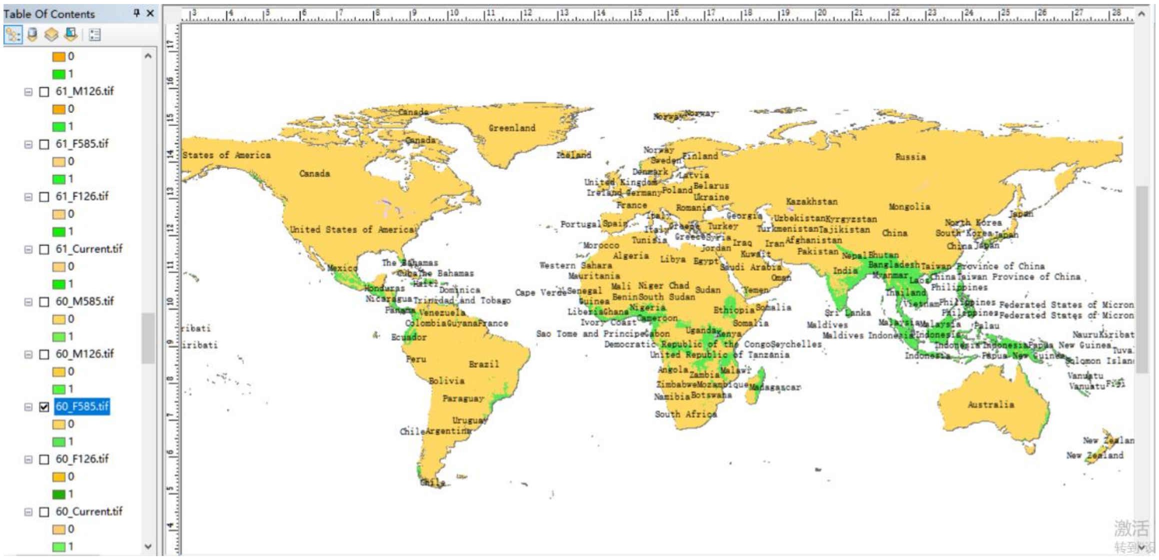
Table Of Contents
0
1
61_M126.tif
0
1
61_F585.tif
0
1
61_F126.tif
0
1
61_Current.tif
0
1
60_M585.tif
0
1
60_M126.tif
0
1
60_F585.tif
0
1
60_F126.tif
0
1
60_Current.tif
0
1
Greenland
Canada
Russia
United States of America
Kazakhstan
Mongolia
China
India
Brazil
Australia
South Africa

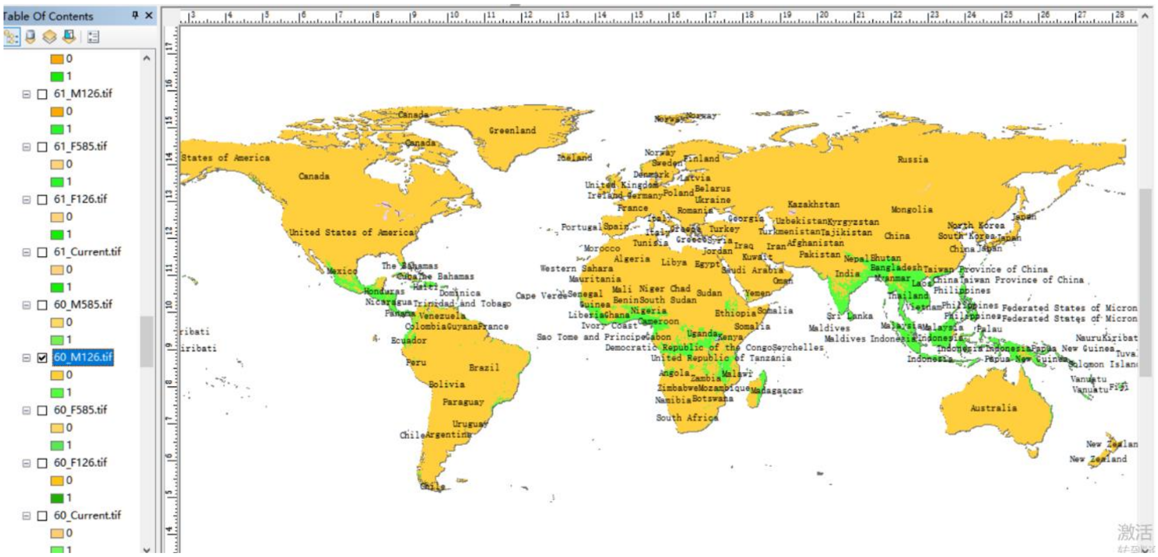
Table Of Contents
0
1
61_M126.tif
0
1
61_F585.tif
0
1
61_F126.tif
0
1
61_Current.tif
0
1
60_M585.tif
0
1
60_M126.tif
0
1
60_F585.tif
0
1
60_F126.tif
0
1
60_Current.tif
0
1
Greenland
Canada
Russia
United States of America
Kazakhstan
Mongolia
China
India
Brazil
Australia
South Africa

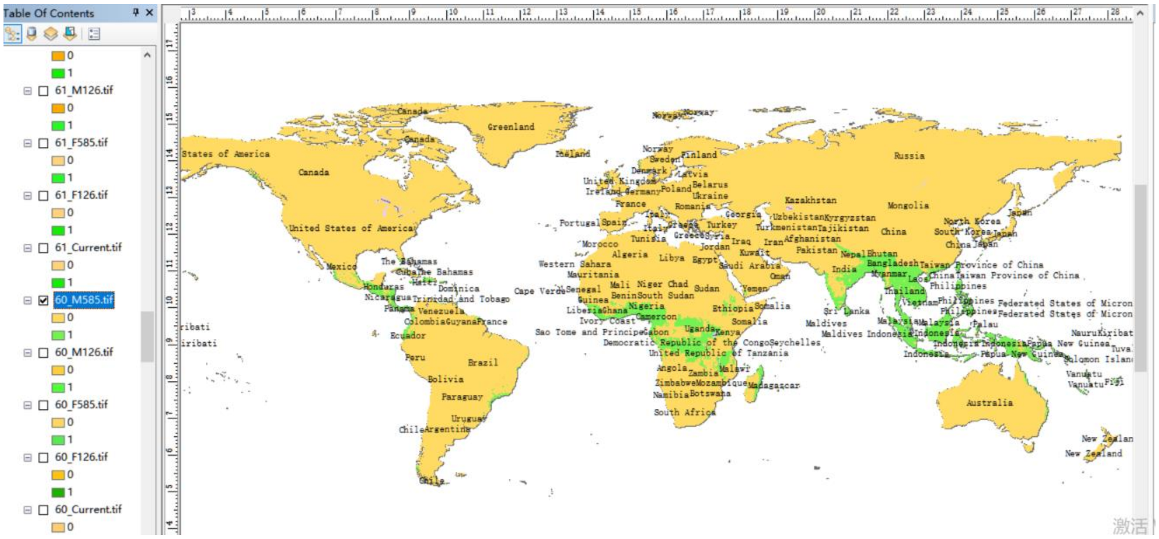
Table Of Contents
0
1
61_M126.tif
0
1
61_F585.tif
0
1
61_F126.tif
0
1
61_Current.tif
0
1
60_M585.tif
0
1
60_M126.tif
0
1
60_F585.tif
0
1
60_F126.tif
0
1
60_Current.tif
0
Greenland
Canada
Russia
United States of America
Kazakhstan
Mongolia
China
India
Brazil
Australia
South Africa

Cinara cupressi   Current, F126, F585, M126 and M585 in this order

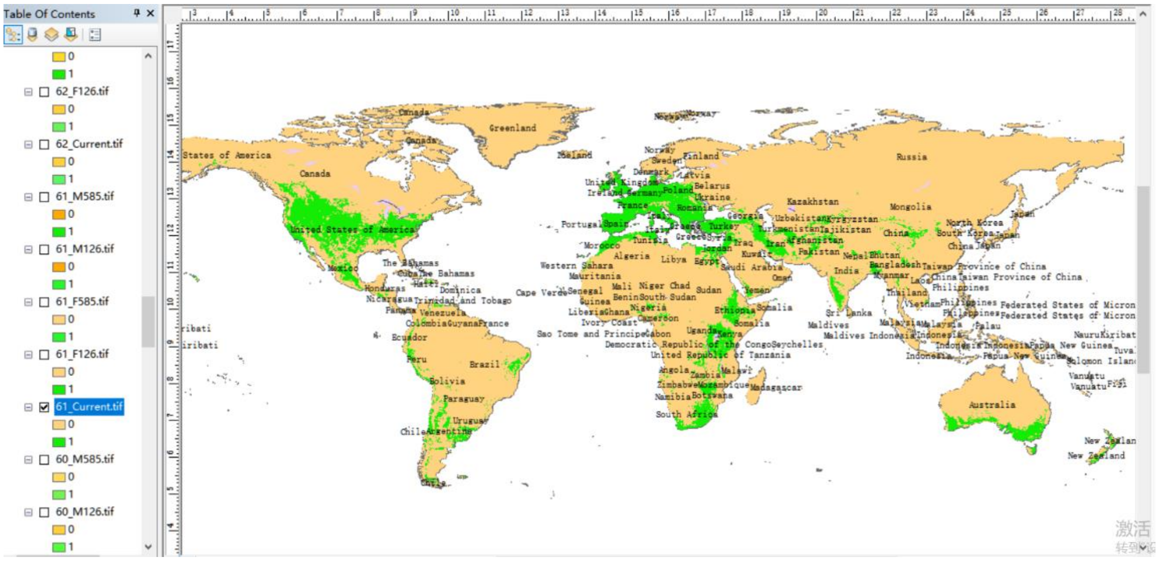

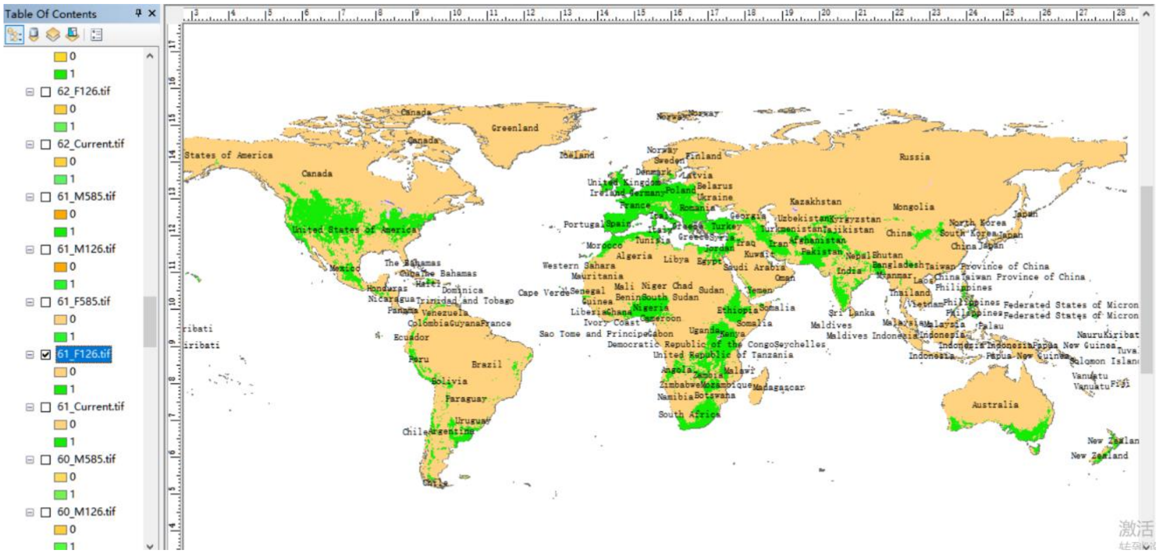

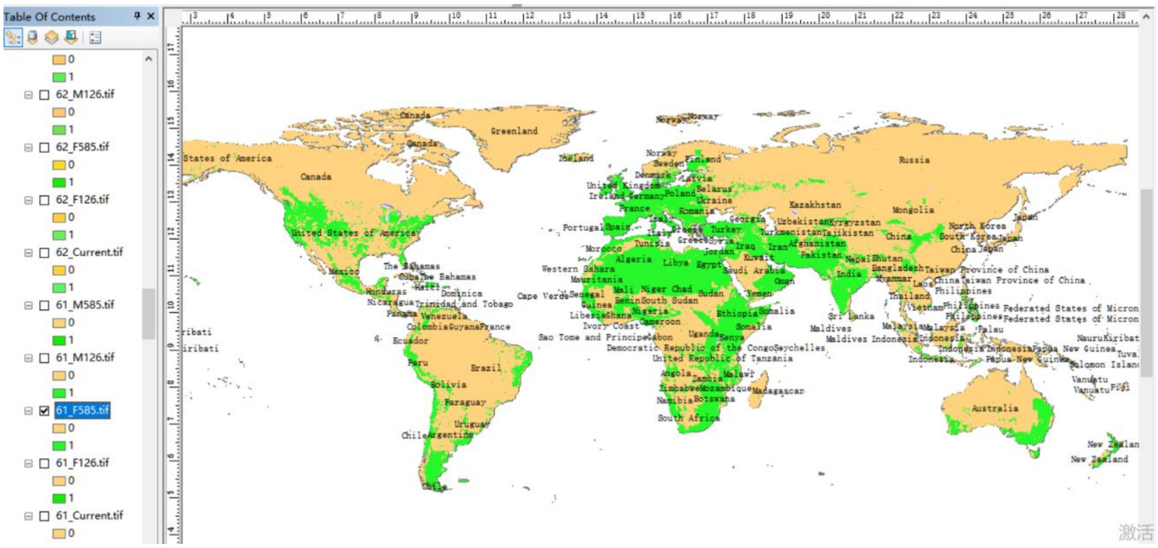
Table Of Contents
0
1
62_M126.tif
0
1
62_F585.tif
0
1
62_F126.tif
0
1
62_Current.tif
0
1
61_M585.tif
0
1
61_M126.tif
0
1
61_F585.tif
0
1
61_F126.tif
0
1
61_Current.tif
0

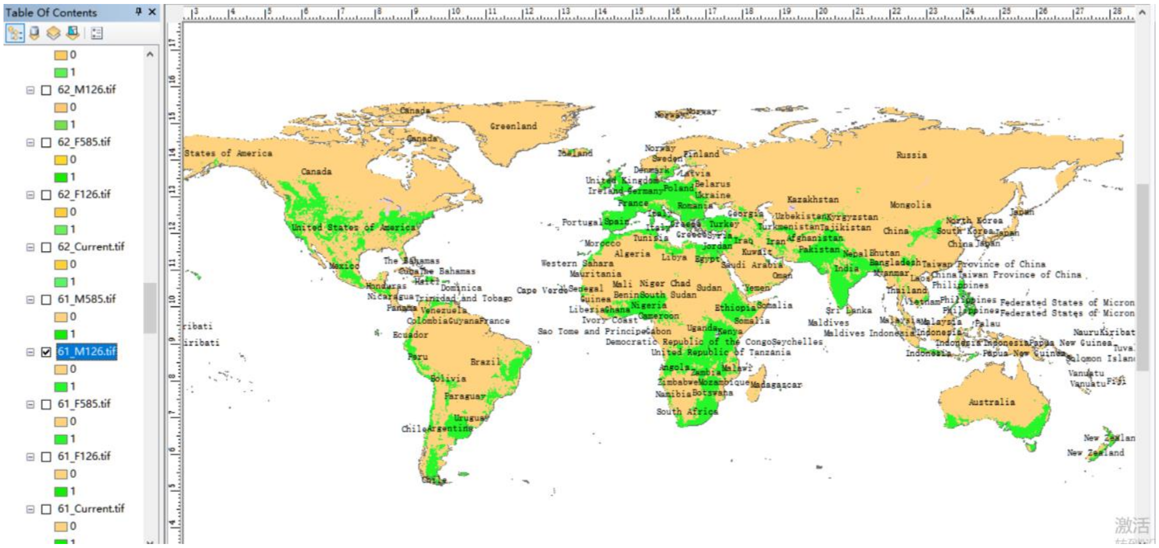
Table Of Contents
0
1
62_M126.tif
0
1
62_F585.tif
0
1
62_F126.tif
0
1
62_Current.tif
0
1
61_M585.tif
0
1
61_M126.tif
0
1
61_F585.tif
0
1
61_F126.tif
0
1
61_Current.tif
0

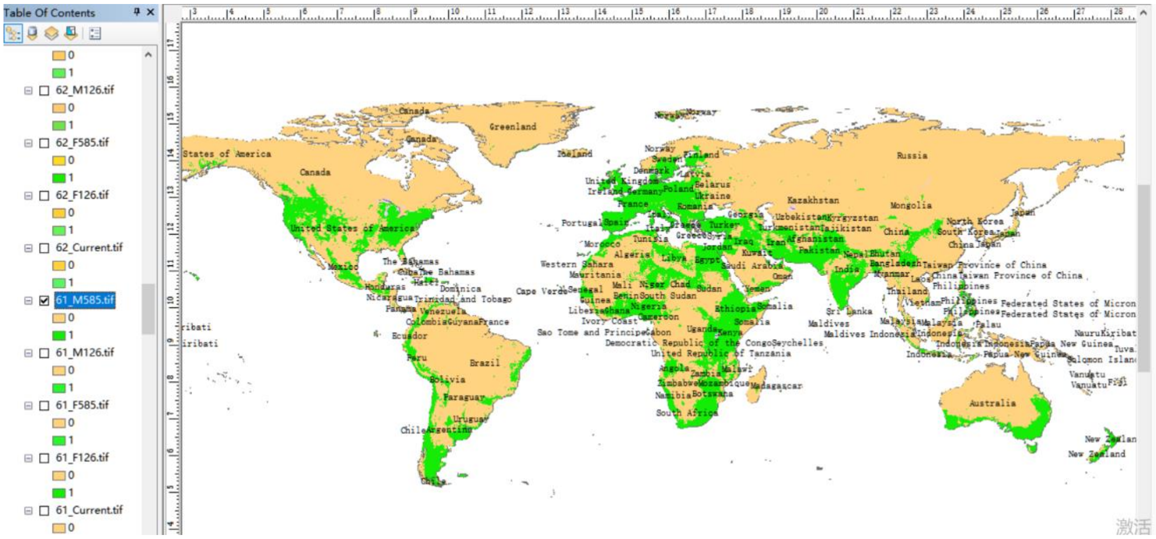
Table Of Contents
0
1
62_M126.tif
0
1
62_F585.tif
0
1
62_F126.tif
0
1
62_Current.tif
0
1
61_M585.tif
0
1
61_M126.tif
0
1
61_F585.tif
0
1
61_F126.tif
0
1
61_Current.tif
0

Platydemus manokwari Current, F126, F585, M126 and M585 in this order

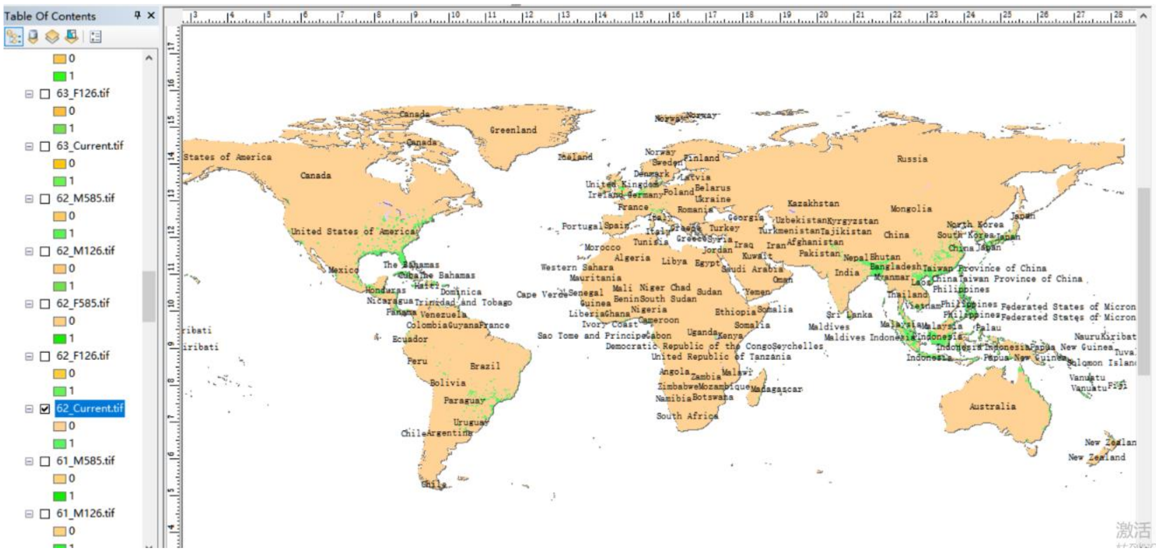

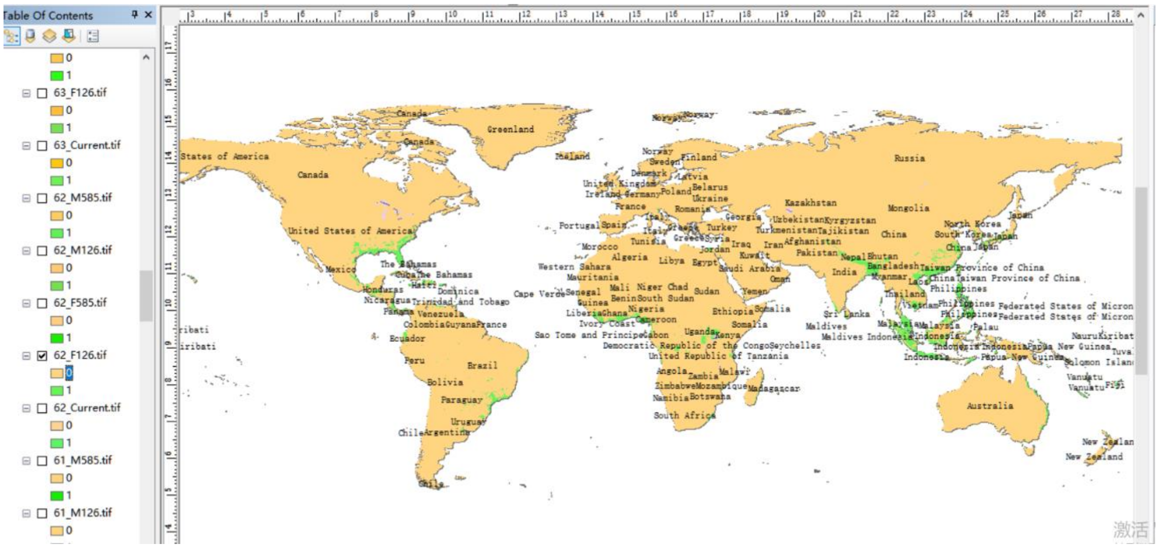

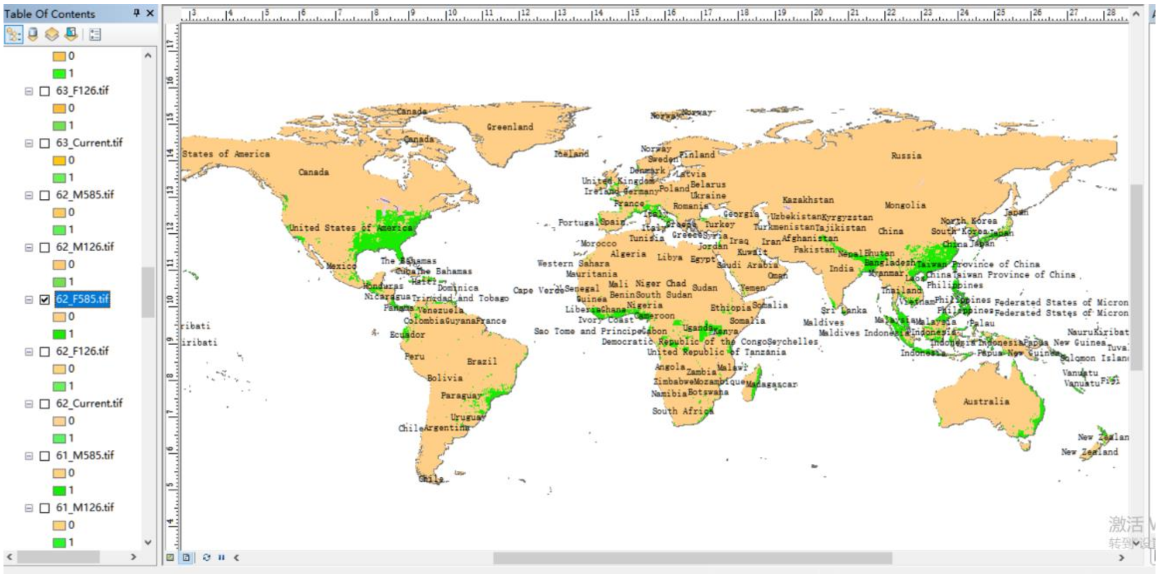
Table Of Contents
63_F126.tif
63_Current.tif
62_M585.tif
62_M126.tif
62_F585.tif
62_F126.tif
62_Current.tif
61_M585.tif
61_M126.tif

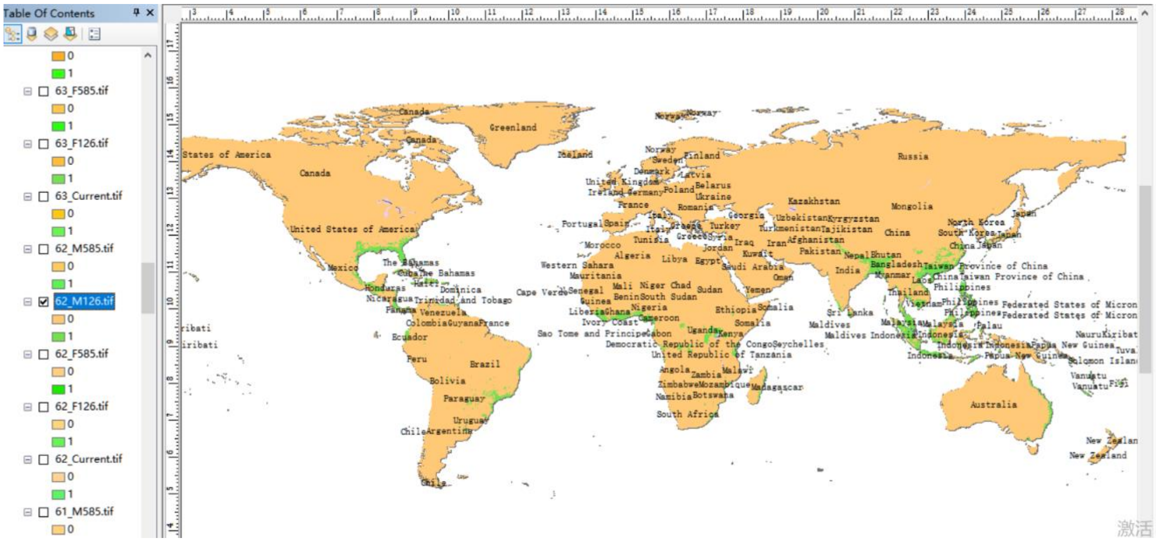
Table Of Contents
63_F585.tif
63_F126.tif
63_Current.tif
62_M585.tif
62_M126.tif
62_F585.tif
62_F126.tif
62_Current.tif
61_M585.tif

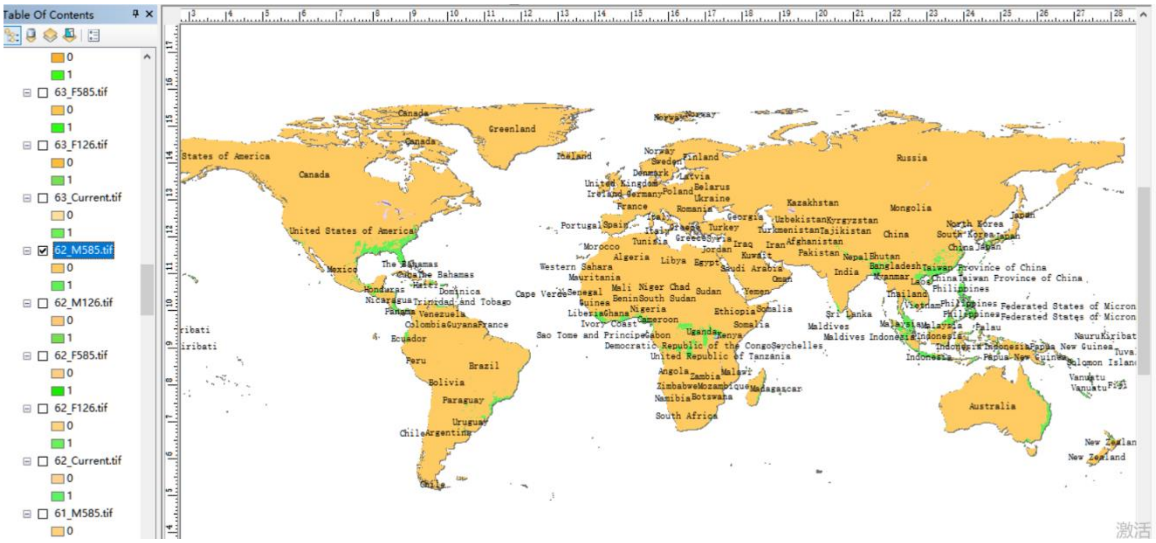
Table Of Contents
63_F585.tif
63_F126.tif
63_Current.tif
62_M585.tif
62_M126.tif
62_F585.tif
62_F126.tif
62_Current.tif
61_M585.tif

Coptotermes formosanus shiraki   Current, F126, F585, M126 and M585 in this order

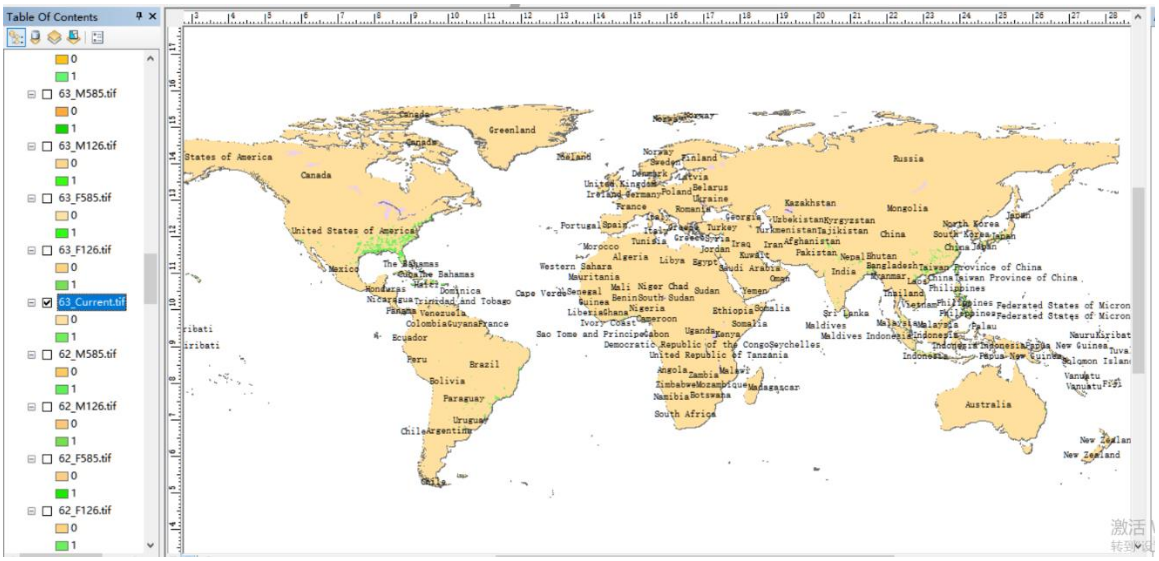

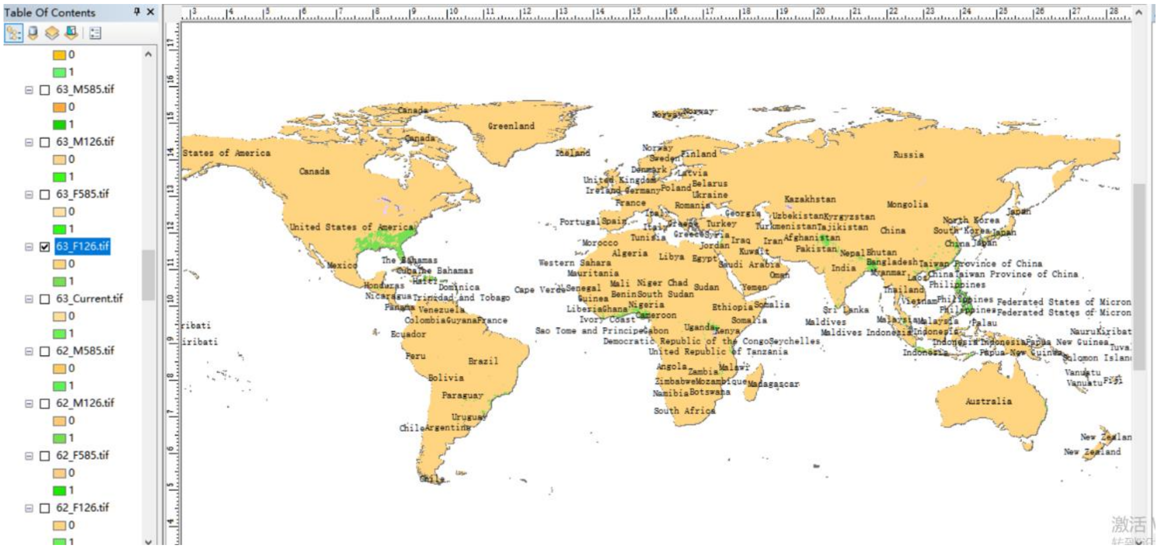

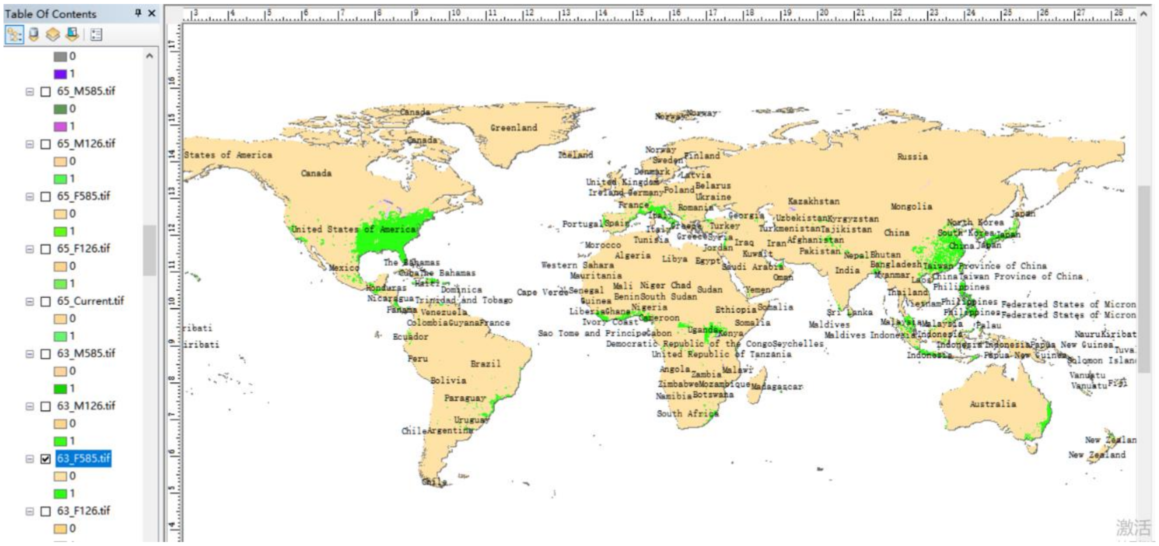
Table Of Contents
0
1
65_M585.tif
0
1
65_M126.tif
0
1
65_F585.tif
0
1
65_F126.tif
0
1
65_Current.tif
0
1
63_M585.tif
0
1
63_M126.tif
0
1
63_F585.tif
0
1
63_F126.tif
0

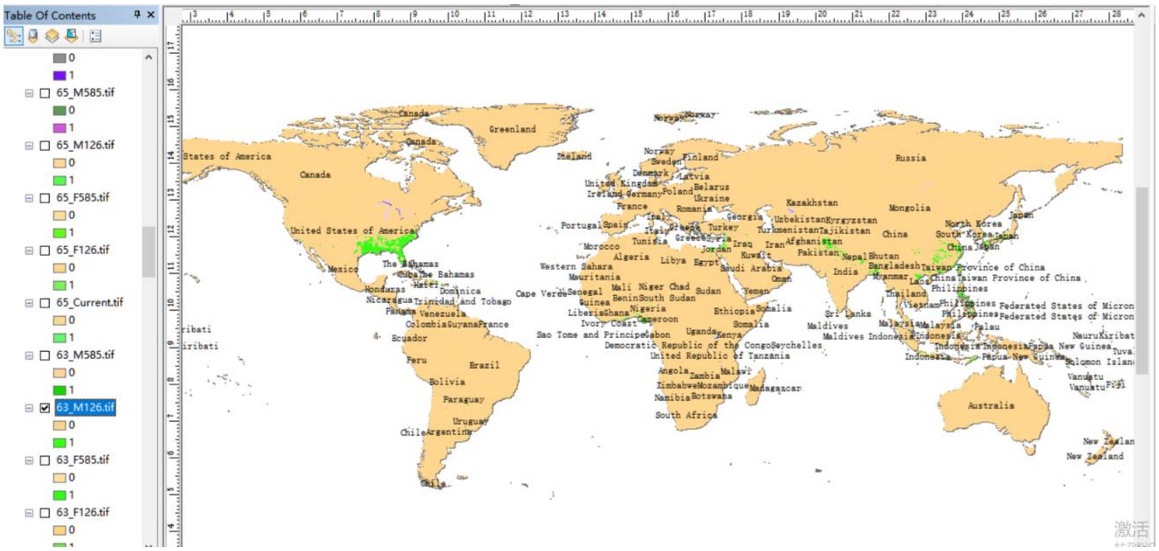
Table Of Contents
0
1
65_M585.tif
0
1
65_M126.tif
0
1
65_F585.tif
0
1
65_F126.tif
0
1
65_Current.tif
0
1
63_M585.tif
0
1
63_M126.tif
0
1
63_F585.tif
0
1
63_F126.tif
0

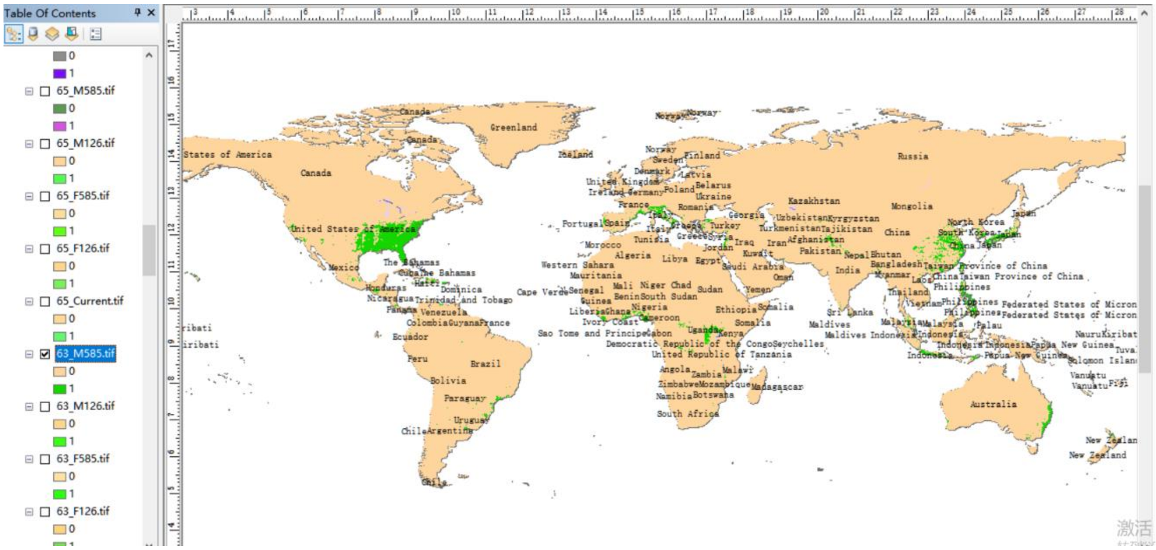
Table Of Contents
0
1
65_M585.tif
0
1
65_M126.tif
0
1
65_F585.tif
0
1
65_F126.tif
0
1
65_Current.tif
0
1
63_M585.tif
0
1
63_M126.tif
0
1
63_F585.tif
0
1
63_F126.tif
0

Lymantria dispar   Current, F126, F585, M126 and M585 in this order

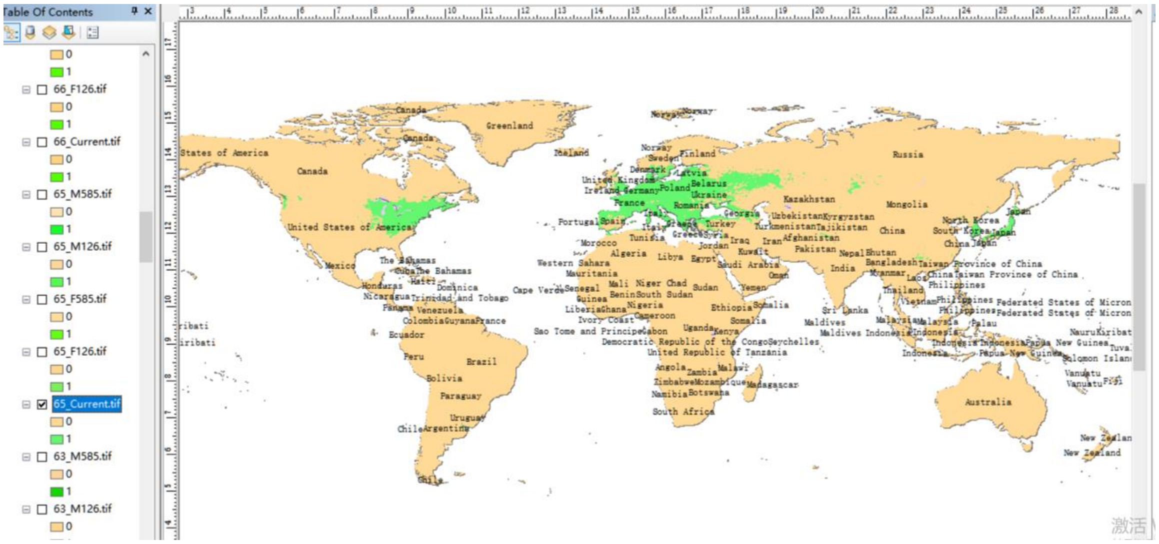

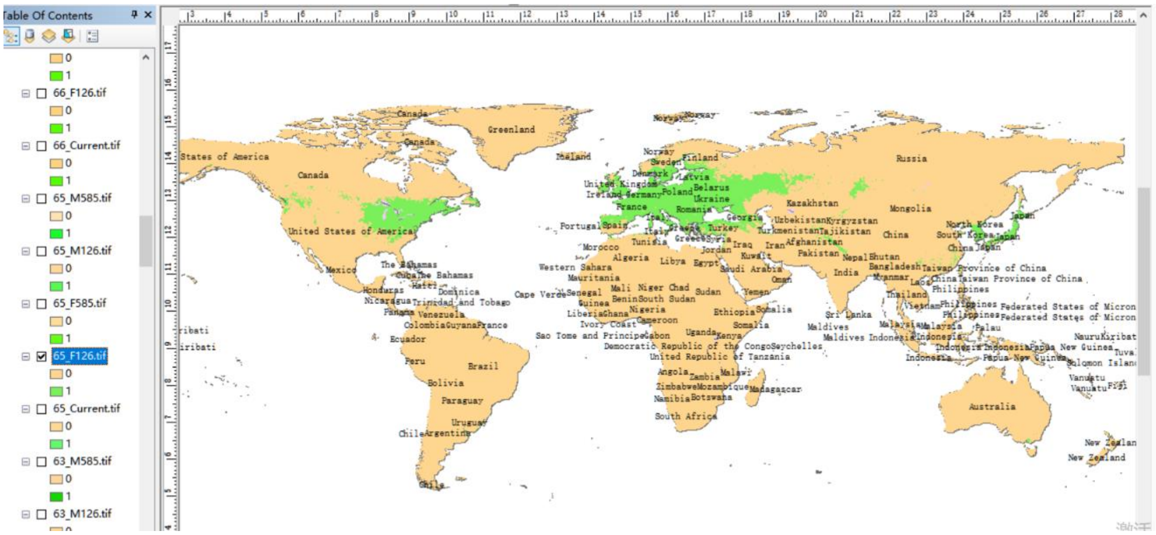

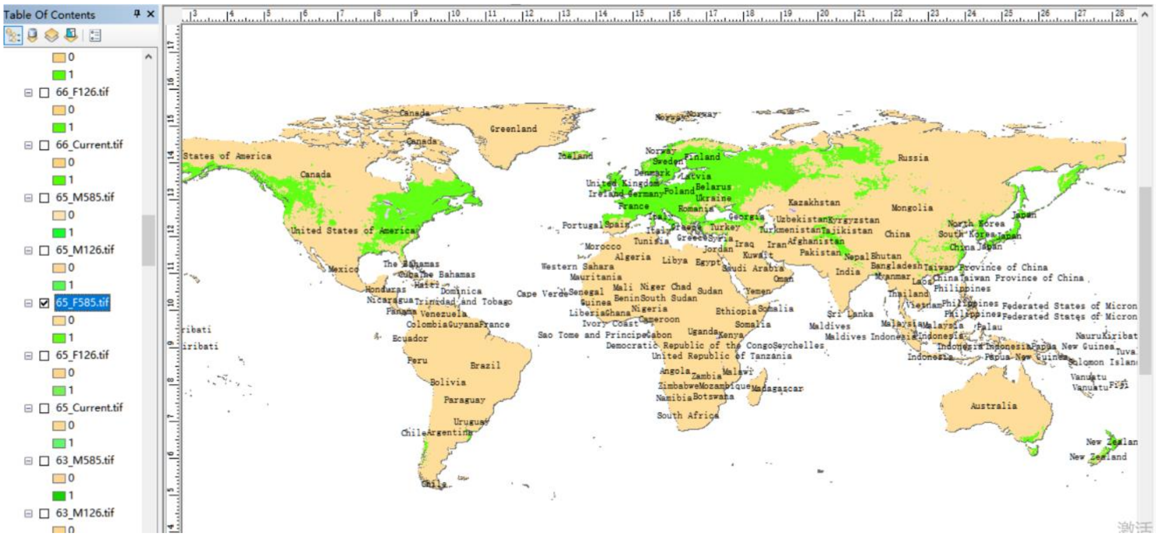
Table Of Contents
66_F126.tif
66_Current.tif
65_M585.tif
65_M126.tif
65_F585.tif
65_F126.tif
65_Current.tif
63_M585.tif
63_M126.tif

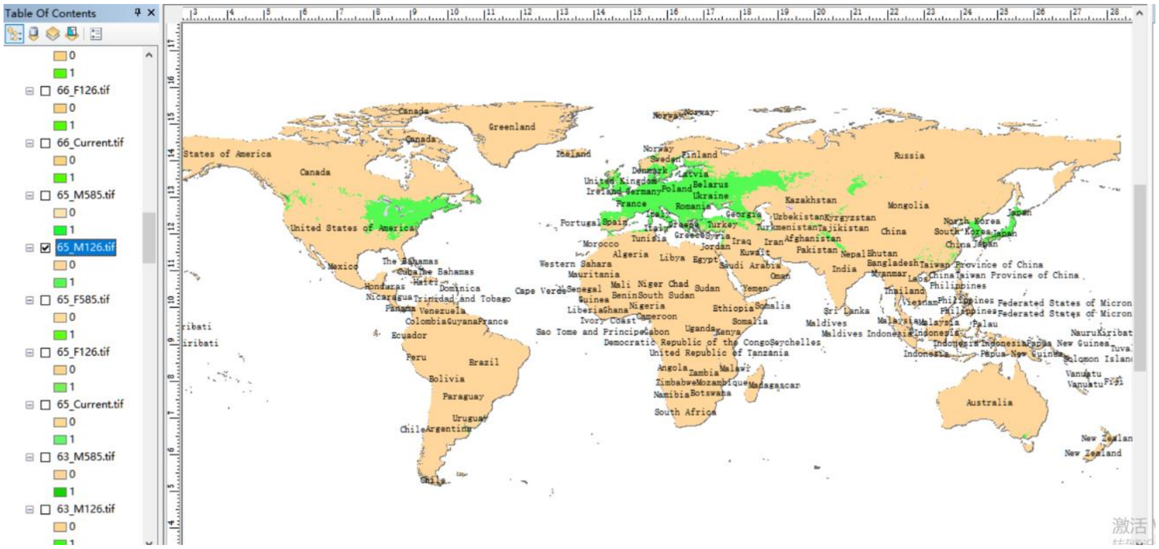
Table Of Contents
66_F126.tif
66_Current.tif
65_M585.tif
65_M126.tif
65_F585.tif
65_F126.tif
65_Current.tif
63_M585.tif
63_M126.tif

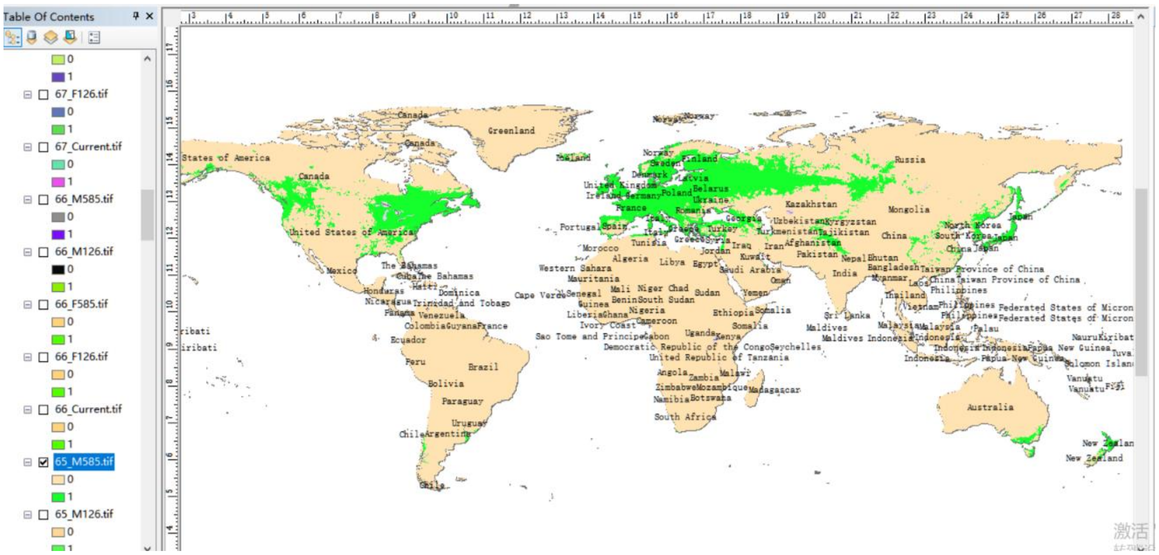
Table Of Contents
67_F126.tif
67_Current.tif
66_M585.tif
66_M126.tif
66_F585.tif
66_F126.tif
66_Current.tif
65_M585.tif
65_M126.tif

Trogoderma granarium Current, F126, F585, M126 and M585 in this order

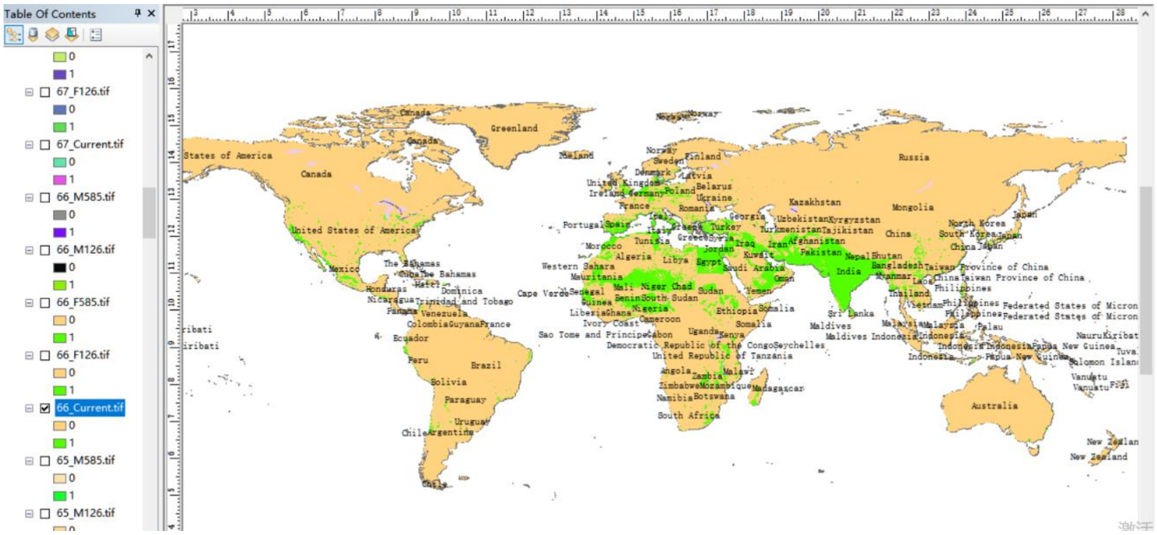

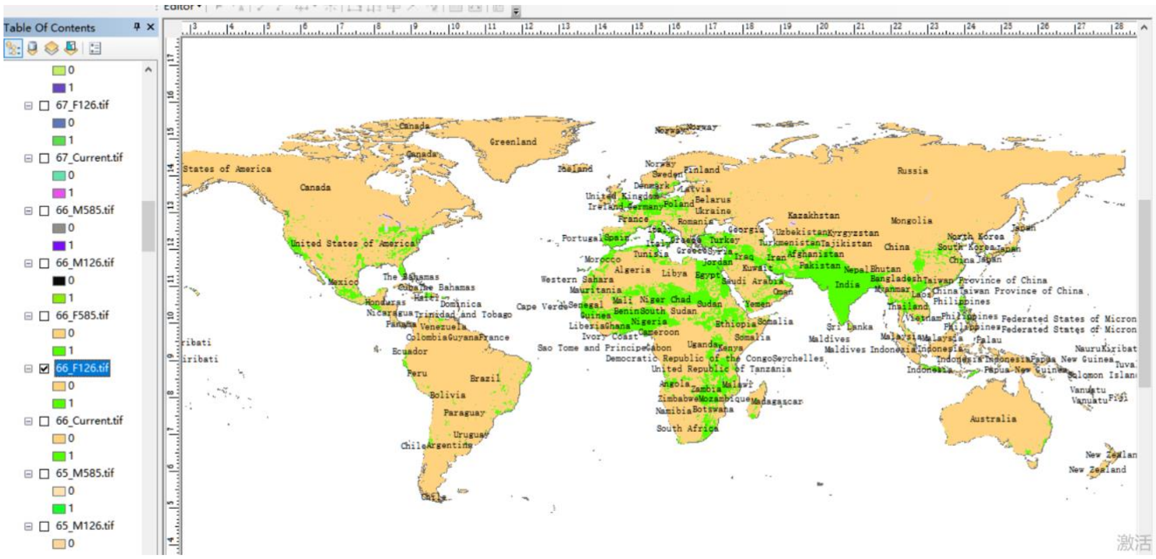

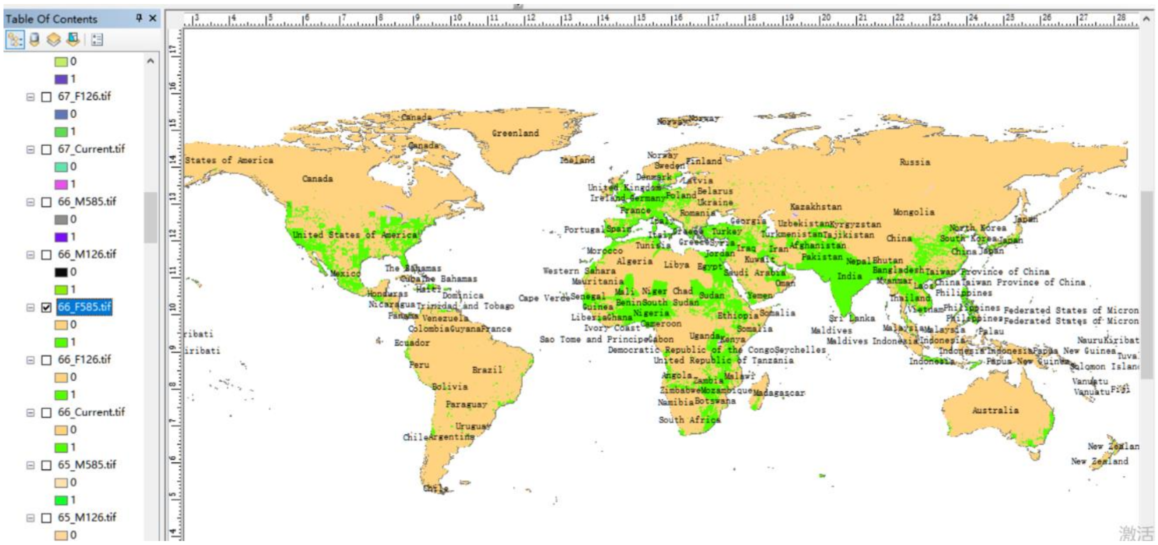
Table Of Contents
67_F126.tif
67_Current.tif
66_M585.tif
66_M126.tif
66_F585.tif
66_F126.tif
66_Current.tif
65_M585.tif
65_M126.tif

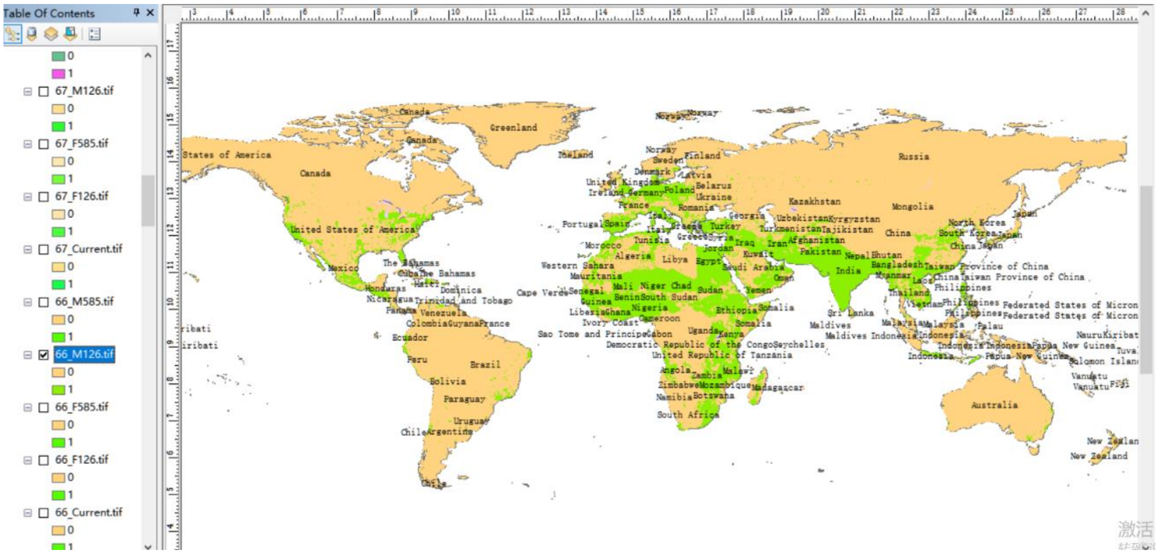
Table Of Contents
67_M126.tif
67_F585.tif
67_F126.tif
67_Current.tif
66_M585.tif
66_M126.tif
66_F585.tif
66_F126.tif
66_Current.tif

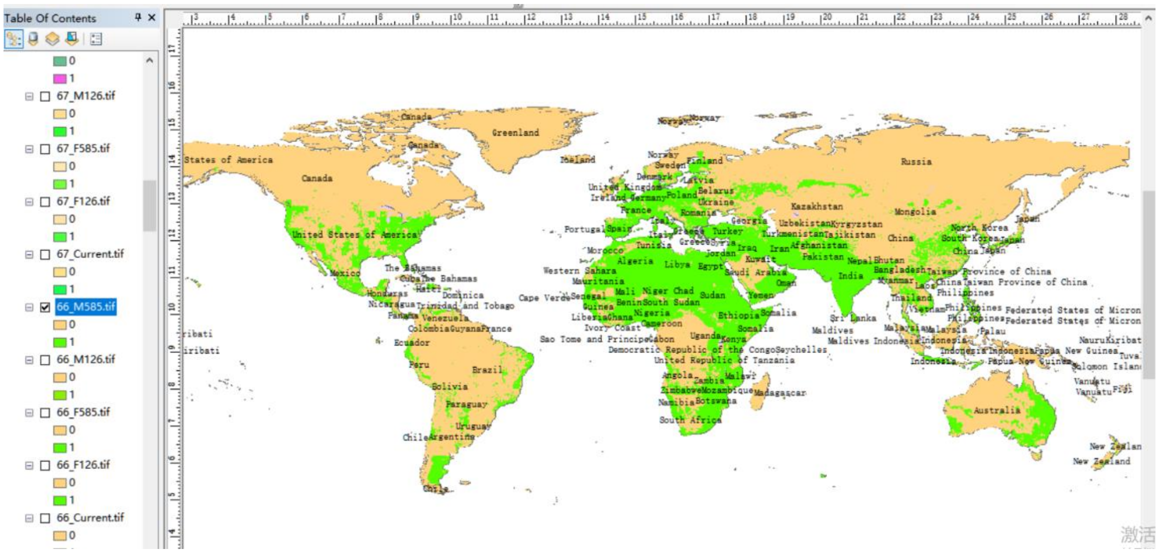
Table Of Contents
67_M126.tif
67_F585.tif
67_F126.tif
67_Current.tif
66_M585.tif
66_M126.tif
66_F585.tif
66_F126.tif
66_Current.tif

Wasmannia auropunctata Current, F126, F585, M126 and M585 in this order

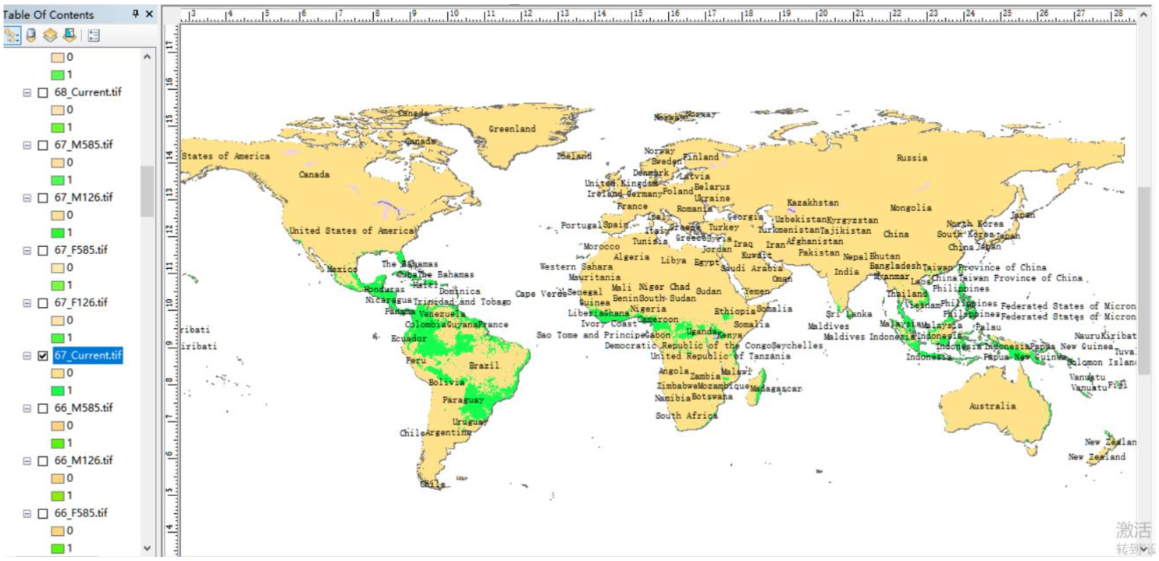

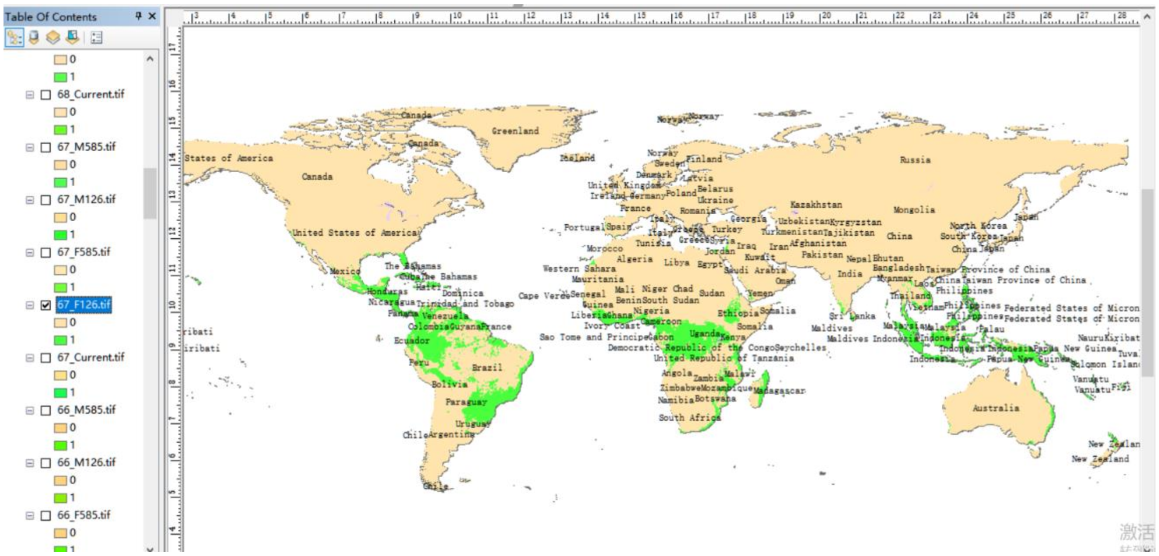

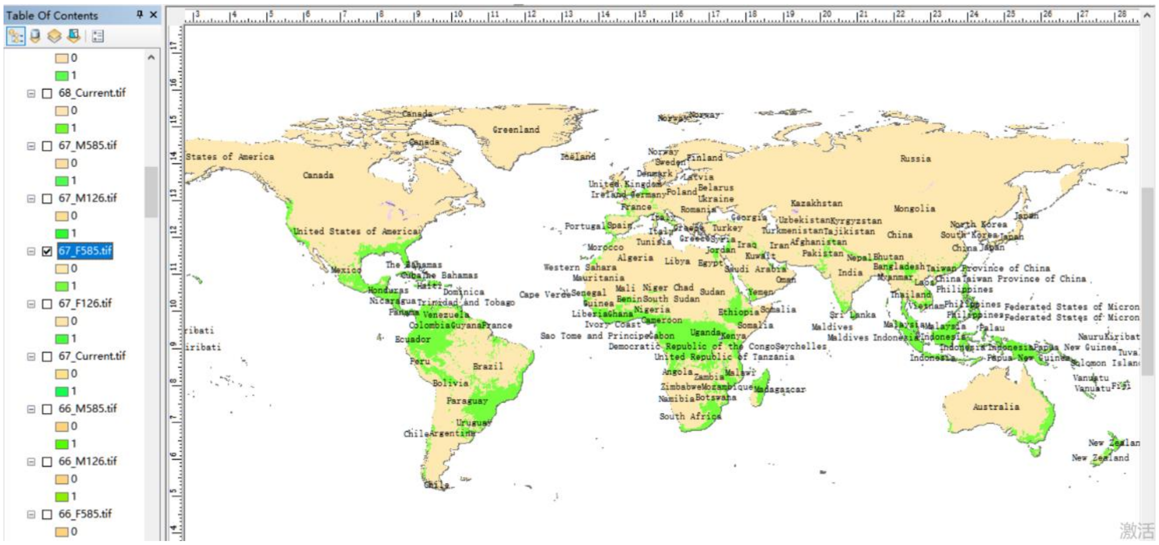
Table Of Contents
0
1
68_Current.tif
0
1
67_M585.tif
0
1
67_M126.tif
0
1
67_F585.tif
0
1
67_F126.tif
0
1
67_Current.tif
0
1
66_M585.tif
0
1
66_M126.tif
0
1
66_F585.tif
0

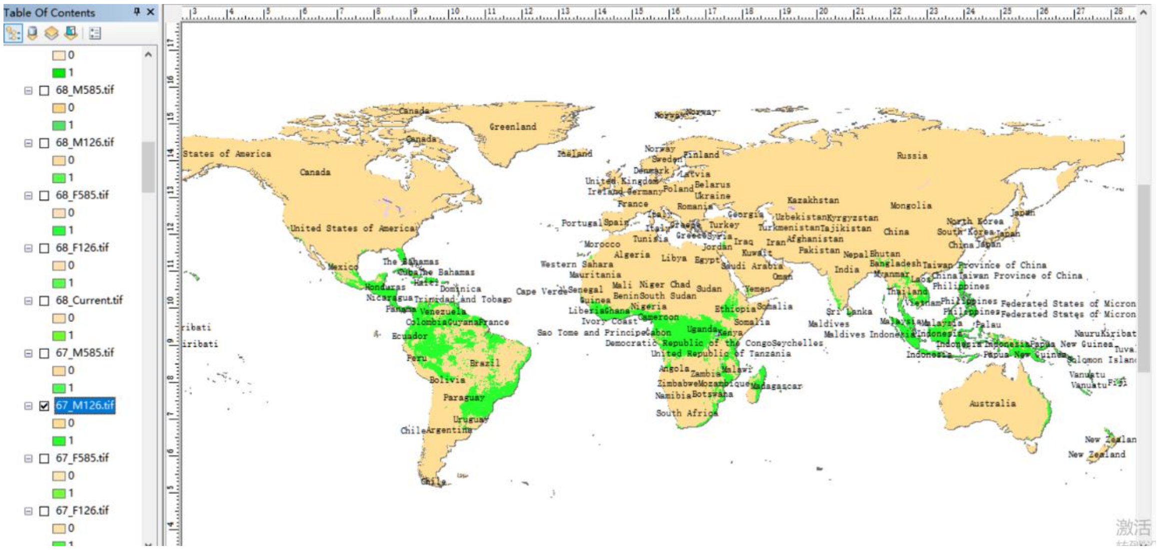
Table Of Contents
0
1
68_M585.tif
0
1
68_M126.tif
0
1
68_F585.tif
0
1
68_F126.tif
0
1
68_Current.tif
0
1
67_M585.tif
0
1
67_M126.tif
0
1
67_F585.tif
0
1
67_F126.tif
0

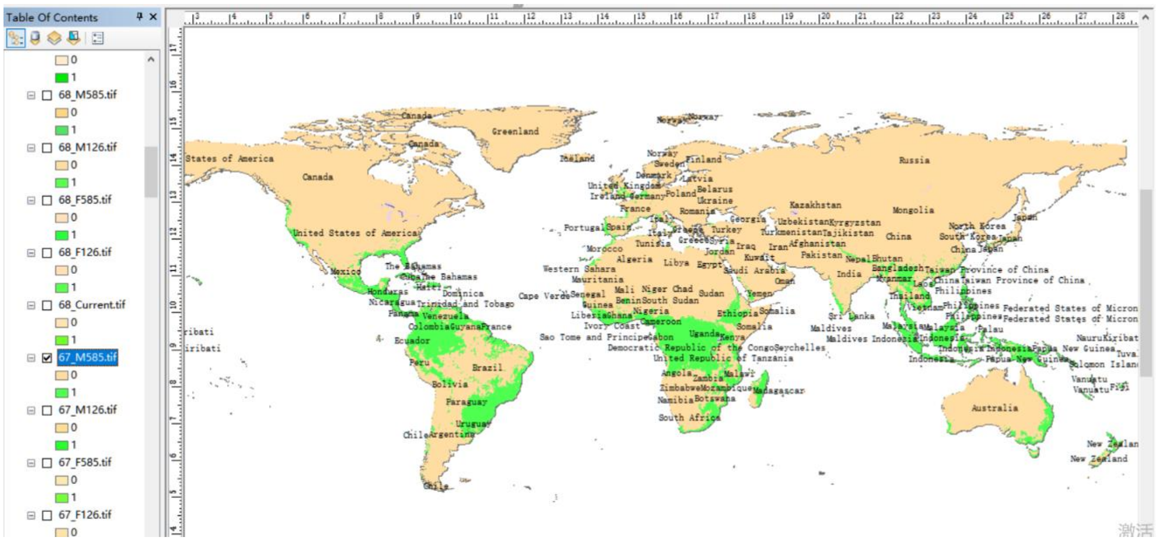
Table Of Contents
0
1
68_M585.tif
0
1
68_M126.tif
0
1
68_F585.tif
0
1
68_F126.tif
0
1
68_Current.tif
0
1
67_M585.tif
0
1
67_M126.tif
0
1
67_F585.tif
0
1
67_F126.tif
0

Solenopsis invicta Current, F126, F585, M126 and M585 in this order

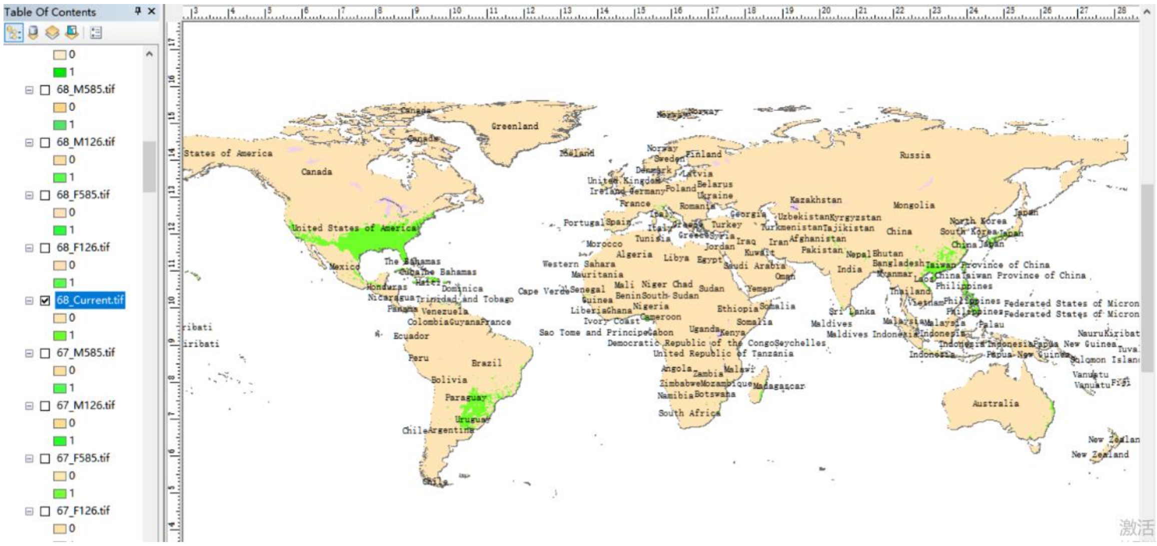

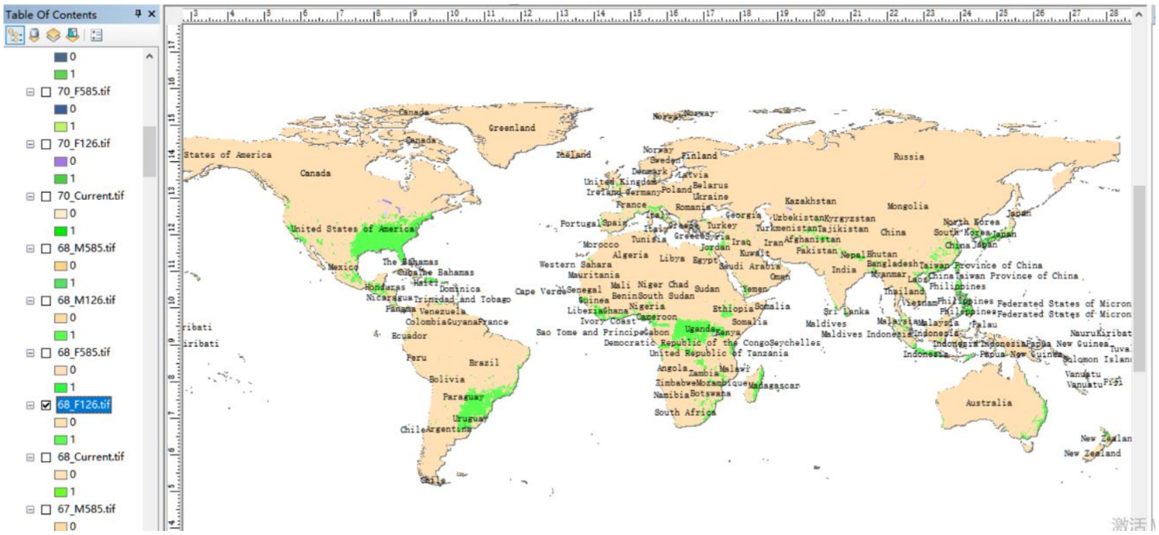

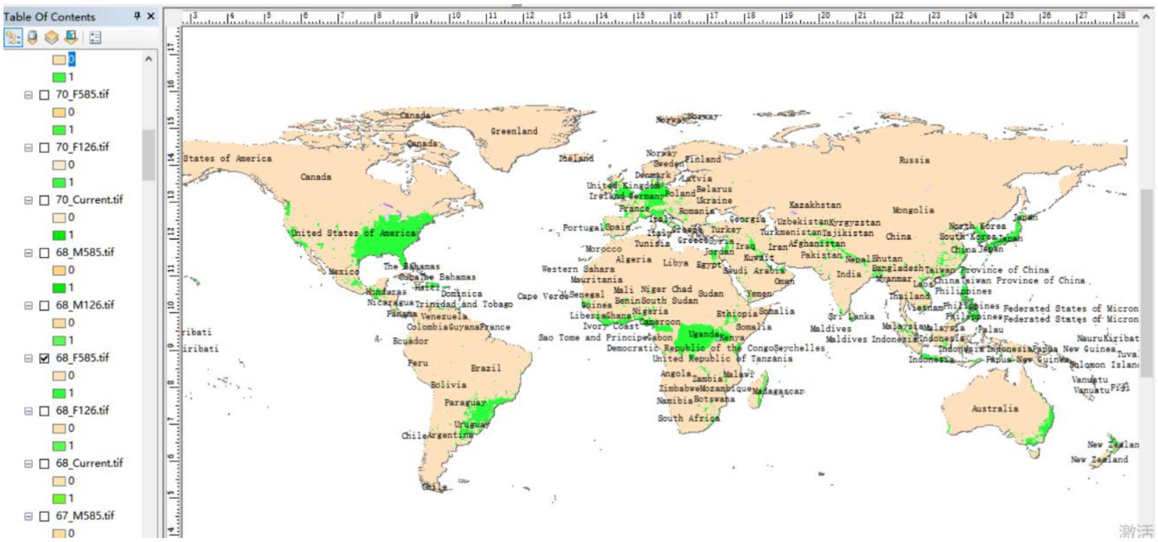
Table Of Contents
0
1
70_F585.tif
0
1
70_F126.tif
0
1
70_Current.tif
0
1
68_M585.tif
0
1
68_M126.tif
0
1
68_F585.tif
0
1
68_F126.tif
0
1
68_Current.tif
0
1
67_M585.tif
0
激活

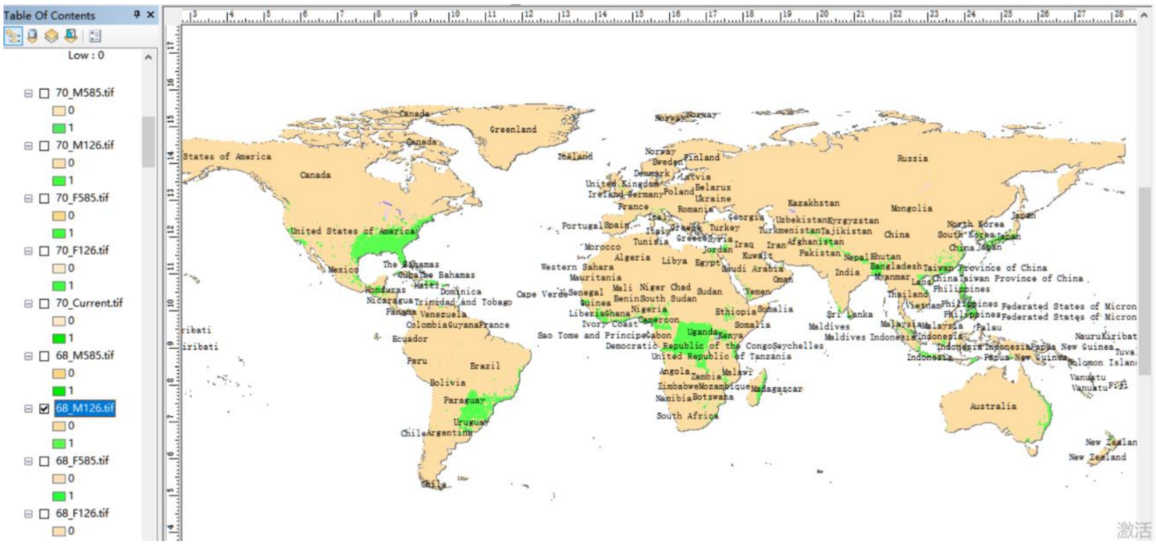
Table Of Contents
Low : 0
70_M585.tif
0
1
70_M126.tif
0
1
70_F585.tif
0
1
70_F126.tif
0
1
70_Current.tif
0
1
68_M585.tif
0
1
68_M126.tif
0
1
68_F585.tif
0
1
68_F126.tif
0
激活

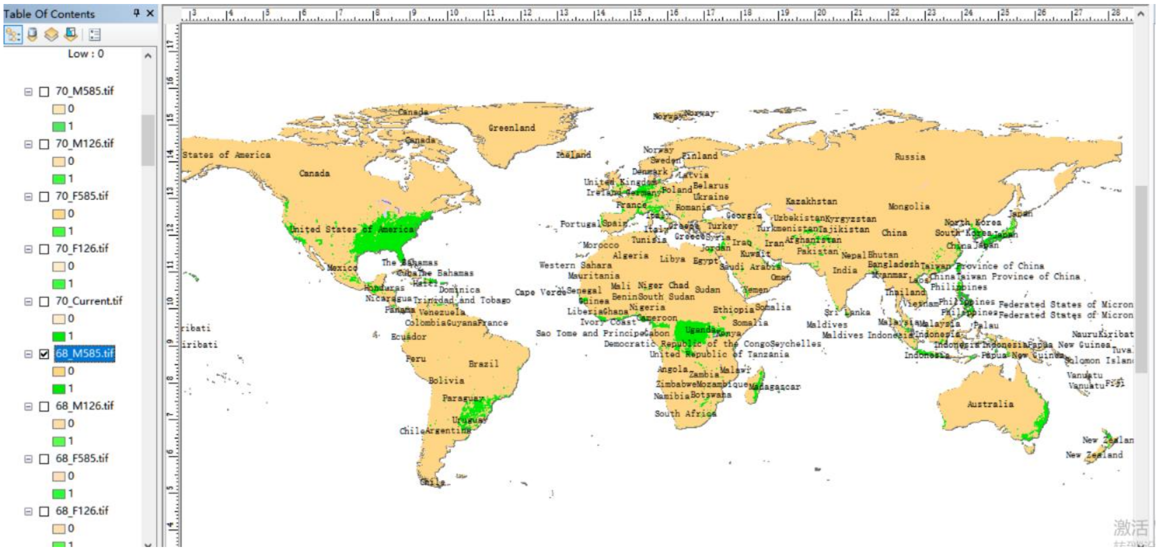
Table Of Contents
Low : 0
70_M585.tif
0
1
70_M126.tif
0
1
70_F585.tif
0
1
70_F126.tif
0
1
70_Current.tif
0
1
68_M585.tif
0
1
68_M126.tif
0
1
68_F585.tif
0
1
68_F126.tif
0
激活

Bemisia tabaci Current, F126, F585, M126 and M585 in this order

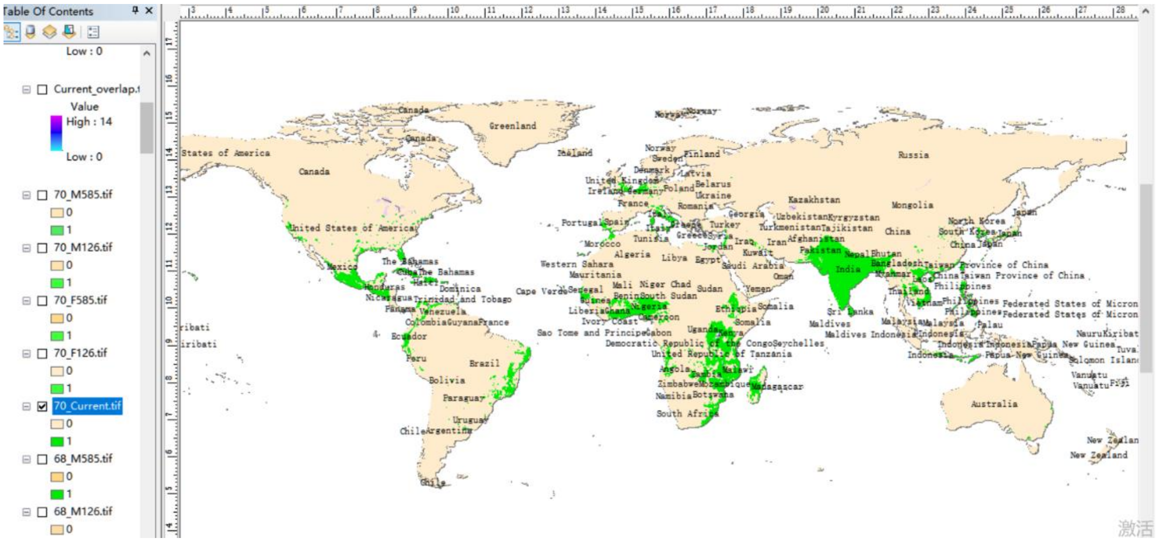

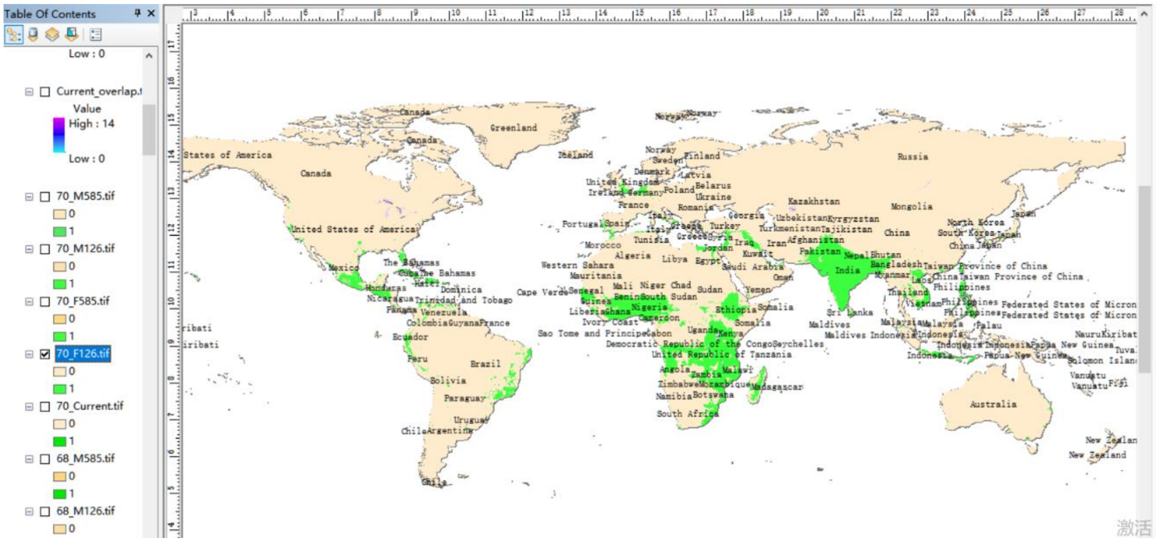

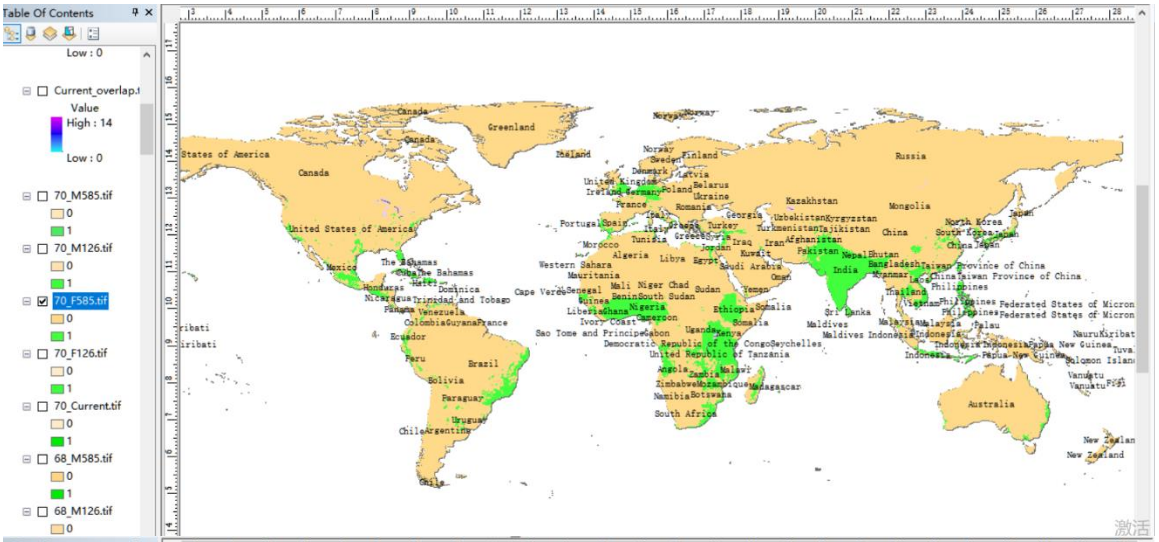
Table Of Contents
Low : 0
Current_overlap.t
Value
High : 14
Low : 0
70_M585.tif
0
1
70_M126.tif
0
1
70_F585.tif
0
1
70_F126.tif
0
1
70_Current.tif
0
1
68_M585.tif
0
1
68_M126.tif
0
激活

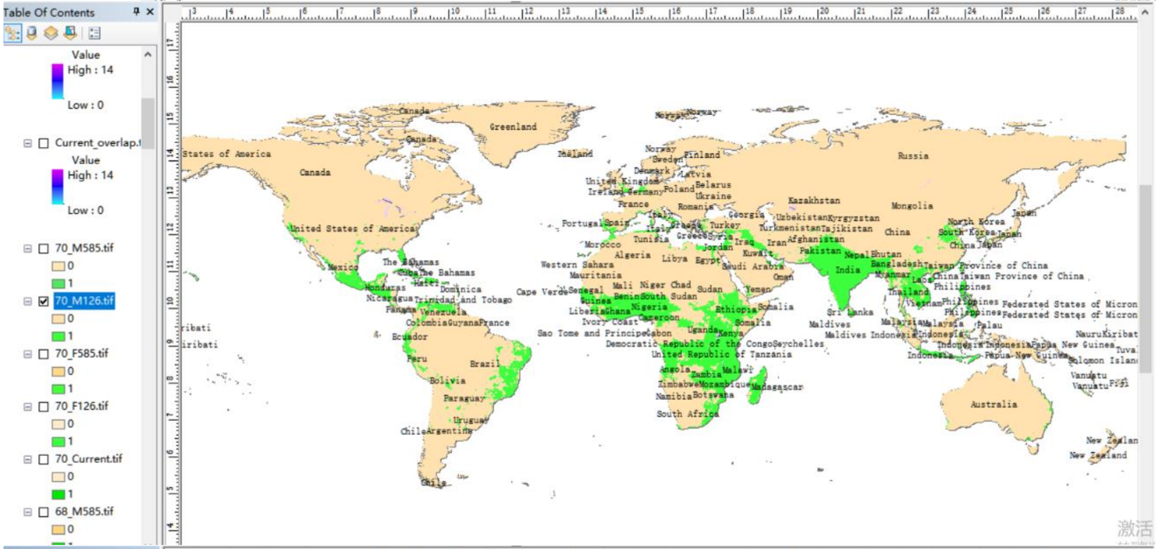
Table Of Contents
Value
High : 14
Low : 0
Current_overlap.t
Value
High : 14
Low : 0
70_M585.tif
0
1
70_M126.tif
0
1
70_F585.tif
0
1
70_F126.tif
0
1
70_Current.tif
0
1
68_M585.tif
0
激活

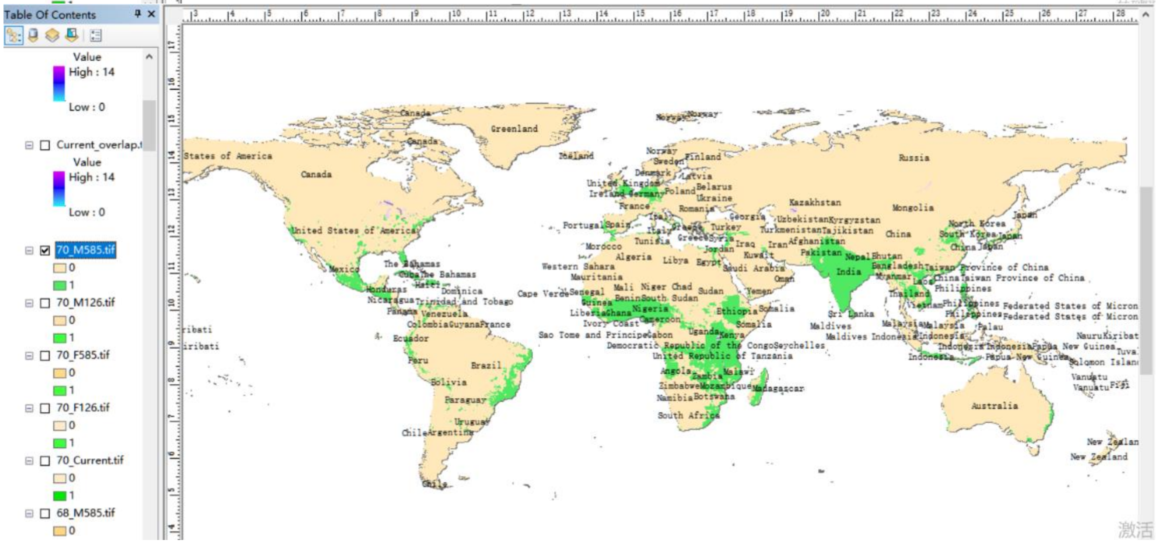
Table Of Contents
Value
High : 14
Low : 0
Current_overlap.t
Value
High : 14
Low : 0
70_M585.tif
0
1
70_M126.tif
0
1
70_F585.tif
0
1
70_F126.tif
0
1
70_Current.tif
0
1
68_M585.tif
0
激活
